# Reconstructing vegetation biomass in the Middle Jurassic Yanliao Biota from insect fossil assemblages

**DOI:** 10.1093/nsr/nwag329

**Published:** 2026-06-02

**Authors:** Liang Chen, Shilong Guo, Lifang Xiao, Nan Yang, Chungkun Shih, Conrad C Labandeira, Chaofan Shi, Dong Ren

**Affiliations:** College of Life Sciences, Capital Normal University, Beijing 100048, China; Department of Paleobiology, National Museum of Natural History, Smithsonian Institution, WA, DC 20013-7012, USA; College of Life Sciences, Capital Normal University, Beijing 100048, China; Department of Paleobiology, National Museum of Natural History, Smithsonian Institution, WA, DC 20013-7012, USA; Department of Paleobiology, National Museum of Natural History, Smithsonian Institution, WA, DC 20013-7012, USA; Institute of Zoology, Guangdong Academy of Science, Guangzhou 510260, China; College of Life Sciences, Capital Normal University, Beijing 100048, China; College of Life Sciences, Capital Normal University, Beijing 100048, China; Department of Paleobiology, National Museum of Natural History, Smithsonian Institution, WA, DC 20013-7012, USA; College of Life Sciences, Capital Normal University, Beijing 100048, China; Department of Paleobiology, National Museum of Natural History, Smithsonian Institution, WA, DC 20013-7012, USA; Department of Entomology, University of Maryland, College Park, MD 20742, USA; School of Ecology, Sun Yat-sen University, Guangzhou 510275, China; College of Life Sciences, Capital Normal University, Beijing 100048, China

**Keywords:** Daohugou area, Middle Jurassic, palaeoecosystems, paleovegetation biomass, plant–insect interactions

## Abstract

Quantifying vegetation biomass is essential for understanding the structure and energy balance of terrestrial ecosystems, yet robust estimates for deep-time ecosystems remain elusive. Most palaeovegetation proxies provide qualitative or relative signals but lack a direct connection to ecosystem-scale energetics. Here, we present a probabilistic framework to reconstruct ancient vegetation biomass using fossil insect assemblages, treating insect biomass as a primary energy-based state variable. We estimated individual insect body mass from fossil morphological traits of insect fossils using relationships calibrated with modern insects, and inferred population densities based on metabolic scaling theory. These estimates were integrated to reconstruct fossil insect community biomass while explicitly accounting for sampling bias. Herbivorous insect biomass was then translated into vegetation biomass through a trophic transfer model incorporating realistic ranges of feeding rates, trophic efficiencies, and biomass accumulation ratios. All uncertainties were implemented by Monte Carlo simulations to generate full probability distributions of vegetation biomass. Sensitivity analyses reveal that insect biomass is the dominant contributor to variance in reconstructed vegetation biomass, with standardized regression coefficients substantially exceeding those of trophic transfer parameters. Ecological parameters primarily modulate vegetation biomass within constrained bounds but do not determine its order of magnitude. Comparisons with modern global vegetation datasets indicate that the reconstructed biomass corresponds to productive terrestrial ecosystems with identifiable modern biome analogues. Our results demonstrate that fossil insect assemblages encode fundamental energy-based constraints of ancient ecosystems and provide a quantitative pathway for reconstructing palaeovegetation biomass.

## INTRODUCTION

Vegetation biomass underpins the structure, functioning, and carbon storage capacity of terrestrial ecosystems [1,2]. As the primary reservoir of organic carbon and the underpinning of food webs, vegetation biomass constrains ecosystem productivity, energy flow, and biogeochemical cycling [3,4]. While modern ecosystems can be quantified using remote sensing and field inventories [5,6], providing key datasets such as global vegetation biomass [7] and vegetation type distributions [8], estimating vegetation biomass in deep time remains a fundamental challenge in palaeoecology [9,10].

Existing approaches to reconstructing palaeovegetation largely rely on plant macrofossils, palynological assemblages, or geochemical proxies [11–13]. Although these methods provide valuable insights into vegetation composition and relative abundance, they rarely yield direct estimates of standing biomass. Plant macrofossils are biased towards large and robust tissues and under-represent herbaceous vegetation [11,14], while pollen records reflect reproductive output rather than biomass and are subject to complex dispersal dynamics [15,16]. Geochemical proxies, in turn, infer vegetation indirectly through climate or productivity signals and require strong assumptions about ecosystem structure [17,18]. As a result, the energy-based scale of ancient terrestrial ecosystems—the absolute magnitude of biomass that supported food webs—has remained largely unconstrained.

Insects offer a largely untapped opportunity to bridge this gap [19,20]. As the most diverse and abundant group of terrestrial animals, insects occupy virtually all trophic levels and directly interact with vegetation through herbivory, pollination, and detritivory [21–23]. Crucially, insect communities are tightly coupled to ecosystem energy availability: insect body size, population density, and community biomass are governed by metabolic constraints and resource supply [24,25]. In modern ecosystems, insect biomass responds predictably to vegetation productivity and structure, rendering it a sensitive indicator of ecosystem energetics [25,26].

Fossil insects are abundant in many depositional settings and often preserve morphological features that can be quantitatively measured [27–30]. Body size, in particular, is routinely recoverable from fossil material and can be linked to individual body mass through well-established relationships [31]. When combined with macroecological scaling between body mass and population density, these relationships provide a pathway from fossil morphology to estimates of insect community biomass [32–34]. Despite this potential, fossil insects have traditionally been used to infer palaeoenvironmental conditions qualitatively [35,36], rather than as quantitative drivers of ecosystem-scale properties.

Most previous attempts to link insects to vegetation have emphasized trophic transfer parameters, such as feeding rates or conversion efficiencies [37–41], implicitly treating insect communities as passive intermediates. However, this perspective overlooks a key ecological principle: the scale of energy transfer through an ecosystem is fundamentally constrained by the biomass of consumer communities themselves [42,43]. Insect biomass is not merely a conduit for energy flow, but an emergent property that integrates resource availability, habitat structure, and ecological interactions [20,24,25]. If insect biomass can be robustly reconstructed from fossil assemblages, it may therefore provide a primary constraint on ancient vegetation biomass.

Here, we develop a probabilistic framework to reconstruct palaeovegetation biomass from fossil insect assemblages by explicitly treating insect biomass as a primary energy-based state variable. Our approach integrates morphological estimation of insect body mass, metabolic scaling of population density, and community-level reconstruction while accounting for fossil sampling bias. Herbivorous insect biomass is subsequently translated into vegetation biomass using a trophic transfer model that incorporates realistic ecological parameter ranges. Uncertainty is propagated throughout the workflow using Monte Carlo simulations [44,45], allowing both central estimates and variance contributions to be quantified.

We address three specific questions. First, can fossil insect morphology be used to reconstruct insect community biomass in a statistically robust and ecologically realistic manner? Second, what is the relative importance of insect biomass versus trophic transfer parameters in determining reconstructed vegetation biomass? Third, do insect-based vegetation biomass estimates correspond to modern terrestrial ecosystems with identifiable biome analogues? By answering these questions, this study reframes fossil insects from qualitative environmental indicators to quantitative drivers of fossil ecosystem energetics and provides a general framework for integrating palaeobiological data with ecosystem-scale carbon dynamics.

## RESULTS

### Fossil insect body mass enables estimation of community biomass

**Figure 1.**
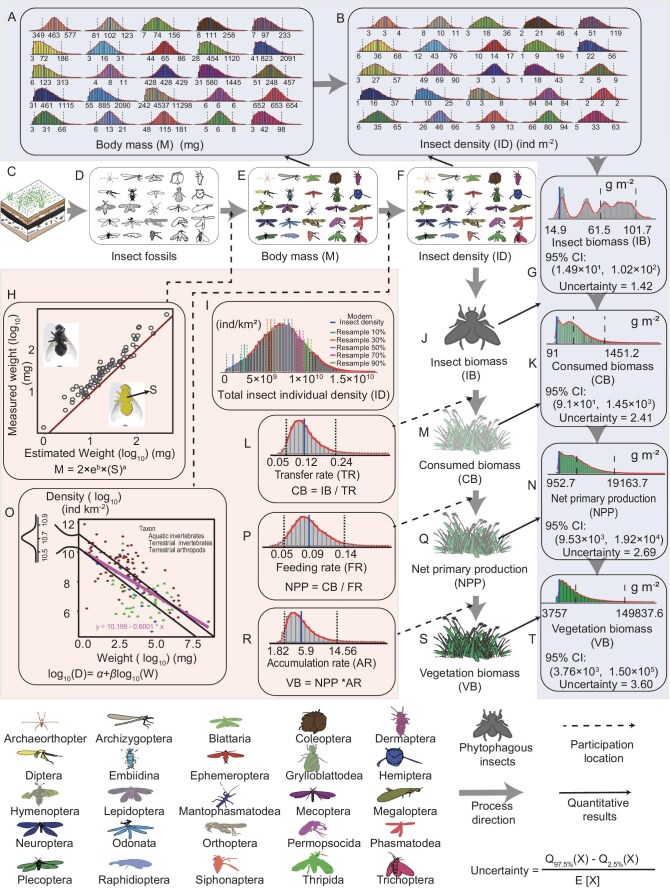
Conceptual and quantitative workflow linking insect fossil data to ecosystem-level biomass and productivity estimates. (A and B) show the empirical distributions of modern insect body mass (M, mg) and insect density (ID, ind m^−2^), respectively, compiled across major insect orders. (C and D) illustrate the sampling and taxonomic identification of insect fossils, which provide the basis for reconstructing body mass (E) and density (F) distributions. These distributions are resampled to generate uncertainty envelopes for insect individual density (I). (H) presents the relationship between estimated and measured body weight (log_10_ scale), validating the mass reconstruction approach across taxa. (O) depicts the scaling relationship between insect density and body weight for different ecological guilds (aquatic invertebrates, terrestrial invertebrates, and terrestrial arthropods). Using reconstructed insect biomass (IB), downstream trophic and energetic processes are quantified: (J) insect biomass (IB); (K) density-scaled biomass estimates; (L) transfer rate (TR; CB = IB/TR); (P) feeding rate (FR; NPP = CB/FR); and (R) accumulation rate (AR; VB = NPP × AR). These steps yield estimates of M consumed biomass (CB), (Q) net primary production (NPP), and (S and T) standing vegetation biomass (VB). (G, K, N, and T) show probability distributions (g m^−2^) of insect biomass, consumed biomass, net primary production, and vegetation biomass, respectively, with medians, 95% confidence intervals, and propagated uncertainties indicated. Taxonomic silhouettes at the bottom represent the major insect orders included in the analysis. Solid arrows indicate the process direction, dashed arrows denote participation locations, and shaded distributions reflect uncertainty propagation across resampling steps.

We compiled fossil insect records from the Yanliao Insect biota in the Daohugou area of Northeastern China. The species information in this dataset (Attachment Data) is derived from published literature, encompassing 749 insect species belonging to 417 genera, 154 families, and 25 orders, highlighting the region’s biodiversity. Among these taxa, herbivorous insects include 318 species, belonging to 183 genera, 72 families, and 16 orders. The workflow, analytical framework, and resulting estimates are summarized in Fig. 1A–T.

We reconstructed individual body mass (M) (Table 1) (Fig. 1A) for fossil phytophagous insects using measurable morphological traits, such as body and wing length, combined with relationships calibrated from modern insects (Fig. 1H). Estimated body masses spanned over three orders of magnitude, reflecting substantial inter‐taxonomic variability (Fig. 1A). Probability distributions of body mass for different taxonomic groups captured the range of plausible values, providing a quantitative basis for subsequent community‐level analyses.

**Table 1.** The list of metrics and their abbreviations.

Metric	Abbreviation	Metric	Abbreviation
M	Body mass	ID	Insect density
IB	Insect biomass	TR	Transformation rate
CB	Consumed biomass	FR	Feeding rate
NPP	Net primary production	AR	Accumulation rate
VB	Vegetation biomass		

To estimate community biomass, individual body mass was converted into potential population densities (Fig. 1B). We applied established negative power‐law relationships between log₁₀ body mass and log₁₀ population density derived from modern terrestrial and aquatic invertebrates (Fig. 1O). These relationships were not inferred from the fossil assemblages themselves but served as ecological constraints to translate body mass into estimates of individual density (Table 2). Resulting density estimates covered a biologically realistic range consistent with modern macroecological patterns, ensuring that reconstructed insect communities could be meaningfully scaled to ecosystem-level biomass.

**Table 2.** Inputs and approximate uncertainties for estimating vegetation biomass simulated by the Monte Carlo method.

	ID (ind/m^2^)	FR (100%)	AR (100%)	TR (100%)
Mean	617	0.09	5.30	0.12
2.5% tail	240	0.07	3.11	0.05
97.5% tail	1582	0.12	9.02	0.20
Uncertainty*	2.18	0.56	1.12	1.25

* Uncertainty is the ratio of (97.5% tail–2.5% tail) and mean.

Integrating body mass and inferred densities yielded estimates of total insect biomass (IB) per unit area. The median IB across fossil assemblages was 61.5 g m⁻², with a 95% confidence interval (CI) of 14.9–101.7 g m⁻² (Fig. 1G and Table 3). Uncertainty analyses indicated that variability in density estimates contributed most to the overall range, while body mass uncertainty had a secondary effect. These results demonstrate that fossil insect morphology provides a robust basis for estimating individual biomass, which, when combined with ecologically constrained density estimates, enables reconstruction of insect community biomass for ancient terrestrial ecosystems.

**Table 3.** The output values and approximate uncertainties generated during the estimation of vegetation biomass simulated by the Monte Carlo method.

	IB (g/m^2^)	CB (g/m^2^)	NPP (g/m^2^)	VB (g/m^2^)
Mean	61.5	564.3	6786	40 607
2.5% tail	14.9	91	952.7	3757
97.5% tail	101.7	1451.2	19 163.7	149 838
Uncertainty	1.42	2.41	2.69	3.6

The results show an overall right-skewed distribution. See Fig. 1 for details.

### Insect biomass dominates variance in reconstructed ecosystem energetics

**Figure 2.**
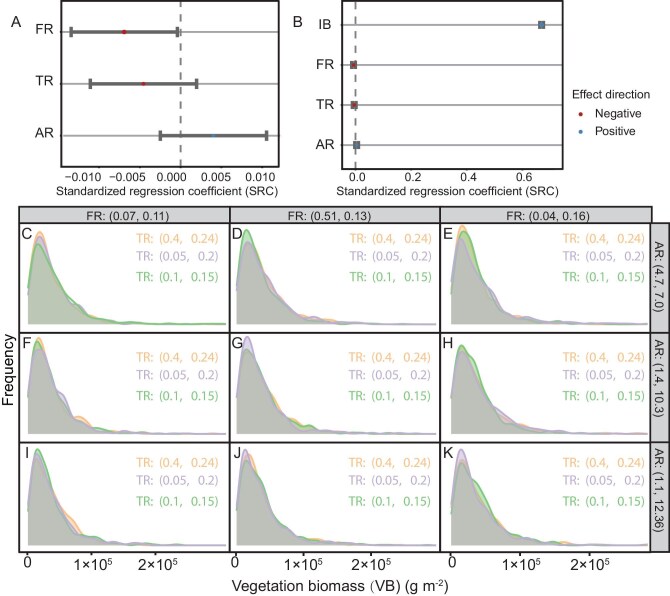
Sensitivity analysis of ecological parameters involved in reconstructing vegetation biomass. (A and B) show the standardized regression coefficients (SRC) of key drivers on vegetation biomass, covering different ecological parameters of feeding rate (FR), transform rate (TR), accumulation rate (AR) and herbivorous insect biomass (IB). Points represent estimated SRC values, error bars denote confidence intervals, and dashed vertical lines indicate zero effects; colors distinguish positive and negative effects. (C–K) display the probability density distributions of vegetation biomass (VB) across varying ranges of ecological parameters (FR, TR, AR). Parameter ranges fluctuate approximately 20% above and below their original values. Results indicate a generally right-skewed distribution of vegetation biomass, where higher TR values correlate with broader distributions and increased probabilities of high biomass. Orange, purple, and green TR colors indicate different value ranges. Consistent patterns across scenarios (F–H) and (I–K) demonstrate the model's robustness to parameter variations.

To evaluate sources of uncertainty in reconstructed vegetation biomass, we quantified the relative contributions of IB and trophic transfer parameters, including feeding rates (FR), energy transfer efficiency (TR), and vegetation accumulation rates (AR) Tables 1 and 2), using standardized regression coefficients (SRC) (Fig. 2A and B). Across all Monte Carlo simulations, IB exhibited the largest SRC values, ranging from 0.66 to 0.68, indicating a dominant influence on the variability of vegetation biomass (VB) (Fig. 1A and B).

Trophic transfer parameters showed smaller effects. SRC values for TR and FR ranged from –0.011 to –0.013, while SRC values for AR ranged from –0.002 to 0.01. These results indicate that variation in trophic transfer and accumulation parameters contributed less to overall variance in reconstructed VB relative to IB.

Propagation of uncertainty through successive trophic steps further highlighted the dominant role of IB. Mean IB uncertainty (uncertainty: 1.42) increased when converted to consumed plant biomass (CB; uncertainty: 2.41), net primary productivity (NPP; uncertainty:2.69), and final VB (uncertainty: 3.60) (Table 3), demonstrating that IB is amplified across the energy transfer pathway (Fig. 1G, K, N, and T). By contrast, variation in TR, FR, or AR produced comparatively minor changes in the distribution of VB.

These analyses demonstrate that reconstructed IB constitutes the primary source of variability in ecosystem-level energy estimates, with trophic parameters acting as secondary modulators (Fig. 2C–K).

### Phytophagous insect biomass enables quantitative estimation of vegetation biomass

Phytophagous IB was used to estimate consumed biomass (CB) by applying transformation rates (TR). Across fossil assemblages, mean CB was 1451.2 g m^−2^, with a 95% CI of 91.0–1450 g m⁻² (Fig. 1K). The uncertainty factor for CB was 2.41, reflecting the combined effects of variability in phytophagous IB and TR.

NPP was subsequently estimated by incorporating insect FR into CB calculations. The estimated range for NPP is ∼952.7–19 163.7 g m⁻², which is a left-skewed distribution, with values tending towards 953.5 g m⁻² (Fig. 1N). The associated uncertainty factor increased to 2.69, indicating amplification of variability through trophic transfer.

VB was calculated by applying AR to NPP. The estimated range for VB is ∼3.76 × 10³–1.50 × 10⁵ g m⁻², which is a left-skewed distribution, with values tending towards 3757 g m⁻² (Fig. 1T). Uncertainty increased across successive trophic steps, resulting in a final uncertainty factor of 3.60 for VB.

### Global comparison of reconstructed vegetation biomass and placement of the Daohugou ecosystem

**Figure 3.**
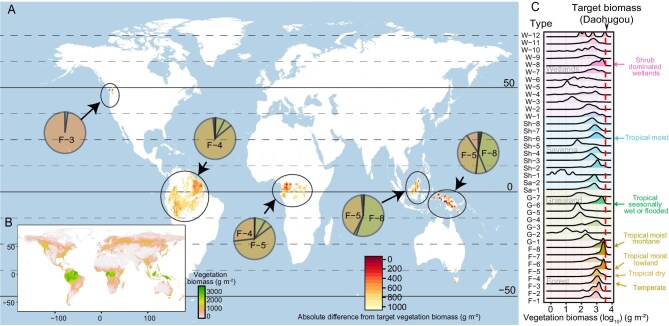
Comparison of global vegetation biomass with reconstructed Daohugou ecosystem estimates. (A) Distribution map of absolute differences between Jurassic Daohugou vegetation biomass estimates and global vegetation biomass, revealing spatial heterogeneity within and between biogeographic categories. Darker colors indicate smaller differences. The pie chart shows the proportion of each habitat type in the corresponding region. (B) Global distribution map of modern vegetation biomass, sourced from Spawn (2020) [7]. (C) Vegetation biomass distribution map across major terrestrial biome types [8], summarizing the range and concentration trends of biomass values within each biome. The reconstructed vegetation biomass for the Daohugou ecosystem is marked with a red dashed line, indicating its position relative to the distribution of modern biome-specific biomass. The abbreviations are F-1: Boreal; F-2: Subarctic; F-3: Temperate; F-4: tropical dry; F-5: tropical moist lowland; F-6: tropical mangrove vegetation; F-7: tropical swamp; F-8: tropical moist montane; G-1: Tundra; G-2: Subarctic; G-3: Subarctic; G-4: Temperate; G-5: tropical dry; G-6: tropical seasonally wet or flooded; G-7: tropical high altitude; Sa-1: Savannadry; Sa-2: Savannamoist; Sh-1: Subarctic; Sh-2: Subantarctic; Sh-3: Boreal; Sh-4: Temperate; Sh-5: tropical dry; Sh-6: tropical moist; Sh-7: tropical high altitude; Sh-8: Mediterranean-type; W-1: Permanent rivers, streams, creeks; W-2: Tundra wetlands; W-3: Alpine wetlands; W-4: Permanent inland deltas; W-5: Permanent saline, brackish, or alkaline lakes; W-6: Seasonal/intermittent saline, brackish, or alkaline lakes and flats; W-7: Seasonal/intermittent/irregular rivers/streams/creeks; W-8: Shrub-dominated wetlands; W-9: Permanent freshwater lakes; W-10: Seasonal/intermittent freshwater lakes (over 8 ha); W-11: Permanent freshwater marshes/pools (under 8 ha); and W-12: Seasonal/intermittent freshwater marshes/pools (under 8 ha). Review drawing number: GS 京(2026) 1526号.

A comparison of the Middle Jurassic Yanliao biota with modern terrestrial communities (Fig. 3A–C) provided a contrast of their estimated biomass with modern vegetation biomass across major biogeographic regions (Fig. 3). Modern vegetation biomass peaks near the equatorial belt, while lower biomass occurs in high-latitude and arid regions (Fig. 3B). The absolute difference between local vegetation biomass and specific biome benchmarks further reveals significant intra-biome variability, particularly in tropical and subtropical regions (Fig. 3A).

The reconstructed vegetational biomass of the Daohugou ecosystem falls within the upper-middle range of the biomass spectrum for major global vegetation types (Fig. 3C, red dashed line). This estimate most closely aligns with the biomass distribution characteristics of shrub wetlands and tropical moist shrublands, though it remains below the median value for closed-canopy forest biomes. Compared to grassland and savanna ecosystems, the Daohugou estimate occupies the upper range of their biomass distributions, surpassing most grassland values and overlapping with savanna biomes characterized by higher moisture and productivity. The positioning of Daohugou’s vegetation biomass within multiple biome distributions indicates substantial biomass overlap between different biome categories. The Daohugou ecosystem does not correspond to a single modern biome but occupies an intermediate position within the global vegetation biomass continuum.

## DISCUSSION

### Reconstructed vegetation biomass and its ecological implications for the Daohugou ecosystem

The reconstructed vegetation biomass for the Daohugou ecosystem provides a quantitative constraint on the energy-based structure of a Middle Jurassic terrestrial ecosystem that has previously been interpreted largely through qualitative palaeoenvironmental proxies. By deriving numerical limits on standing vegetation biomass, our results directly link fossil insect assemblages to ecosystem-wide energy availability (Fig. 1), thus offering a complementary perspective for reconstructions based on climate or taxonomic composition alone.

The magnitude of reconstructed vegetation biomass indicates an ecosystem characterized by moderate to high primary productivity. Importantly, the inferred values do not correspond to either low-biomass, resource-limited landscapes, or extremely high-biomass, closed-canopy forest ecosystems. Instead, Daohugou occupies an intermediate position within the global vegetation biomass spectrum, overlapping with productive shrubland and wetland ecosystems while remaining below typical forest biomass ranges (Fig. 3C). This placement suggests a structurally heterogeneous vegetative landscape rather than uniform vegetation cover.

This energy-based characterization is consistent with independent palaeoenvironmental evidence. Palaeoclimatic reconstructions indicate warm and humid regional conditions during deposition of the Jiulongshan Formation, which would have supported and sustained primary production [46]. Sedimentological data further indicate a lacustrine–volcaniclastic setting influenced by episodic volcanic activity [47]. Such disturbance-prone environments are typically associated with spatially heterogeneous vegetation mosaics. A vegetation biomass occurring within this reconstructed environment would be sufficient to sustain rapid post-disturbance recovery while maintaining overall ecosystem productivity, consistent with the inferred environmental dynamics.

The reconstructed vegetation biomass also provides an energy-based context for the exceptional abundance and diversity of fossil insects at Daohugou. Modern ecosystems with low standing biomass of vegetation rarely support diverse insect communities over extended time periods, whereas insect abundance and biomass scale positively with the quantity of vegetation and its structural complexity [48,49]. The Daohugou vegetation biomass estimate offers a plausible energy-based foundation for the observed insect diversity (Fig. 1).

Beyond insects, the Daohugou biota includes a diverse assemblage of vertebrates and arthropod predators [50–52]. Although these groups are not explicitly incorporated into the reconstruction, their presence imposes additional demands on the transfer of energy between trophic levels. The inferred vegetation biomass is compatible with an ecosystem capable of supporting multiple trophic levels, reinforcing the consistency of the internal energy-based reconstruction.

While vegetation biomass alone cannot resolve plant taxonomic composition, it however constrains the total amount of photosynthetic tissue present in the landscape. Biomass levels within the inferred range imply that vegetation was neither sparse nor exclusively low in stature, suggesting appreciable vertical structure. This inference is broadly consistent with reports of diverse Middle Jurassic plant assemblages from the region [51–54], without over-specification of forest type or canopy structure.

Taken together, the reconstructed vegetation biomass situates the Daohugou ecosystem within a realistic and quantitatively constrained energy-based framework (Fig. 1). By anchoring ecosystem interpretation to consumer-derived limits on energy availability, this approach strengthens palaeoecological inferences and provides a robust baseline for comparative analyses of Jurassic terrestrial ecosystems.

### A general framework for estimating vegetation biomass from fossil insect assemblages

The framework developed in this study provides a general approach for estimating vegetation biomass in deep-time terrestrial ecosystems by using a fossil insect assemblage as quantitative indicator of ecosystem energy availability. Unlike traditional palaeovegetation reconstructions [55–57] that rely primarily on plant remains or climate proxies, this framework is built around a consumer-based, energy-based constraint, explicitly treating insect biomass as a central state variable linking fossil evidence to ecosystem-scale biomass estimates (Fig. 1).

The proposed framework follows a stepwise translation from fossil morphology to ecosystem energetics (Fig. 1). Quantitative measurements of fossil insect morphology enable estimation of individual body mass using established relationships, which are then scaled to community-level insect biomass through body mass–density relationships (Fig. 1A–J). This process does not seek to reconstruct exact population sizes for individual taxa; instead, it integrates across taxonomic and demographic variability the recovery of aggregate insect biomass as a variable of the community.

A defining feature of the framework is the explicit propagation of uncertainty levels across all reconstruction steps. Uncertainty arising from morphological estimation, sampling completeness, and ecological scaling relationships is retained and quantified rather than suppressed. As a result, insect biomass, net primary production, and vegetation biomass are expressed as probability distributions rather than single point estimates (Fig. 1G–T). Such treatment allows uncertainty to be evaluated transparently and compared across ecosystems, providing the best practices in quantitative palaeoecology.

The translation from insect biomass to vegetation biomass is achieved through a trophic transfer model that links consumer biomass to primary production using ecologically realistic parameter ranges (Fig. 1L–R). Sensitivity analyses (Fig. 2) demonstrate that while feeding rates and accumulation rates influence the breadth of reconstructed vegetation biomass distributions, the overall scale of vegetation biomass is primarily constrained by the insect biomass itself (Fig. 2). This distinction highlights insect biomass as a variable that integrates multiple ecological processes, whereas trophic parameters modulate, but do not dominate, reconstruction outcomes.

The framework is robust to incomplete fossil sampling. By explicitly incorporating resampling procedures, the effects of sampling intensity on insect biomass estimates can be quantified, allowing the reconstructed vegetation biomass to be interpreted within clearly defined limits of uncertainty (Fig. 1I). This feature enables the application of the framework to fossil assemblages with varying preservational quality, rather than restricting its use to exceptionally preserved deposits.

Generality is further enhanced by the framework’s focus on community-level biomass rather than taxon-specific dynamics [29,58]. This approach reduces sensitivity to taxonomic uncertainty, which is common in fossil insect assemblages, and increases applicability across geological intervals and depositional settings. Insect-based biomass estimates also can be integrated with independent palaeoecological proxies, such as plant macrofossils or pollen [11,12], serving either as an independent energy-based constraint or as a means of cross-validation.

In summary, the proposed framework offers a flexible and transparent method for estimating vegetation biomass from fossil insect assemblages (Fig. 1). By centering on insect biomass as a primary energy-based constraint and explicitly accounting for uncertainty and sampling bias, this approach provides a parsimonious yet powerful tool for reconstructing the energy-based architecture of terrestrial ecosystems throughout deep time.

### Applicability, assumptions, and limitations of insect-based biomass reconstruction

The insect-based framework for reconstructing vegetation biomass provides a quantitative and energetically grounded approach to palaeoecological inference, but its applicability depends on a set of clearly defined assumptions and boundary conditions. Explicit articulation of these conditions is essential for evaluating the robustness of the present results and for guiding its broader application across geological contexts.

The framework is most readily applicable to terrestrial fossil assemblages that preserve insects with sufficient abundance and morphological fidelity to allow for quantitative body-size estimation. Depositional environments characterized by exceptional preservation, such as lacustrine or volcaniclastic Lagerstätten [59,60], are particularly well suited to this approach. Reduced sampling volumes primarily cause reconstructed insect biomass to trend towards lower values, resulting in vegetation biomass reconstructions that underestimate actual values.

A core assumption of the framework is that modern relationships between insect body size, population density, and biomass provide a reasonable basis for deep-time reconstruction. This assumption is supported by the physiological and metabolic constraints underlying body size–population density scaling, which are expected to be broadly conserved over evolutionary timescales. Similarly, this framework assumes that modern ranges of trophic transfer parameters define a plausible ecological envelope for ancient ecosystems. Sensitivity analyses (Fig. 2) indicate that these parameters modulate uncertainty but do not determine the overall scale of reconstructed vegetation biomass, which is primarily constrained by insect biomass itself.

The framework further assumes that fossil insect assemblages integrate ecosystem energy availability over timescales relevant to vegetation biomass. Fossil assemblages are commonly time-averaged, and reconstructed biomass should therefore be interpreted as representing long-term average conditions rather than short-term ecological states. While this limits the resolution of temporal restriction, it enhances the stability of ecosystem-scale energy-based estimates.

Several limitations are acknowledged. There are uncertainties in insect data attributable to sampling and burial biases, accuracy in estimating insect body weight, and precision in assessing insect community density. Although rigorous methodologies address these factors within our model, systematic biases cannot be fully corrected. Consequently, insect-based biomass reconstruction does not aim to provide precise estimates of vegetation biomass or detailed reconstructions of vegetation structure. Instead, such reconstructions offer probabilistic constraints on the range of biomass consistent with observed consumer communities, appropriate for ecosystem-scale energy-based analyses rather than fine-scale ecological inference. Future research should integrate independent geochemical indicators and other plant data methods to cross-validate and further calibrate this reconstruction approach.

Despite these limitations, the framework provides a transparent and conservative method for constraining ancient ecosystem energetics. When integrated with independent palaeoecological proxies, the proposed method offers a robust foundation for comparative analyses of terrestrial ecosystems through deep time, emphasizing energy-based plausibility over detailed but unconstrained reconstruction.

## MATERIALS AND METHODS

A description of all methods and materials can be found in the supplemental material.

## Supplementary Material

nwag329_Supplemental_Files

## Data Availability

All other data are available in the main text or the supplementary materials.
