# Supplementary material for: Reconstructing vegetation biomass in the Middle Jurassic Yanliao Biota from insect fossil assemblages
Source: Natl Sci Rev. 2026 Jun 2;13(12):nwag329. doi: 10.1093/nsr/nwag329 (PMC13321125; doi:10.1093/nsr/nwag329)
Supplement: nwag329_Supplemental_Files [file nwag329_supplemental_files.zip › Insect-data.docx]

Table 1 Data on species composition, body size, and habitat habits of the Daohugou insect fossil assemblage

| **Order**  **Family**  **Genus**  **Species** | **Habitat^1^** | **Feeding Class^1^** | **Total image pixel** | **Scale starting pixel** | **Scale ending pixel** | **Actual scale size/mm** | **Body pixel** | **Single wing pixel** | **b** | **b_e** | **a** | **a_e** | **r** | **Reference** |
| --- | --- | --- | --- | --- | --- | --- | --- | --- | --- | --- | --- | --- | --- | --- |
| **Archaeorthoptera**  Chresmodidae  *Jurachresmoda*  *J. gaskelli* | H | Pr | 133545 | 93 | 151 | 20 | 2013 | - | -2.14 | 0.036 | 1.331 | 0.011 | 0.94 | [1] |
| *J. sanyica* | H | Pr | 163438 | 61 | 126 | 20 | 2216 | - | -2.14 | 0.036 | 1.331 | 0.011 | 0.94 | [2] |
| **Archizygoptera**  Protomyrmeleontidae  *Protomyrmeleon*  *P. daohugouensis* | H | Pr | 147651 | 295 | 396 | 3 |  | 108162 | -2.14 | 0.036 | 1.331 | 0.011 | 0.94 | [3] |
| *P. lini* | H | Pr | 201605 | 255 | 324 | 5 | 14051 | 20397 | -2.14 | 0.036 | 1.331 | 0.011 | 0.94 | [3] |
| **Blattaria**  Fuziidae  *Arcofuzia*  *A. cana* | S | Pr |  |  |  |  |  |  | -2.14 | 0.036 | 1.331 | 0.011 | 0.94 | [4] |
| *Colorifuzia*  *C. agenora* | S | Pr | 513240 | 540 | 652 | 5 | 43406 | 36277 | -2.14 | 0.036 | 1.331 | 0.011 | 0.94 | [5] |
| *Fuzia*  *F. dadao* | S | Pr | 825565 | 70 | 81 | 1 | 9012 | 4551 | -2.14 | 0.036 | 1.331 | 0.011 | 0.94 | [6] |
| *Parvifuzia*  *P. brava* | S | Pr | 415208 | 306 | 347 | 1 | 32904 | 15701 | -2.14 | 0.036 | 1.331 | 0.011 | 0.94 | [7] |
| P. marsa | S | Pr | 310800 | 407 | 445 | 1 | 24235 | 12795 | -2.14 | 0.036 | 1.331 | 0.011 | 0.94 | [7] |
| P. peregrina | S | Pr | 306190 | 44 | 101 | 2 | 21052 | 16049 | -2.14 | 0.036 | 1.331 | 0.011 | 0.94 | [8] |
| Liberiblattinidae  *Entropia*  *E. initialis* | S | Pr | 118331 | Eosesatheris | 6941 |  | 30769 |  | -2.14 | 0.036 | 1.331 | 0.011 | 0.94 | [9] |
| Raphidiomimidae  *Divocina*  *D. noci* | S | Pr | 502980 | 70 | 89 | 1 | 10994 | 9551 | -2.14 | 0.036 | 1.331 | 0.011 | 0.94 | [10] |
| *Falcatusiblatta*  *F. gracilis* | S | Pr |  |  |  |  |  |  | -2.14 | 0.036 | 1.331 | 0.011 | 0.94 | [11] |
| *F. qiandaohua* | S | Pr |  |  |  |  |  |  | -2.14 | 0.036 | 1.331 | 0.011 | 0.94 | [11] |
| *Fortiblatta*  *F. cuspicolor* | S | Pr | 290490 | 3 | 104 | 5 | 21091 | 24050 | -2.14 | 0.036 | 1.331 | 0.011 | 0.94 | [12] |
| *Graciliblatta*  *G. bella* | S | Pr | 621732 | 234 | 377 | 5 | 44558 | 58302 | -2.14 | 0.036 | 1.331 | 0.011 | 0.94 | [13] |
| **Coleoptera**  Incertae sedis  *Cervicatinius*  *C. complanus* | S | Sa | 157728 | 61 | 134 | 2 | 53867 |  | -1.857 | 0.095 | 1.296 | 0.03 | 0.92 | [14] |
| *Juraniscus*  *J. majeri* | S | Pr&Om | 126162 | 246 | 312 | 1 | 37822 |  | -1.857 | 0.095 | 1.296 | 0.03 | 0.92 | [15] |
| *Leicarabus*  *L. parvus* | S | Pr&Om |  |  |  |  |  |  | -1.857 | 0.095 | 1.296 | 0.03 | 0.92 | [16] |
| *Serecoleus*  *S. nadbitovae* | S | Pr&Om | 295800 | 44 | 59 | 1 | 33466 |  | -1.857 | 0.095 | 1.296 | 0.03 | 0.92 | [17] |
| *Sinopeltis*  *S. amoena* | S | Pr&Om | 301490 | 22 | 60 | 1 | 29672 |  | -1.857 | 0.095 | 1.296 | 0.03 | 0.92 | [18] |
| *S. jurrasica* | S | Pr&Om | 329160 | 35 | 79 | 1 | 62770 |  | -1.857 | 0.095 | 1.296 | 0.03 | 0.92 | [18] |
| *Wuhua*  *W. jurassica* | S | Pr&Om | 405504 | 231 | 343 | 3 | 30344 |  | -1.857 | 0.095 | 1.296 | 0.03 | 0.92 | [19] |
| Alloioscarabaeidae  *Alloioscarabaeus*  *A. cheni* | S | Sa | 282552 | 234 | 257 | 1 | 28895 |  | -1.857 | 0.095 | 1.296 | 0.03 | 0.92 | [20] |
| Artematopodidae  *Sinobrevipogon*  *S. jurassicus* | S | Ph | 159636 | 96 | 153 | 2 | 11427 |  | -1.857 | 0.095 | 1.296 | 0.03 | 0.92 | [21] |
| *Tarsomegamerus*  *T. mesozoicus* | S | Ph | 237644 | 81 | 127 | 2 | 31146 |  | -1.857 | 0.095 | 1.296 | 0.03 | 0.92 | [22] |
| Asiocoleidae  *Loculitricoleus*  *L. flatus* | S | Ph |  |  |  |  |  |  | -1.857 | 0.095 | 1.296 | 0.03 | 0.92 | [23] |
| *L. tenuatus* | S | Ph |  |  |  |  |  |  | -1.857 | 0.095 | 1.296 | 0.03 | 0.92 | [23] |
| Boganiidae  *Palaeoboganium*  *P. jurassicum* | S | Ph | 197980 | 76 | 103 | 1 | 33995 |  | -1.857 | 0.095 | 1.296 | 0.03 | 0.92 | [24] |
| Buprestidae  *Sinoparathyrea*  *S. bimaculata* | S | Ph | 112942 | 518 | 570 | 2 | 76678 |  | -1.857 | 0.095 | 1.296 | 0.03 | 0.92 | [25] |
| *S. robusta* | S | Ph | 333000 | 607 | 633 | 1 | 53863 |  | -1.857 | 0.095 | 1.296 | 0.03 | 0.92 | [25] |
| *S. gracilenta* | S | Ph | 278892 | 373 | 394 | 1 | 26839 |  | -1.857 | 0.095 | 1.296 | 0.03 | 0.92 | [25] |
| Cerophytidae  *Jurassophytum*  *J. cleidecostae* | E&S | Ph | 150150 | 206 | 243 | 2 | 7446 |  | -1.857 | 0.095 | 1.296 | 0.03 | 0.92 | [26] |
| Cleridae  *Protoclerus*  *P. korynetoides* | S | Pr | 300846 | 38 | 62 | 1 | 8060 |  | -1.857 | 0.095 | 1.296 | 0.03 | 0.92 | [27] |
| *Wangweiella*  *W. calloviana* | S | Pr | 293181 | 61 | 89 | 1 | 10685 |  | -1.857 | 0.095 | 1.296 | 0.03 | 0.92 | [27] |
| Coptoclavidae  *Daohugounectes*  *D. primitivus* | H | Pr | 249738 | 520 | 627 | 2 | 61953 | - | -1.857 | 0.095 | 1.296 | 0.03 | 0.92 | [28] |
| *Cupedidae*  *Gracilicupes*  *G. crassicruralis* | S | Sa | 335790 | 167 | 208 | 2 | 19597 |  | -1.857 | 0.095 | 1.296 | 0.03 | 0.92 | [29] |
| *G. tenuicruralis* | S | Sa | 250631 | 111 | 166 | 2 | 27071 |  | -1.857 | 0.095 | 1.296 | 0.03 | 0.92 | [29] |
| *Latocupes*  *L. angustilabialis* | S | Sa |  |  |  |  |  |  | -1.857 | 0.095 | 1.296 | 0.03 | 0.92 | [30] |
| *L. collaris* | S | Sa |  |  |  |  |  |  | -1.857 | 0.095 | 1.296 | 0.03 | 0.92 | [30] |
| *L. latilabialis* | S | Sa | 165710 | 60 | 100 | 2 | 18801 |  | -1.857 | 0.095 | 1.296 | 0.03 | 0.92 | [30] |
| *Mesocupes*  *M. angustilabialis* | S | Sa | 176993 | 74 | 109 | 2 | 20037 |  | -1.857 | 0.095 | 1.296 | 0.03 | 0.92 | [30] |
| *M. collaris* | S | Sa | 171054 | 140 | 172 | 2 | 24844 |  | -1.857 | 0.095 | 1.296 | 0.03 | 0.92 | [30] |
| *M. latilabialis* | S | Sa |  |  |  |  |  |  | -1.857 | 0.095 | 1.296 | 0.03 | 0.92 | [30] |
| *Dermestidae*  *Paradermestes*  *P. jurassicus* | S | Om |  |  |  |  |  |  | -1.857 | 0.095 | 1.296 | 0.03 | 0.92 | [31] |
| *Derodontidae*  *Juropeltastica*  *J. sinica* | S | Pr |  |  |  |  |  |  | -1.857 | 0.095 | 1.296 | 0.03 | 0.92 | [32] |
| Elateridae  *Clavelater*  *C. ningchengensis* | E&S | Ph | 197334 | 328 | 404 | 1 | 55122 |  | -2.836 | 0.32 | 1.56 | 0.093 | 0.95 | [33] |
| *Paradesmatus*  *P. baiae* | E&S | Ph | 144648 | 135 | 162 | 1 | 42501 |  | -2.836 | 0.32 | 1.56 | 0.093 | 0.95 | [34] |
| *P. ponomarenkoi* | E&S | Ph | 147496 | 81 | 111 | 1 | 20374 |  | -2.836 | 0.32 | 1.56 | 0.093 | 0.95 | [34] |
| *Paraprotagrypnus*  *P. superbus* | E&S | Ph | 335508 | 158 | 201 | 1 | 35036 |  | -2.836 | 0.32 | 1.56 | 0.093 | 0.95 | [35] |
| *Protagrypnus*  *P. robustus* | E&S | Ph | 158497 | 71 | 92 | 1 | 25119 |  | -2.836 | 0.32 | 1.56 | 0.093 | 0.95 | [34] |
| Jurodidae  *Jurodes*  *J. daohugouensis* | S | Om | 418309 | 185 | 253 | 1 | 59651 |  | -1.857 | 0.095 | 1.296 | 0.03 | 0.92 | [36] |
| *J. pygmaeus* | S | Om | 212704 | 97 | 146 | 1 | 7706 |  | -1.857 | 0.095 | 1.296 | 0.03 | 0.92 | [36] |
| Lasiosynidae  *Anacapitis*  *A. plata* | S | Ph |  |  |  |  |  |  | -1.857 | 0.095 | 1.296 | 0.03 | 0.92 | [23] |
| *Bupredactyla*  *B. magna* | S | Ph | 59832 | 20 | 43 | 2 | 17798 |  | -1.857 | 0.095 | 1.296 | 0.03 | 0.92 | [37] |
| *Lasiosyne*  *L. daohugouensis* | S | Ph | 164616 | 302 | 334 | 2 | 23540 |  | -1.857 | 0.095 | 1.296 | 0.03 | 0.92 | [37] |
| *L. euglyphea* | S | Ph | 291031 | 292 | 355 | 3 | 63653 |  | -1.857 | 0.095 | 1.296 | 0.03 | 0.92 | [38] |
| *L. fedorenkoi* | S | Ph | 190530 | 12 | 42 | 2 | 16453 |  | -1.857 | 0.095 | 1.296 | 0.03 | 0.92 | [37] |
| *L. gratiosa* | S | Ph | 329427 | 36 | 65 | 2 | 27259 |  | -1.857 | 0.095 | 1.296 | 0.03 | 0.92 | [37] |
| *L. laxa* | S | Ph |  |  |  |  |  |  | -1.857 | 0.095 | 1.296 | 0.03 | 0.92 | [39] |
| *L. quadricollis* | S | Ph | 178112 | 307 | 337 | 2 | 27059 |  | -1.857 | 0.095 | 1.296 | 0.03 | 0.92 | [37] |
| *Parelateriformius*  *P. capitifossus* | S | Ph |  |  |  |  |  |  | -1.857 | 0.095 | 1.296 | 0.03 | 0.92 | [40] |
| *P. communis* | S | Ph |  |  |  |  |  |  | -1.857 | 0.095 | 1.296 | 0.03 | 0.92 | [40] |
| *P. mirabdominis* | S | Ph |  |  |  |  |  |  | -1.857 | 0.095 | 1.296 | 0.03 | 0.92 | [40] |
| *P. villosus* | S | Ph |  |  |  |  |  |  | -1.857 | 0.095 | 1.296 | 0.03 | 0.92 | [40] |
| Lucanidae  *Juraesalus*  *J. atavus* | S | Sa | 334422 | 172 | 215 | 2 | 15471 |  | -1.857 | 0.095 | 1.296 | 0.03 | 0.92 | [41] |
| Melyridae  *Sinomelyris*  *S. praedecessor* | S | Sa | 165326 | 258 | 323 | 1 | 53189 |  | -1.857 | 0.095 | 1.296 | 0.03 | 0.92 | [15] |
| Monotomidae  *Jurorhizophagus*  *J. alienus* | E | Ph |  |  |  |  |  |  | -1.857 | 0.095 | 1.296 | 0.03 | 0.92 | [42] |
| Ochodaeidae  *Mesochodaeus*  *M. daohugouensis* | S | Sa |  |  |  |  |  |  | -1.857 | 0.095 | 1.296 | 0.03 | 0.92 | [43] |
| Ommatidae  *Brochocoleus*  *B. applanatus* | E | Ph |  |  |  |  |  |  | -1.857 | 0.095 | 1.296 | 0.03 | 0.92 | [23] |
| *B. magnus* | E | Ph |  |  |  |  |  |  | -1.857 | 0.095 | 1.296 | 0.03 | 0.92 | [23] |
| *B. validus* | E | Ph |  |  |  |  |  |  | -1.857 | 0.095 | 1.296 | 0.03 | 0.92 | [23] |
| *Notocupes*  *N. (Notocupes) pingi* | E | Ph |  |  |  |  |  |  | -1.857 | 0.095 | 1.296 | 0.03 | 0.92 | [44] |
| *Omma*  *O. delicate* | E | Ph | 569985 | 59 | 169 | 2 | 58974 |  | -1.857 | 0.095 | 1.296 | 0.03 | 0.92 | [45] |
| *Pareuryomma*  *P. ancistrodonta* | E | Ph | 810726 | 85 | 292 | 2 | 115921 |  | -1.857 | 0.095 | 1.296 | 0.03 | 0.92 | [45] |
| *Tetraphalerus*  *T. decorosus* | E | Ph | 811752 | 101 | 228 | 1 | 104180 |  | -1.857 | 0.095 | 1.296 | 0.03 | 0.92 | [45] |
| Parandrexidae  *Parandrexis*  *P. agilis* | S | Ph |  |  |  |  |  |  | -1.857 | 0.095 | 1.296 | 0.03 | 0.92 | [46] |
| *P. longicornis* | S | Ph |  |  |  |  |  |  | -1.857 | 0.095 | 1.296 | 0.03 | 0.92 | [46] |
| *P. oblongis* | S | Ph |  |  |  |  |  |  | -1.857 | 0.095 | 1.296 | 0.03 | 0.92 | [46] |
| Prionoceridae  *Idgiaites*  *I. jurassicus* | S | Ph&Sa | 471255 | 395 | 429 | 1 | 62664 |  | -1.857 | 0.095 | 1.296 | 0.03 | 0.92 | [47] |
| Ripiphoridae  *Archaeoripiphorus*  *A. nuwa* | S | Om | 270674 | 279 | 445 | 5 | 81930 |  | -1.857 | 0.095 | 1.296 | 0.03 | 0.92 | [48] |
| Schizophoridae  *Abrohadeocoleodes*  *A. eurycladus* | H | Ph&Sa | 251625 | 55 | 128 | 2 | 26338 | 10395 | -1.857 | 0.095 | 1.296 | 0.03 | 0.92 | [49] |
| *A. nii* | H | Ph&Sa | 284082 | 143 | 212 | 2 | 26886 | 8915 | -1.857 | 0.095 | 1.296 | 0.03 | 0.92 | [49] |
| *A. ooideus* | H | Ph&Sa | 330372 | 120 | 177 | 2 | 32906 | 11224 | -1.857 | 0.095 | 1.296 | 0.03 | 0.92 | [49] |
| *A. patefactus* | H | Ph&Sa | 83385 | 57 | 97 | 2 | 11227 | 5937 | -1.857 | 0.095 | 1.296 | 0.03 | 0.92 | [49] |
| *Homocatabrycus*  *H. liui* | H | Pr | 98268 | 314 | 349 | 2 | 49429 | 18385 | -1.857 | 0.095 | 1.296 | 0.03 | 0.92 | [38] |
| *Menopraesagus*  *M. explanatus* | H | Ph&Sa | 192136 | 163 | 238 | 2 | 57487 | - | -1.857 | 0.095 | 1.296 | 0.03 | 0.92 | [38] |
| *M. grammicus* | H | Ph&Sa | 131705 | 99 | 147 | 2 |  | 13922 | -1.857 | 0.095 | 1.296 | 0.03 | 0.92 | [38] |
| *M. oryziformis* | H | Ph&Sa | 202124 | 447 | 500 | 2 | 21843 | 11372 | -1.857 | 0.095 | 1.296 | 0.03 | 0.92 | [49] |
| *M. oxycerus* | H | Ph&Sa | 127008 | 23 | 97 | 2 | 62832 | 27761 | -1.857 | 0.095 | 1.296 | 0.03 | 0.92 | [38] |
| *Sinoschizala*  *S. darani* | H | Ph&Sa | 150696 | 260 | 276 | 1 |  | 11744 | -1.857 | 0.095 | 1.296 | 0.03 | 0.92 | [50] |
| Staphylinidae  *Juroglypholoma*  *J. antiquum* | S | Pr&Sa |  |  |  |  |  |  | -1.857 | 0.095 | 1.296 | 0.03 | 0.92 | [51] |
| *Mesapatetica*  *M. aenigmatica* | S | Pr&Sa |  |  |  |  |  |  | -1.857 | 0.095 | 1.296 | 0.03 | 0.92 | [52] |
| *Protolisthaerus*  *P. jurassicus* | S | Pr&Sa | 276816 | 260 | 304 | 2 | 11451 |  | -1.857 | 0.095 | 1.296 | 0.03 | 0.92 | [53] |
| *Sinanthobium*  *S. daohugouense* | S | Pr&Sa |  |  |  |  |  |  | -1.857 | 0.095 | 1.296 | 0.03 | 0.92 | [54] |
| Trachypachidae  *Eodromeus*  *E. daohugouensis* | E&S | Pr&Sa | 328155 | 494 | 569 | 2 | 55584 |  | -1.857 | 0.095 | 1.296 | 0.03 | 0.92 | [55] |
| *E. robustus* | E&S | Pr&Sa | 239520 | 354 | 397 | 2 | 38122 |  | -1.857 | 0.095 | 1.296 | 0.03 | 0.92 | [55] |
| *Unda*  *U. chifengensis* | E&S | Pr&Sa | 215670 | 324 | 394 | 2 | 47057 |  | -1.857 | 0.095 | 1.296 | 0.03 | 0.92 | [55] |
| Trogossitidae  *Latitergum*  *L. glabrum* | S | Pr&Om | 380075 | 22 | 41 | 1 | 24905 |  | -1.857 | 0.095 | 1.296 | 0.03 | 0.92 | [56] |
| *Marginulatus*  *M. venustus* | S | Pr&Om | 358272 | 31 | 68 | 1 | 43459 |  | -1.857 | 0.095 | 1.296 | 0.03 | 0.92 | [56] |
| **Dermaptera**  Incertae sedis  *Atopderma*  *A. ellipta* | S | Pr |  |  |  |  |  |  | -2.14 | 0.036 | 1.331 | 0.011 | 0.94 | [57] |
| Bellodermatidae  *Belloderma*  *B. arcuata* | S | Om | 263929 | 130 | 276 | 2 | 18987 |  | -2.14 | 0.036 | 1.331 | 0.011 | 0.94 | [58] |
| *B. ovata* | S | Om | 239250 | 187 | 388 | 2 | 22653 |  | -2.14 | 0.036 | 1.331 | 0.011 | 0.94 | [58] |
| Dermapteridae  *Jurassimedeola*  *J. orientalis* | S | Om |  |  |  |  |  |  | -2.14 | 0.036 | 1.331 | 0.011 | 0.94 | [59] |
| *Palaeodermapteron*  *P. dicranum* | S | Om | 148969 | 35 | 73 | 4 | 9262 |  | -2.14 | 0.036 | 1.331 | 0.011 | 0.94 | [60] |
| *Sinopalaeodermata*  *S. neimonggolensis* | S | Om | 171798 | 319 | 361 | 3 | 14029 |  | -2.14 | 0.036 | 1.331 | 0.011 | 0.94 | [59] |
| Protodiplatyidae  *Abrderma*  *A. gracilentum* | S | Ph&Sa |  |  |  |  |  |  | -2.14 | 0.036 | 1.331 | 0.011 | 0.94 | [61] |
| *Perissoderma*  *P. triangulum* | S | Ph&Sa |  |  |  |  |  |  | -2.14 | 0.036 | 1.331 | 0.011 | 0.94 | [61] |
| **Diptera**  Anisopodidae  *Megarhyphus*  *M. rarus* | S | Ph&Sa | 60993 | 129 | 145 | 1 | 13396 |  | -2.197 | 0.089 | 1.309 | 0.03 | 0.94 | [62] |
| Archisargidae  *Archirhagio*  *A. gracilentus* | E&S | Ph&Sa | 756208 | 488 | 604 | 3 | 35844 |  | -2.197 | 0.089 | 1.309 | 0.03 | 0.94 | [63] |
| *A. striatus* | E&S | Ph&Sa |  |  |  |  |  |  | -2.197 | 0.089 | 1.309 | 0.03 | 0.94 | [64] |
| *A. varius* | E&S | Ph&Sa | 350796 | 369 | 399 | 1 | 24925 | 18460 | -2.197 | 0.089 | 1.309 | 0.03 | 0.94 | [64] |
| *A. zhangi* | E&S | Ph&Sa | 609696 | 409 | 435 | 1 | 32351 | 15056 | -2.197 | 0.089 | 1.309 | 0.03 | 0.94 | [64] |
| *Archisargus*  *A. spurivenius* | E&S | Ph&Sa | 541780 | 15 | 63 | 2 | 24753 |  | -2.197 | 0.089 | 1.309 | 0.03 | 0.94 | [65] |
| *A. strigatus* | E&S | Ph&Sa | 158224 | 66 | 130 | 2 | 91923 | 52237 | -2.197 | 0.089 | 1.309 | 0.03 | 0.94 | [65] |
| *Calosargus*  *C. (Calosargus) antiquus* | E&S | Ph&Sa | 343185 | 383 | 424 | 1 | 50129 | 15672 | -2.197 | 0.089 | 1.309 | 0.03 | 0.94 | [66] |
| *C. (Calosargus) bellus* | E&S | Ph&Sa | 391300 | 19 | 81 | 2 | 35979 | 15320 | -2.197 | 0.089 | 1.309 | 0.03 | 0.94 | [66] |
| *C. (Calosargus) daohugouensis* | E&S | Ph&Sa | 222360 | 549 | 598 | 1 |  | 27446 | -2.197 | 0.089 | 1.309 | 0.03 | 0.94 | [66] |
| *C. (Calosargus) hani* | E&S | Ph&Sa | 270396 | 22 | 56 | 1 | 48704 | 14592 | -2.197 | 0.089 | 1.309 | 0.03 | 0.94 | [66] |
| *C. (Calosargus) tenuicellulatus* | E&S | Ph&Sa | 358940 | 493 | 598 | 1 | 49514 |  | -2.197 | 0.089 | 1.309 | 0.03 | 0.94 | [66] |
| *C. (Calosargus) validus* | E&S | Ph&Sa | 377848 | 77 | 110 | 1 | 25582 |  | -2.197 | 0.089 | 1.309 | 0.03 | 0.94 | [66] |
| *C. (Pterosargus) sinicus* | E&S | Ph&Sa | 126630 | 202 | 221 | 1 | 113432 |  | -2.197 | 0.089 | 1.309 | 0.03 | 0.94 | [67] |
| *Daohugosargus*  *D. eximius* | E&S | Ph&Sa | 445875 | 130 | 175 | 1 | 102737 |  | -2.197 | 0.089 | 1.309 | 0.03 | 0.94 | [68] |
| *Flagellisargus*  *F. robustus* | E&S | Ph&Sa | 520260 | 434 | 508 | 1 | 140754 |  | -2.197 | 0.089 | 1.309 | 0.03 | 0.94 | [69] |
| *F. sinicus* | E&S | Ph&Sa | 475710 | 294 | 338 | 1 |  | 38633 | -2.197 | 0.089 | 1.309 | 0.03 | 0.94 | [69] |
| *F. venustus* | E&S | Ph&Sa | 388652 | 74 | 183 | 1 |  | 109100 | -2.197 | 0.089 | 1.309 | 0.03 | 0.94 | [69] |
| *Mesosolva*  *M. daohugouensis* | E&S | Ph&Sa |  |  |  |  |  |  | -2.197 | 0.089 | 1.309 | 0.03 | 0.94 | [70] |
| *M. sinensis* | E&S | Ph&Sa | 3887114 | 554 | 598 | 2 | 14635 | 18746 | -2.197 | 0.089 | 1.309 | 0.03 | 0.94 | [71] |
| *M. zhangae* | E&S | Ph&Sa | 78996 | 335 | 387 | 1 |  | 36733 | -2.197 | 0.089 | 1.309 | 0.03 | 0.94 | [71] |
| *Novisargus*  *N. rarus* | E&S | Ph&Sa | 504296 | 220 | 267 | 1 | 42097 | 25901 | -2.197 | 0.089 | 1.309 | 0.03 | 0.94 | [72] |
| *Ovisargus*  *O. (Ovisargus) singulus* | E&S | Ph&Sa | 458586 | 329 | 377 | 1 | 32574 | 11688 | -2.197 | 0.089 | 1.309 | 0.03 | 0.94 | [72] |
| *Sharasargus*  *S. fortis* | E&S | Ph&Sa | 389400 | 120 | 159 | 1 | 44128 | 34462 | -2.197 | 0.089 | 1.309 | 0.03 | 0.94 | [68] |
| *S. maculus* | E&S | Ph&Sa | 131328 | 412 | 445 | 1 | 41697 | 19589 | -2.197 | 0.089 | 1.309 | 0.03 | 0.94 | [72] |
| *Tabanisargus*  *T. daohugouensis* | E&S | Ph&Sa | 286638 | 133 | 150 | 1 | 69898 |  | -2.197 | 0.089 | 1.309 | 0.03 | 0.94 | [72] |
| *Uranorhagio*  *U. asymmetricus* | E&S | Ph&Sa | 349695 | 460 | 494 | 2 | 11596 | 5423 | -2.197 | 0.089 | 1.309 | 0.03 | 0.94 | [73] |
| *U. daohugouensis* | E&S | Ph&Sa | 328704 | 471 | 496 | 2 | 18242 | 8926 | -2.197 | 0.089 | 1.309 | 0.03 | 0.94 | [74] |
| *U. deviatus* | E&S | Ph&Sa | 322762 | 472 | 488 | 1 | 10761 | 6168 | -2.197 | 0.089 | 1.309 | 0.03 | 0.94 | [74] |
| *Athericidae*  *Qiyia*  *Q. jurassica* | E&S | Pr | 360000 | 371 | 454 | 5 | 19919 |  | -2.197 | 0.089 | 1.309 | 0.03 | 0.94 | [75] |
| Axymyiidae  *Juraxymyia*  *J. fossilis* | E&S | Sa | 324836 | 245 | 292 | 1 | 23791 | 24585 | -2.197 | 0.089 | 1.309 | 0.03 | 0.94 | [76] |
| *Parapsocus*  *P. pectinatus* | E&S | Sa |  |  |  |  |  |  | -2.197 | 0.089 | 1.309 | 0.03 | 0.94 | [77] |
| *Raraxymyia*  *R. parallela* | E&S | Sa | 81432 | 376 | 303 | 1 | 14296 | 12446 | -2.197 | 0.089 | 1.309 | 0.03 | 0.94 | [78] |
| *R. proxima* | E&S | Sa | 87100 | 278 | 314 | 1 | 13319 | 12576 | -2.197 | 0.089 | 1.309 | 0.03 | 0.94 | [78] |
| *Sinaxymyia*  *S. rara* | E&S | Sa | 484329 | 335 | 380 | 1 | 8983 | 8612 | -2.197 | 0.089 | 1.309 | 0.03 | 0.94 | [76] |
| Blephariceridae  *Blephadejura*  *B. propria* | H&S | Sa |  |  |  |  |  |  | -2.197 | 0.089 | 1.309 | 0.03 | 0.94 | [79] |
| *Brianina*  *B. longitibialis* | H&S | Sa | 223584 | 137 | 172 | 1 | 9219 | 13614 | -2.197 | 0.089 | 1.309 | 0.03 | 0.94 | [80] |
| *Megathon*  *M. brodskyi* | H&S | Sa | 251245 | 138 | 174 | 1 | 20205 | 15702 | -2.197 | 0.089 | 1.309 | 0.03 | 0.94 | [80] |
| Hennigmatidae  *Daohennigma*  *D. panops* | E&S | Ph&Sa |  |  |  |  |  |  | -2.197 | 0.089 | 1.309 | 0.03 | 0.94 | [79] |
| Kovalevisargidae  *Kerosargus*  *K. sororius* | E&S | Ph&Sa | 419175 | 398 | 445 | 1 | 93576 | 35552 | -2.197 | 0.089 | 1.309 | 0.03 | 0.94 | [81] |
| *Kovalevisargus*  *K. brachypterus* | E&S | Ph&Sa | 463968 | 236 | 283 | 1 | 32683 | 18913 | -2.197 | 0.089 | 1.309 | 0.03 | 0.94 | [81] |
| *K. macropterus* | E&S | Ph&Sa | 468078 | 380 | 424 | 1 | 43311 | 21406 | -2.197 | 0.089 | 1.309 | 0.03 | 0.94 | [81] |
| Limoniidae  *Architipula*  *A. chinensis* | H&S | Sa | 80040 | 129 | 157 | 1 | 2217 | 2115 | -2.197 | 0.089 | 1.309 | 0.03 | 0.94 | [82] |
| *A. conformis* | H&S | Sa | 73402 | 115 | 179 | 8 | 3169 | 4043 | -2.197 | 0.089 | 1.309 | 0.03 | 0.94 | [83] |
| *A. insolita* | H&S | Sa | 50629 | 108 | 141 | 1 | 3879 | 4888 | -2.197 | 0.089 | 1.309 | 0.03 | 0.94 | [84] |
| *A. trichoclada* | H&S | Sa | 39026 | 66 | 88 | 1 | 1788 | 2261 | -2.197 | 0.089 | 1.309 | 0.03 | 0.94 | [84] |
| *Eotipulina*  *E. eximia* | H&S | Sa | 46540 | 43 | 77 | 1 | 5530 | 5381 | -2.197 | 0.089 | 1.309 | 0.03 | 0.94 | [84] |
| *Eotipuloptera*  *E. dignata* | H&S | Sa | 77748 | 323 | 335 | 1 | 5314 | 5576 | -2.197 | 0.089 | 1.309 | 0.03 | 0.94 | [84] |
| *Cretolimonia*  *C. excelsa* | H&S | Sa | 90025 | 240 | 255 | 1 | 5859 | 5101 | -2.197 | 0.089 | 1.309 | 0.03 | 0.94 | [85] |
| *Mesotipula*  *M. gloriosa* | H&S | Sa | 76112 | 240 | 256 | 1 | 5677 | 7198 | -2.197 | 0.089 | 1.309 | 0.03 | 0.94 | [85] |
| Mesosciophilidae  *Jurasciophila*  *J. curvula* | S | Ph&Sa | 265200 | 31 | 63 | 1 | 10524 | 7676 | -2.197 | 0.089 | 1.309 | 0.03 | 0.94 | [86] |
| *J. lepida* | S | Ph&Sa | 212898 | 31 | 62 | 1 | 10246 | 7790 | -2.197 | 0.089 | 1.309 | 0.03 | 0.94 | [86] |
| *Mesosciophila*  *M. abstracta* | S | Ph&Sa | 89301 | 191 | 223 | 1 | 8175 | 6632 | -2.197 | 0.089 | 1.309 | 0.03 | 0.94 | [87] |
| *M. antiqua* | S | Ph&Sa | 263252 | 162 | 274 | 3 | 18700 | 7975 | -2.197 | 0.089 | 1.309 | 0.03 | 0.94 | [88] |
| *M. eucalla* | S | Ph&Sa | 246333 | 49 | 70 | 1 | 6010 | 4587 | -2.197 | 0.089 | 1.309 | 0.03 | 0.94 | [89] |
| *M. sigmoidea* | S | Ph&Sa | 305509 | 17 | 53 | 1 | 17207 | 9726 | -2.197 | 0.089 | 1.309 | 0.03 | 0.94 | [90] |
| *Mesosciophilodes*  *M. synchrona* | S | Ph&Sa | 75088 | 141 | 161 | 1 | 6302 | 3099 | -2.197 | 0.089 | 1.309 | 0.03 | 0.94 | [87] |
| *Paramesosciophilodes*  *P. aequus* | S | Ph&Sa | 373160 | 149 | 199 | 1 | 24051 | 11395 | -2.197 | 0.089 | 1.309 | 0.03 | 0.94 | [90] |
| *P. bellus* | S | Ph&Sa | 359613 | 425 | 465 | 1 | 23633 | 12149 | -2.197 | 0.089 | 1.309 | 0.03 | 0.94 | [91] |
| *P. eximia* | S | Ph&Sa | 84510 | 112 | 142 | 1 | 5280 | 4328 | -2.197 | 0.089 | 1.309 | 0.03 | 0.94 | [87] |
| *P. ningchengensis* | S | Ph&Sa | 72324 | 93 | 121 | 1 | 6442 | 4053 | -2.197 | 0.089 | 1.309 | 0.03 | 0.94 | [89] |
| *P. rarissima* | S | Ph&Sa | 342240 | 423 | 470 | 1 | 22738 | 17740 | -2.197 | 0.089 | 1.309 | 0.03 | 0.94 | [91] |
| *Similsciophila*  *S. singularis* | S | Ph&Sa | 380699 | 210 | 235 | 1 | 7034 | 4203 | -2.197 | 0.089 | 1.309 | 0.03 | 0.94 | [92] |
| *S. sinuata* | S | Ph&Sa | 380160 | 225 | 250 | 1 | 5015 | 4681 | -2.197 | 0.089 | 1.309 | 0.03 | 0.94 | [92] |
| Nemestrinidae  *Ahirmoneura*  *A. neimengguensis* | S | Pa&Ph | 273229 | 517 | 547 | 2 | 24626 |  | -2.197 | 0.089 | 1.309 | 0.03 | 0.94 | [93] |
| *Orientisargidae*  *Orientisargus*  *O. illecebrosus* | S | Ph | 160875 | 197 | 229 | 1 | 23607 | 10397 | -2.197 | 0.089 | 1.309 | 0.03 | 0.94 | [73] |
| Pediciidae  *Praearchitipula*  *P. abnormis* | E&S | Ph | 509030 | 253 | 266 | 1 | 2523 | 7563 | -2.197 | 0.089 | 1.309 | 0.03 | 0.94 | [83] |
| *P. apprima* | E&S | Ph | 391140 | 535 | 559 | 1 | 18029 | 15035 | -2.197 | 0.089 | 1.309 | 0.03 | 0.94 | [94] |
| *P. mirabilis* | E&S | Ph | 607695 | 517 | 553 | 1 | 16474 | 15652 | -2.197 | 0.089 | 1.309 | 0.03 | 0.94 | [94] |
| Perissommatidae  *Perissordes*  *P. pilosus* | S | Ph |  |  |  |  |  |  | -2.197 | 0.089 | 1.309 | 0.03 | 0.94 | [79] |
| Protobrachyceridae  *Protobrachyceron*  *P. sinensis* | S | Ph |  |  |  |  |  |  | -2.197 | 0.089 | 1.309 | 0.03 | 0.94 | [95] |
| Protopleciidae  *Epimesoplecia*  *E. ambloneura* | E&S | Ph | 481182 | 274 | 289 | 1 | 5299 | 4500 | -2.197 | 0.089 | 1.309 | 0.03 | 0.94 | [96] |
| *E. elenae* | E&S | Ph | 73580 | 164 | 180 | 1 | 9378 | 6459 | -2.197 | 0.089 | 1.309 | 0.03 | 0.94 | [97] |
| *E. macrostrena* | E&S | Ph | 505182 | 498 | 519 | 1 | 7921 | 5387 | -2.197 | 0.089 | 1.309 | 0.03 | 0.94 | [96] |
| *E. plethora* | E&S | Ph | 487430 | 524 | 544 | 1 | 8744 |  | -2.197 | 0.089 | 1.309 | 0.03 | 0.94 | [96] |
| *E. prosoneura* | E&S | Ph | 420000 | 309 | 326 | 1 | 3001 | 5293 | -2.197 | 0.089 | 1.309 | 0.03 | 0.94 | [96] |
| *E. shcherbakovi* | E&S | Ph | 79590 | 171 | 197 | 1 | 13364 | 11213 | -2.197 | 0.089 | 1.309 | 0.03 | 0.94 | [97] |
| *E. stana* | E&S | Ph | 534287 | 258 | 275 | 1 | 5000 | 5737 | -2.197 | 0.089 | 1.309 | 0.03 | 0.94 | [96] |
| *Mesoplecia*  *M. anfracta* | E&S | Ph | 235074 | 89 | 237 | 4 | 29560 | 21989 | -2.197 | 0.089 | 1.309 | 0.03 | 0.94 | [88] |
| *M. coadnata* | E&S | Ph | 242963 | 81 | 220 | 3 | 28166 | 22623 | -2.197 | 0.089 | 1.309 | 0.03 | 0.94 | [88] |
| *M. fastigata* | E&S | Ph | 400176 | 297 | 322 | 1 | 7518 | 8259 | -2.197 | 0.089 | 1.309 | 0.03 | 0.94 | [98] |
| *M. mediana* | E&S | Ph | 270928 | 334 | 370 | 1 | 16111 | 10147 | -2.197 | 0.089 | 1.309 | 0.03 | 0.94 | [97] |
| *M. plena* | E&S | Ph | 451440 | 494 | 532 | 1 | 20178 | 17455 | -2.197 | 0.089 | 1.309 | 0.03 | 0.94 | [98] |
| *M. sinica* | E&S | Ph | 107536 | 33 | 77 | 1 |  | 32501 | -2.197 | 0.089 | 1.309 | 0.03 | 0.94 | [97] |
| Protorhyphidae  *Protorhyphus*  *P. neimonggolensis* | E&S | Ph | 108438 | 110 | 141 | 1 | 8664 | 5515 | -2.197 | 0.089 | 1.309 | 0.03 | 0.94 | [62] |
| Ptychopteridae  *Crenoptychoptera*  *C. decorosa* | H&S | Sa | 332044 | 275 | 424 | 3 | 43313 | 73155 | -2.197 | 0.089 | 1.309 | 0.03 | 0.94 | [99] |
| *Eoptychoptera*  *E. ansorgei* | H&S | Sa | 73656 | Eosesatheris | 2257 |  | 3512 | 4029 | -2.197 | 0.089 | 1.309 | 0.03 | 0.94 | [100] |
| *E. jurassica* | H&S | Sa | 88245 | Eosesatheris | 861 |  | 2635 | 4874 | -2.197 | 0.089 | 1.309 | 0.03 | 0.94 | [100] |
| *Eoptychopterina*  *E. adnexa* | H&S | Sa | 177840 | 84 | 140 | 3 |  | 11672 | -2.197 | 0.089 | 1.309 | 0.03 | 0.94 | [101] |
| *E. elenae* | H&S | Sa | 72000 | Eosesatheris | 2273 |  | 3112 | 4253 | -2.197 | 0.089 | 1.309 | 0.03 | 0.94 | [97] |
| *E. antica* | H&S | Sa | 189448 | 221 | 318 | 5 | 8766 | 10856 | -2.197 | 0.089 | 1.309 | 0.03 | 0.94 | [101] |
| *E. mediata* | H&S | Sa | 265842 | 104 | 227 | 3 | 18749 | 32793 | -2.197 | 0.089 | 1.309 | 0.03 | 0.94 | [101] |
| *E. postica* | H&S | Sa | 241029 | 625 | 676 | 2 | 12743 | 38903 | -2.197 | 0.089 | 1.309 | 0.03 | 0.94 | [102] |
| *Proptychopterina*  *P. opinata* | H&S | Sa | 324696 | Eosesatheris | 9108 |  | 16471 | 14594 | -2.197 | 0.089 | 1.309 | 0.03 | 0.94 | [103] |
| Rhagionemestriidae  *Jurassinemestrinus*  *J. orientalis* | H&S | Ph | 167217 | 188 | 219 | 1 | 48068 | 20158 | -2.197 | 0.089 | 1.309 | 0.03 | 0.94 | [67] |
| Rhagionempididae  *Ussatchovia*  *U. gracilenta* | H&S | Ph | 203148 | 232 | 305 | 1 | 23364 | 18758 | -2.197 | 0.089 | 1.309 | 0.03 | 0.94 | [104] |
| *U. robusta* | H&S | Ph | 231324 | 321 | 375 | 1 | 16833 | 12261 | -2.197 | 0.089 | 1.309 | 0.03 | 0.94 | [104] |
| Rhagionidae  *Achrysopilus*  *A. neimenguensis* | E&S | Pr | 438256 | 276 | 341 | 1 | 53600 | 27306 | -2.197 | 0.089 | 1.309 | 0.03 | 0.94 | [105] |
| *Daohugorhagio*  *D. elongatus* | E&S | Pr | 282528 | 404 | 437 | 1 | 34233 | 16721 | -2.197 | 0.089 | 1.309 | 0.03 | 0.94 | [106] |
| *Lithorhagio*  *L. megalocephalus* | E&S | Pr | 406000 | 355 | 404 | 1 | 50779 | 26312 | -2.197 | 0.089 | 1.309 | 0.03 | 0.94 | [107] |
| *Palaeoarthroteles*  *P. jurassicus* | E&S | Pr | 330621 | 141 | 187 | 1 | 29886 | 20657 | -2.197 | 0.089 | 1.309 | 0.03 | 0.94 | [108] |
| *P. pallidus* | E&S | Pr | 225412 | 125 | 156 | 1 | 33568 | 26617 | -2.197 | 0.089 | 1.309 | 0.03 | 0.94 | [108] |
| *Palaeobolbomyia*  *P. sinica* | E&S | Pr | 200015 | 280 | 381 | 1 |  | 62870 | -2.197 | 0.089 | 1.309 | 0.03 | 0.94 | [104] |
| *Parachrysopilus*  *P. jurassicus* | E&S | Pr | 274975 | 351 | 400 | 1 | 11898 | 12437 | -2.197 | 0.089 | 1.309 | 0.03 | 0.94 | [106] |
| *Protorhagio*  *P. parvus* | E&S | Pr | 318444 | 216 | 296 | 1 | 24062 | 15571 | -2.197 | 0.089 | 1.309 | 0.03 | 0.94 | [107] |
| *Sinorhagio*  *S. daohugouensis* | E&S | Pr | 297686 | 215 | 265 | 1 | 52556 | 41396 | -2.197 | 0.089 | 1.309 | 0.03 | 0.94 | [109] |
| *S. sinuatus* | E&S | Pr | 310222 | 334 | 337 | 1 | 35396 | 21629 | -2.197 | 0.089 | 1.309 | 0.03 | 0.94 | [106] |
| *Trichorhagio*  *T. gregarius* | E&S | Pr | 425113 | 252 | 287 | 1 | 12693 | 8798 | -2.197 | 0.089 | 1.309 | 0.03 | 0.94 | [106] |
| Strashilidae  *Strashila*  *S. daohugouensis* | H | Ph | 95700 | 247 | 323 | 2 | 32362 | - | -2.197 | 0.089 | 1.309 | 0.03 | 0.94 | [110] |
| *Vosila*  *V. sinensis* | H | Ph | 370370 | 55 | 318 | 5.7 | 25765 | - | -2.197 | 0.089 | 1.309 | 0.03 | 0.94 | [110] |
| Tanyderidae  *Praemacrochile*  *P. ansorgei* | H&S | Sa | 348816 | 595 | 647 | 2 | 15391 | 16022 | -2.197 | 0.089 | 1.309 | 0.03 | 0.94 | [111] |
| *P. chinensis* | H&S | Sa | 368629 | 706 | 745 | 1 | 24415 | 20909 | -2.197 | 0.089 | 1.309 | 0.03 | 0.94 | [111] |
| *P. dryasis* | H&S | Sa | 121296 | 752 | 785 | 1 | 19018 | 10505 | -2.197 | 0.089 | 1.309 | 0.03 | 0.94 | [112] |
| *P. ovalum* | H&S | Sa | 123240 | 424 | 448 | 1 |  | 7148 | -2.197 | 0.089 | 1.309 | 0.03 | 0.94 | [112] |
| *Protanyderus*  *P. astictum* | H&S | Sa | 146304 | 879 | 911 | 1 | 42828 | 36930 | -2.197 | 0.089 | 1.309 | 0.03 | 0.94 | [112] |
| *P. vulcanium* | H&S | Sa | 210750 | 638 | 677 | 1 |  | 52740 | -2.197 | 0.089 | 1.309 | 0.03 | 0.94 | [112] |
| Trichoceridae  *Eotrichocera*  *E. (Archaeotrichocera) amabilis* | E&S | Sa | 616420 | 261 | 283 | 1 | 5856 | 5529 | -2.197 | 0.089 | 1.309 | 0.03 | 0.94 | [113] |
| *E. (Archaeotrichocera) conica* | E&S | Sa |  |  |  |  |  |  | -2.197 | 0.089 | 1.309 | 0.03 | 0.94 | [114] |
| *E. (Archaeotrichocera) ephemera* | E&S | Sa | 328520 | 507 | 541 | 1 | 12652 | 14209 | -2.197 | 0.089 | 1.309 | 0.03 | 0.94 | [115] |
| *E. (Archaeotrichocera) longensis* | E&S | Sa | 654922 | 566 | 605 | 1 | 35900 | 32176 | -2.197 | 0.089 | 1.309 | 0.03 | 0.94 | [113] |
| *E. (Archaeotrichocera) spatiosa* | E&S | Sa | 389340 | 626 | 688 | 2 | 26782 | 39533 | -2.197 | 0.089 | 1.309 | 0.03 | 0.94 | [102] |
| *Tanyochoreta*  *T. (Sinotrichocera) parva* | E&S | Sa | 462938 | 569 | 643 | 1 | 27839 | 18022 | -2.197 | 0.089 | 1.309 | 0.03 | 0.94 | [115] |
| *T. (Tanyochoreta) chifengica* | E&S | Sa | 319194 | 478 | 512 | 1 | 11598 | 12861 | -2.197 | 0.089 | 1.309 | 0.03 | 0.94 | [115] |
| *T. (Tanyochoreta) integera* | E&S | Sa | 394240 | 133 | 166 | 1 | 10106 | 8719 | -2.197 | 0.089 | 1.309 | 0.03 | 0.94 | [115] |
| *Zherikhinina*  *Z. reni* | E&S | Sa |  |  |  |  |  |  | -2.197 | 0.089 | 1.309 | 0.03 | 0.94 | [114] |
| **Embiidina**  Sinembiidae  *Juraembia*  *J. ningchengensis* | S | Ph | 407838 | 93 | 175 | 2 | 30004 |  | -2.14 | 0.036 | 1.331 | 0.011 | 0.94 | [116] |
| Sinembia  S. rossi | S | Ph | 417186 | 61 | 134 | 2 | 9593 |  | -2.14 | 0.036 | 1.331 | 0.011 | 0.94 | [116] |
| **Ephemeroptera**  Fuyoidae  *Fuyous*  *F. gregarius* | H | Ph |  |  |  |  |  |  | -2.14 | 0.036 | 1.331 | 0.011 | 0.94 | [117] |
| Hexagenitidae  *Shantous*  *S. lacustris* | H | Ph |  |  |  |  |  |  | -2.14 | 0.036 | 1.331 | 0.011 | 0.94 | [117] |
| Siphlonuridae  *Cheirolgisca*  *C. ningchengensis* | H | Ph |  |  |  |  |  |  | -2.14 | 0.036 | 1.331 | 0.011 | 0.94 | [118] |
| *Olgisca*  *O. angusticubitis* | H | Ph |  |  |  |  |  |  | -2.14 | 0.036 | 1.331 | 0.011 | 0.94 | [118] |
| Siphluriscidae  *Jurassonurus*  *J. amoenus* | H | Ph | 85907 | 27 | 48 | 2 | 4529 | 5341 | -2.14 | 0.036 | 1.331 | 0.011 | 0.94 | [119] |
| *Multiramificans*  *M. ovalis* | H | Ph | 222256 | 28 | 61 | 2 | 14166 | 26278 | -2.14 | 0.036 | 1.331 | 0.011 | 0.94 | [120] |
| *Stackelbergisca*  *S. cylindrata* | H | Ph | 101906 | 377 | 404 | 1 | 38277 | - | -2.14 | 0.036 | 1.331 | 0.011 | 0.94 | [121] |
| **Grylloblattodea**  Bajanzhargalanidae  *Sinonele*  *S. fangi* | E&S | Pr | 484400 | 47 | 93 | 6 | 6985 | 10878 | -2.14 | 0.036 | 1.331 | 0.011 | 0.94 | [122] |
| *S. hei* | E&S | Pr | 116424 | 44 | 100 | 6 |  | 24937 | -2.14 | 0.036 | 1.331 | 0.011 | 0.94 | [122] |
| *S. mini* | E&S | Pr | 200464 | 25 | 75 | 3 |  | 19698 | -2.14 | 0.036 | 1.331 | 0.011 | 0.94 | [122] |
| *S. phasmoides* | E&S | Pr | 249197 | 22 | 71 | 6 |  | 15795 | -2.14 | 0.036 | 1.331 | 0.011 | 0.94 | [122] |
| Blattogryllidae  *Duoduo*  *D. qianae* | E&S | Pr | 399455 | 37 | 122 | 5 |  | 111452 | -2.14 | 0.036 | 1.331 | 0.011 | 0.94 | [123] |
| *Plesioblattogryllus*  *P. magnificus* | E&S | Pr | 641520 | 559 | 659 | 10 | 54103 |  | -2.14 | 0.036 | 1.331 | 0.011 | 0.94 | [124] |
| *P. minor* | E&S | Pr | 130986 | 16 | 127 | 10 |  | 16246 | -2.14 | 0.036 | 1.331 | 0.011 | 0.94 | [125] |
| Geinitziidae  *Geinitzia*  *G. aristovi* | E&S | Pr | 575050 | 10 | 132 | 5 |  | 101804 | -2.14 | 0.036 | 1.331 | 0.011 | 0.94 | [126] |
| *Shurabia*  *S. grandis* | E&S | Pr | 389940 | 107 | 185 | 5 | 34926 |  | -2.14 | 0.036 | 1.331 | 0.011 | 0.94 | [126] |
| *Sinosepididontus*  *S. chifengensis* | E&S | Pr | 428400 | 164 | 267 | 5 | 14314 |  | -2.14 | 0.036 | 1.331 | 0.011 | 0.94 | [126] |
| Juraperlidae  *Juraperla*  *J. daohugouensis* | E&S | Pr | 81720 | 238 | 320 | 4 |  | 28044 | -2.14 | 0.036 | 1.331 | 0.011 | 0.94 | [127] |
| *J. grandis* | E&S | Pr | 250125 | 31 | 83 | 5 | 14165 | 24726 | -2.14 | 0.036 | 1.331 | 0.011 | 0.94 | [128] |
| **Hemiptera**  Incertae sedis  *Serecoleus*  *S. nadbitovae* | S | Ph |  |  |  |  |  |  | -2.765 | 0.135 | 1.423 | 0.041 | 0.97 | [17] |
| Corixidae  *Daohugocorixa*  *D. vulcanica* | H | Ph | 112518 | 169 | 198 | 1 | 10493 | 3494 | -2.765 | 0.135 | 1.423 | 0.041 | 0.97 | [129] |
| *Karataviella*  *K. popovi* | H | Ph | 93636 | 138 | 155 | 1 | 14432 | 6118 | -2.765 | 0.135 | 1.423 | 0.041 | 0.97 | [129] |
| Fulgoridiidae  *Fenghuangor*  *F. imperator* | E&S | Ph | 290966 | 534 | 578 | 1 |  | 184881 | -2.765 | 0.135 | 1.423 | 0.041 | 0.97 | [130] |
| Miridae  *Mirivena*  *M. robusta* | S | Ph | 335543 | 40 | 80 | 1 | 54671 |  | -2.765 | 0.135 | 1.423 | 0.041 | 0.97 | [131] |
| Oviparosiphidae  *Daoaphis*  *D. magnalata* | E&S | Ph | 376508 | 318 | 375 | 5 | 24848 |  | -2.765 | 0.135 | 1.423 | 0.041 | 0.97 | [132] |
| Pachymeridiidae  *Corollpachymeridium*  *C. heteroneurus* | S | Ph |  |  |  |  |  |  | -2.765 | 0.135 | 1.423 | 0.041 | 0.97 | [133] |
| *Nitoculus*  *N. regillus* | S | Ph | 103761 | 15 | 43 | 1 | 20086 |  | -2.765 | 0.135 | 1.423 | 0.041 | 0.97 | [134] |
| *Peregrinpachymeridium*  *P. comitcola* | S | Ph |  |  |  |  |  |  | -2.765 | 0.135 | 1.423 | 0.041 | 0.97 | [133] |
| *Sinopachymeridium*  *S. popovi* | S | Ph | 324480 | 461 | 533 | 1 | 56649 |  | -2.765 | 0.135 | 1.423 | 0.041 | 0.97 | [135] |
| *[135] Viriosinervis*  *V. stolidus* | S | Ph | 100188 | 15 | 54 | 1 | 38346 |  | -2.765 | 0.135 | 1.423 | 0.041 | 0.97 | [134] |
| Palaeontinidae  *Abrocossus*  *A. longus* | E&S | Ph | 814800 | 557 | 662 | 10 |  | 119146 | -2.765 | 0.135 | 1.423 | 0.041 | 0.97 | [136] |
| *Cladocossus*  *C. undulatus* | E&S | Ph | 81510 | 261 | 311 | 5 |  | 30222 | -2.765 | 0.135 | 1.423 | 0.041 | 0.97 | [137] |
| *Cricocossus*  *C. paradoxus* | E&S | Ph | 79730 | 273 | 318 | 5 |  | 30586 | -2.765 | 0.135 | 1.423 | 0.041 | 0.97 | [137] |
| *Daohugoucossus*  *D. lii* | E&S | Ph | 148167 | 295 | 370 | 10 |  | 39951 | -2.765 | 0.135 | 1.423 | 0.041 | 0.97 | [138] |
| *D. parallelivenius* | E&S | Ph | 141966 | 33 | 147 | 10 |  | 51185 | -2.765 | 0.135 | 1.423 | 0.041 | 0.97 | [138] |
| *D. shii* | E&S | Ph | 310147 | 134 | 194 | 10 | 16280 | 35683 | -2.765 | 0.135 | 1.423 | 0.041 | 0.97 | [138] |
| *D. solutus* | E&S | Ph | 160564 | 76 | 150 | 5 |  | 71271 | -2.765 | 0.135 | 1.423 | 0.041 | 0.97 | [139] |
| *Eoiocossus*  *E. conchatus* | E&S | Ph |  |  |  |  |  |  | -2.765 | 0.135 | 1.423 | 0.041 | 0.97 | [140] |
| *E. pteroideus* | E&S | Ph |  |  |  |  |  |  | -2.765 | 0.135 | 1.423 | 0.041 | 0.97 | [140] |
| *E. validus* | E&S | Ph | 257560 | 583 | 640 | 10 |  | 125167 | -2.765 | 0.135 | 1.423 | 0.041 | 0.97 | [140] |
| *Gansucossus*  *G. typicus* | E&S | Ph | 189392 | 63 | 142 | 5 |  | 84953 | -2.765 | 0.135 | 1.423 | 0.041 | 0.97 | [139] |
| *Kallicossus*  *K. ningchengensis* | E&S | Ph |  |  |  |  |  |  | -2.765 | 0.135 | 1.423 | 0.041 | 0.97 | [141] |
| *Martynovocossus*  *M. ancylivenius* | E&S | Ph |  |  |  |  |  |  | -2.765 | 0.135 | 1.423 | 0.041 | 0.97 | [142] |
| *M. bellus* | E&S | Ph |  |  |  |  |  |  | -2.765 | 0.135 | 1.423 | 0.041 | 0.97 | [143] |
| *M. cheni* | E&S | Ph | 194628 | 294 | 358 | 10 |  | 20583 | -2.765 | 0.135 | 1.423 | 0.041 | 0.97 | [143] |
| *M. decorus* | E&S | Ph | 292243 | 96 | 222 | 10 |  | 59483 | -2.765 | 0.135 | 1.423 | 0.041 | 0.97 | [143] |
| *M. punctulosus* | E&S | Ph |  |  |  |  |  |  | -2.765 | 0.135 | 1.423 | 0.041 | 0.97 | [143] |
| *Neimenggucossus*  *N. normalis* | E&S | Ph | 697236 | 287 | 360 | 5 |  | 54137 | -2.765 | 0.135 | 1.423 | 0.041 | 0.97 | [136] |
| *Ningchengia*  *N. aspera* | E&S | Ph | 215775 | 379 | 437 | 5 |  | 45896 | -2.765 | 0.135 | 1.423 | 0.041 | 0.97 | [144] |
| *N. minuta* | E&S | Ph | 182112 | 2 | 55 | 3 |  | 46227 | -2.765 | 0.135 | 1.423 | 0.041 | 0.97 | [145] |
| *Palaeontinodes*  *P. angarensis* | E&S | Ph | 197136 | 17 | 82 | 10 |  | 30550 | -2.765 | 0.135 | 1.423 | 0.041 | 0.97 | [146] |
| *P. daohugouensis* | E&S | Ph | 839209 | 519 | 655 | 5 |  | 133474 | -2.765 | 0.135 | 1.423 | 0.041 | 0.97 | [136] |
| *P. locellus* | E&S | Ph | 832788 | 80 | 195 | 10 |  | 125655 | -2.765 | 0.135 | 1.423 | 0.041 | 0.97 | [136] |
| *P. reshuitangensis* | E&S | Ph |  |  |  |  |  |  | -2.765 | 0.135 | 1.423 | 0.041 | 0.97 | [147] |
| *P. separatus* | E&S | Ph | 213129 | 282 | 378 | 10 |  | 33016 | -2.765 | 0.135 | 1.423 | 0.041 | 0.97 | [136] |
| *P. shabarovi* | E&S | Ph | 221130 | 406 | 498 | 10 |  | 56851 | -2.765 | 0.135 | 1.423 | 0.041 | 0.97 | [145] |
| *Papilioncossus*  *P. conchatus* | E&S | Ph | 106945 | 45 | 96 | 10 |  | 36377 | -2.765 | 0.135 | 1.423 | 0.041 | 0.97 | [145] |
| *P. giganteus* | E&S | Ph | 101802 | 245 | 284 | 10 |  | 32268 | -2.765 | 0.135 | 1.423 | 0.041 | 0.97 | [148] |
| *P. pteroideus* | E&S | Ph | 93438 | 68 | 111 | 10 |  | 28377 | -2.765 | 0.135 | 1.423 | 0.041 | 0.97 | [145] |
| *Plachutella*  *P. magica* | E&S | Ph | 197568 | 308 | 376 | 5 |  | 66367 | -2.765 | 0.135 | 1.423 | 0.041 | 0.97 | [145] |
| *Sinopalaeocossus*  *S. amoenus* | E&S | Ph | 336713 | 385 | 446 | 10 |  | 16471 | -2.765 | 0.135 | 1.423 | 0.041 | 0.97 | [149] |
| *S. eumorphus* | E&S | Ph | 281055 | 436 | 547 | 5 |  | 103086 | -2.765 | 0.135 | 1.423 | 0.041 | 0.97 | [150] |
| *S. fangi* | E&S | Ph |  |  |  |  |  |  | -2.765 | 0.135 | 1.423 | 0.041 | 0.97 | [149] |
| *S. laevis* | E&S | Ph |  |  |  |  |  |  | -2.765 | 0.135 | 1.423 | 0.041 | 0.97 | [149] |
| *S. longicaulis* | E&S | Ph | 286026 | 98 | 230 | 10 |  | 81432 | -2.765 | 0.135 | 1.423 | 0.041 | 0.97 | [151] |
| *S. trinervus* | E&S | Ph | 148551 | 420 | 498 | 5 |  | 67633 | -2.765 | 0.135 | 1.423 | 0.041 | 0.97 | [152] |
| *Suljuktocossus*  *S. chifengensis* | E&S | Ph | 853702 | 528 | 706 | 10 |  | 143410 | -2.765 | 0.135 | 1.423 | 0.041 | 0.97 | [136] |
| *S. coloratus* | E&S | Ph | 205200 | 263 | 328 | 5 |  | 52776 | -2.765 | 0.135 | 1.423 | 0.041 | 0.97 | [145] |
| *S. yinae* | E&S | Ph |  |  |  |  |  |  | -2.765 | 0.135 | 1.423 | 0.041 | 0.97 | [153] |
| *Synapocossus*  *S. sciacchitanoae* | E&S | Ph | 169598 | 560 | 674 | 10 | 17811 | 28562 | -2.765 | 0.135 | 1.423 | 0.041 | 0.97 | [154] |
| Procercopidae  *Anthoscytina*  *A. brevineura* | E&S | Ph | 282820 | 368 | 398 | 2 |  | 26667 | -2.765 | 0.135 | 1.423 | 0.041 | 0.97 | [155] |
| *A. elegans* | E&S | Ph | 248400 | 761 | 698 | 2 |  | 23688 | -2.765 | 0.135 | 1.423 | 0.041 | 0.97 | [155] |
| *A. perpetua* | E&S | Ph | 563387 | 477 | 504 | 2 | 7416 |  | -2.765 | 0.135 | 1.423 | 0.041 | 0.97 | [156] |
| *A. daidaleos* | E&S | Ph | 471485 | 20 | 83 | 2 |  | 50246 | -2.765 | 0.135 | 1.423 | 0.041 | 0.97 | [157] |
| *Jurocercopis*  *J. grandis* | E&S | Ph | 184819 | 204 | 264 | 4 | 25522 | 26755 | -2.765 | 0.135 | 1.423 | 0.041 | 0.97 | [158] |
| *Titanocercopis*  *T. borealis* | E&S | Ph |  |  |  |  |  |  | -2.765 | 0.135 | 1.423 | 0.041 | 0.97 | [159] |
| Progonocimicidae  *Cicadocoris*  *C. anisomeridis* | S | Ph | 120786 | 105 | 128 | 1 | 4941 | 3016 | -2.765 | 0.135 | 1.423 | 0.041 | 0.97 | [160] |
| *C. assimilis* | S | Ph |  |  |  |  |  |  | -2.765 | 0.135 | 1.423 | 0.041 | 0.97 | [161] |
| *C. brunneus* | S | Ph |  |  |  |  |  |  | -2.765 | 0.135 | 1.423 | 0.041 | 0.97 | [16] |
| *Mesocimex*  *M. lini* | S | Ph | 392786 | 140 | 184 | 1 | 20048 | 16740 | -2.765 | 0.135 | 1.423 | 0.041 | 0.97 | [162] |
| *M. parvus* | S | Ph | 214896 | 199 | 260 | 1 | 13998 | 10160 | -2.765 | 0.135 | 1.423 | 0.041 | 0.97 | [163] |
| *M. sinensis* | S | Ph |  |  |  |  |  |  | -2.765 | 0.135 | 1.423 | 0.041 | 0.97 | [16] |
| *M. varians* | S | Ph |  |  |  |  |  |  | -2.765 | 0.135 | 1.423 | 0.041 | 0.97 | [164] |
| Protopsyllidiidae  *Poljanka*  *P. hirsuta* | E&S | Ph | 343980 | 362 | 452 | 2 | 22397 | 16780 | -2.765 | 0.135 | 1.423 | 0.041 | 0.97 | [165] |
| *P. strigosa* | E&S | Ph | 615772 | 43 | 194 | 1 | 39589 | 64892 | -2.765 | 0.135 | 1.423 | 0.041 | 0.97 | [166] |
| Rhopalidae  *Grandicaputus*  *G. bipunctatus* | S | Ph |  |  |  |  |  |  | -2.765 | 0.135 | 1.423 | 0.041 | 0.97 | [167] |
| *Longiclavula*  *L. calvata* | S | Ph | 275328 | 64 | 100 | 1 | 40839 | 20807 | -2.765 | 0.135 | 1.423 | 0.041 | 0.97 | [168] |
| *Miracorizus*  *M. punctatus* | S | Ph | 289800 | 19 | 58 | 1 | 39476 |  | -2.765 | 0.135 | 1.423 | 0.041 | 0.97 | [168] |
| *Originicorizus*  *O. pyriformis* | S | Ph |  |  |  |  |  |  | -2.765 | 0.135 | 1.423 | 0.041 | 0.97 | [167] |
| *Quatlocellus*  *Q. liae* | S | Ph |  |  |  |  |  |  | -2.765 | 0.135 | 1.423 | 0.041 | 0.97 | [167] |
| Sinoalidae  *Jiania*  *J. crebra* | E&S | Ph | 355840 | 278 | 319 | 4 | 12534 |  | -2.765 | 0.135 | 1.423 | 0.041 | 0.97 | [169] |
| *J. gracila* | E&S | Ph | 378336 | 149 | 205 | 4 | 15933 |  | -2.765 | 0.135 | 1.423 | 0.041 | 0.97 | [169] |
| *Luanpingia*  *L. daohugouensis* | E&S | Ph |  |  |  |  |  |  | -2.765 | 0.135 | 1.423 | 0.041 | 0.97 | [170] |
| *Shufania*  *S. hani* | E&S | Ph | 352944 | 524 | 621 | 1 |  | 111286 | -2.765 | 0.135 | 1.423 | 0.041 | 0.97 | [171] |
| *Sinoala*  *S. parallelivena* | E&S | Ph | 343896 | 173 | 208 | 2 | 8757 |  | -2.765 | 0.135 | 1.423 | 0.041 | 0.97 | [169] |
| *Stictocercopis*  *S. wuhuaensis* | E&S | Ph | 561450 | 260 | 307 | 2 | 22348 |  | -2.765 | 0.135 | 1.423 | 0.041 | 0.97 | [172] |
| Sinojuraphididae  *Sinojuraphis*  *S. ningchengensis* | E&S | Ph | 415496 | 176 | 236 | 2 | 7568 |  | -2.765 | 0.135 | 1.423 | 0.041 | 0.97 | [173] |
| Tettigarctidae  *Hirtaprosbole*  *H. erromera* | E&S&A | Ph |  |  |  |  |  |  | -2.765 | 0.135 | 1.423 | 0.041 | 0.97 | [174] |
| *Maculaprosbole*  *M. zhengi* | E&S&A | Ph | 267810 | 407 | 434 | 2 |  | 51825 | -2.765 | 0.135 | 1.423 | 0.041 | 0.97 | [175] |
| *Sanmai*  *S. kongi* | E&S&A | Ph | 122220 | 125 | 181 | 5 |  | 13156 | -2.765 | 0.135 | 1.423 | 0.041 | 0.97 | [176] |
| *S. mengi* | E&S&A | Ph | 116432 | 450 | 550 | 5 | 56902 | 53400 | -2.765 | 0.135 | 1.423 | 0.041 | 0.97 | [176] |
| *S. xuni* | E&S&A | Ph |  | 597 | 650 | 5 |  | 14706 | -2.765 | 0.135 | 1.423 | 0.041 | 0.97 | [176] |
| *Shuraboprosbole*  *S. daohugouensis* | E&S&A | Ph | 137816 | 292 | 401 | 10 | 25299 | 35505 | -2.765 | 0.135 | 1.423 | 0.041 | 0.97 | [177] |
| *S. media* | E&S&A | Ph | 134674 | 367 | 441 | 5 |  | 46566 | -2.765 | 0.135 | 1.423 | 0.041 | 0.97 | [177] |
| *S. minuta* | E&S&A | Ph | 190320 | 313 | 446 | 5 | 55929 |  | -2.765 | 0.135 | 1.423 | 0.041 | 0.97 | [177] |
| *Sunotettigarcta*  *S. hirsute* | E&S&A | Ph | 287984 | 401 | 438 | 5 | 42561 |  | -2.765 | 0.135 | 1.423 | 0.041 | 0.97 | [178] |
| *Tianyuprosbole*  *T. zhengi* | E&S&A | Ph | 453492 | 381 | 515 | 5 |  | 113359 | -2.765 | 0.135 | 1.423 | 0.041 | 0.97 | [179] |
| Vetanthocoridae  *Pumilanthocoris*  *P. gracilis* | S | Pr |  |  |  |  |  |  | -2.765 | 0.135 | 1.423 | 0.041 | 0.97 | [179] |
| *P. obesus* | S | Pr |  |  |  |  |  |  | -2.765 | 0.135 | 1.423 | 0.041 | 0.97 | [179] |
| **Hymenoptera**  Incertae sedis  *Prolyda*  *P. dimidia* | S | Ph | 109564 | 627 | 669 | 2 | 10745 | 9577 | -2.375 | 0.08 | 1.456 | 0.028 | 0.95 | [180] |
| P. elegantula | S | Ph | 104544 | 652 | 696 | 2 | 12295 |  | -2.375 | 0.08 | 1.456 | 0.028 | 0.95 | [180] |
| Anomopterellidae  *Anomopterella*  *A. ampla* | S | Pa&Pr | 171613 | 358 | 385 | 1 | 10312 |  | -2.375 | 0.08 | 1.456 | 0.028 | 0.95 | [181] |
| *A. brachystelis* | S | Pa&Pr | 347259 | 27 | 75 | 1 | 35596 | 19223 | -2.375 | 0.08 | 1.456 | 0.028 | 0.95 | [181] |
| *A. brevis* | S | Pa&Pr | 86536 | 53 | 104 | 1 | 13066 | 11147 | -2.375 | 0.08 | 1.456 | 0.028 | 0.95 | [182] |
| *A. coalita* | S | Pa&Pr | 334048 | 37 | 84 | 1 | 18155 | 15039 | -2.375 | 0.08 | 1.456 | 0.028 | 0.95 | [181] |
| *A. divergens* | S | Pa&Pr | 268956 | 32 | 63 | 1 | 6996 | 5802 | -2.375 | 0.08 | 1.456 | 0.028 | 0.95 | [181] |
| *A. huangi* | S | Pa&Pr |  |  |  |  |  |  | -2.375 | 0.08 | 1.456 | 0.028 | 0.95 | [183] |
| *A. ovalis* | S | Pa&Pr | 253011 | 33 | 61 | 1 | 5784 | 4377 | -2.375 | 0.08 | 1.456 | 0.028 | 0.95 | [181] |
| *A. pygmea* | S | Pa&Pr | 85176 | 17 | 59 | 1 | 8849 | 6559 | -2.375 | 0.08 | 1.456 | 0.028 | 0.95 | [182] |
| *Choristopterella*  *C. stenocera* | S | Pa&Pr |  |  |  |  |  |  | -2.375 | 0.08 | 1.456 | 0.028 | 0.95 | [184] |
| *Synaphopterella*  *S. patula* | S | Pa&Pr | 396772 | 119 | 159 | 1 | 10914 | 5944 | -2.375 | 0.08 | 1.456 | 0.028 | 0.95 | [181] |
| Daohugoidae  *Daohugoa*  *D. tobiasi* | S | Ph | 264231 | 269 | 364 | 5 | 17651 |  | -2.375 | 0.08 | 1.456 | 0.028 | 0.95 | [185] |
| Ephialtitidae  *Asiephialtites*  *A. lini* | S | Pa&Pr | 147519 | 40 | 199 | 5 | 15214 |  | -2.375 | 0.08 | 1.456 | 0.028 | 0.95 | [186] |
| *Karataus*  *K. daohugouensis* | S | Pa&Pr | 80968 | 43 | 74 | 2 | 10541 | 5487 | -2.375 | 0.08 | 1.456 | 0.028 | 0.95 | [187] |
| *K. exilis* | S | Pa&Pr | 58104 | 63 | 92 | 2 | 6079 | 4111 | -2.375 | 0.08 | 1.456 | 0.028 | 0.95 | [187] |
| *K. orientalis* | S | Pa&Pr | 61864 | 91 | 123 | 2 | 7814 | 4759 | -2.375 | 0.08 | 1.456 | 0.028 | 0.95 | [187] |
| *K. strenuus* | S | Pa&Pr | 64380 | 44 | 98 | 2 | 10703 |  | -2.375 | 0.08 | 1.456 | 0.028 | 0.95 | [187] |
| *K. vigoratus* | S | Pa&Pr | 57054 | 32 | 68 | 2 | 7053 |  | -2.375 | 0.08 | 1.456 | 0.028 | 0.95 | [187] |
| *Praeproapocritus*  *P. flexus* | S | Pa&Pr | 326040 | 105 | 131 | 5 | 21294 | 11416 | -2.375 | 0.08 | 1.456 | 0.028 | 0.95 | [188] |
| *P. vulgatus* | S | Pa&Pr | 156546 | 46 | 180 | 5 | 22797 |  | -2.375 | 0.08 | 1.456 | 0.028 | 0.95 | [186] |
| *Proapocritus*  *P. atropus* | S | Pa&Pr | 161721 | 112 | 220 | 5 | 9356 |  | -2.375 | 0.08 | 1.456 | 0.028 | 0.95 | [186] |
| *P. bialatus* | S | Pa&Pr | 309792 | 325 | 350 | 1 | 12675 |  | -2.375 | 0.08 | 1.456 | 0.028 | 0.95 | [189] |
| *P. densipediculus* | S | Pa&Pr | 165092 | 55 | 163 | 5 | 26095 | 21558 | -2.375 | 0.08 | 1.456 | 0.028 | 0.95 | [186] |
| *P. elegans* | S | Pa&Pr | 133848 | 124 | 267 | 5 | 27761 |  | -2.375 | 0.08 | 1.456 | 0.028 | 0.95 | [186] |
| *P. formosus* | S | Pa&Pr | 140114 | 83 | 229 | 5 | 29670 |  | -2.375 | 0.08 | 1.456 | 0.028 | 0.95 | [186] |
| *P. longantennatus* | S | Pa&Pr | 248952 | 16 | 260 | 5 | 29889 |  | -2.375 | 0.08 | 1.456 | 0.028 | 0.95 | [186] |
| *P. parallelus* | S | Pa&Pr | 101003 | 36 | 68 | 1 | 14990 | 10805 | -2.375 | 0.08 | 1.456 | 0.028 | 0.95 | [188] |
| *P. sculptus* | S | Pa&Pr | 137709 | 293 | 402 | 5 | 12975 |  | -2.375 | 0.08 | 1.456 | 0.028 | 0.95 | [186] |
| *Proephialtitia*  *P. acantha* | S | Pa&Pr | 355300 | 165 | 203 | 5 | 6293 |  | -2.375 | 0.08 | 1.456 | 0.028 | 0.95 | [190] |
| *P. tenuata* | S | Pa&Pr | 78455 | 18 | 39 | 5 | 3428 | 1116 | -2.375 | 0.08 | 1.456 | 0.028 | 0.95 | [190] |
| *Stephanogaster*  *S. ningchengensis* | S | Pa&Pr | 152490 | 71 | 109 | 2 | 16424 | 9428 | -2.375 | 0.08 | 1.456 | 0.028 | 0.95 | [191] |
| *S. pristinus* | S | Pa&Pr | 233640 | 149 | 299 | 5 | 25501 |  | -2.375 | 0.08 | 1.456 | 0.028 | 0.95 | [186] |
| *S. rasnitsyni* | S | Pa&Pr |  |  |  |  |  |  | -2.375 | 0.08 | 1.456 | 0.028 | 0.95 | [191] |
| *Symphytopterus*  *S. graciler* | S | Pa&Pr |  |  |  |  |  |  | -2.375 | 0.08 | 1.456 | 0.028 | 0.95 | [192] |
| Heloridae  *Archaeohelorus*  *A. hoi* | S | Pa&Pr | 396274 | 321 | 345 | 1 | 9847 | 5326 | -2.375 | 0.08 | 1.456 | 0.028 | 0.95 | [193] |
| *A. polyneurus* | S | Pa&Pr | 104512 | 679 | 708 | 1 | 12750 | 10701 | -2.375 | 0.08 | 1.456 | 0.028 | 0.95 | [194] |
| *A. tensus* | S | Pa&Pr | 373500 | 689 | 716 | 1 | 15799 |  | -2.375 | 0.08 | 1.456 | 0.028 | 0.95 | [194] |
| Karatavitidae  *Karatavites*  *K. junfengi* | S | Pa&Pr | 314500 | 350 | 558 | 5 | 31677 |  | -2.375 | 0.08 | 1.456 | 0.028 | 0.95 | [186] |
| *K. medius* | S | Pa&Pr |  |  |  |  |  |  | -2.375 | 0.08 | 1.456 | 0.028 | 0.95 | [195] |
| *K. ningchengensis* | S | Pa&Pr | 556944 | 413 | 452 | 1 | 36553 |  | -2.375 | 0.08 | 1.456 | 0.028 | 0.95 | [196] |
| *Postxiphydria*  *P. daohugouensis* | S | Pa&Pr | 228765 | 33 | 203 | 5 | 34094 |  | -2.375 | 0.08 | 1.456 | 0.028 | 0.95 | [186] |
| *P. ningchengensis* | S | Pa&Pr | 231242 | 288 | 436 | 5 | 36986 |  | -2.375 | 0.08 | 1.456 | 0.028 | 0.95 | [186] |
| *Postxiphydroides*  *P. strenuus* | S | Pa&Pr | 292023 | 361 | 598 | 5 | 41949 |  | -2.375 | 0.08 | 1.456 | 0.028 | 0.95 | [186] |
| *Praeparyssites*  *P. orientalis* | S | Pa&Pr | 482658 | 227 | 336 | 1 | 48610 |  | -2.375 | 0.08 | 1.456 | 0.028 | 0.95 | [197] |
| *Praeratavites*  *P. daohugou* | S | Pa&Pr | 307643 | 398 | 566 | 5 | 24533 |  | -2.375 | 0.08 | 1.456 | 0.028 | 0.95 | [197] |
| *P. perspicuus* | S | Pa&Pr | 288075 | 29 | 215 | 5 | 27588 | 17198 | -2.375 | 0.08 | 1.456 | 0.028 | 0.95 | [186] |
| *P. rasnitsyni* | S | Pa&Pr | 139278 | 211 | 230 | 1 | 6792 | 5835 | -2.375 | 0.08 | 1.456 | 0.028 | 0.95 | [196] |
| *P. wuhuaensis* | S | Pa&Pr | 199101 | 207 | 305 | 5 | 31056 | 23401 | -2.375 | 0.08 | 1.456 | 0.028 | 0.95 | [186] |
| *Praeratavitoides*  *P. amabilis* | S | Pa&Pr | 259560 | 151 | 355 | 5 | 61707 | 27956 | -2.375 | 0.08 | 1.456 | 0.028 | 0.95 | [186] |
| Kuafuidae  *Kuafua*  *K. polyneura* | S | Pa&Pr | 142680 | 243 | 393 | 5 | 15427 |  | -2.375 | 0.08 | 1.456 | 0.028 | 0.95 | [186] |
| *Megalodontesidae*  *Brevisiricius*  *B. partialis* | S | Ph | 214948 | 218 | 233 | 1 | 3706 | 2802 | -2.375 | 0.08 | 1.456 | 0.028 | 0.95 | [198] |
| *Limbisiricius*  *L. aequalis* | S | Ph | 287718 | 419 | 432 | 1 | 4750 |  | -2.375 | 0.08 | 1.456 | 0.028 | 0.95 | [198] |
| *L. complanatus* | S | Ph | 245448 | 193 | 211 | 1 | 8543 | 4637 | -2.375 | 0.08 | 1.456 | 0.028 | 0.95 | [198] |
| Mesoserphidae  *Apiciserphus*  *A. augustus* | S | Pa&Pr |  |  |  |  |  |  | -2.375 | 0.08 | 1.456 | 0.028 | 0.95 | [199] |
| *Basiserphus*  *B. longus* | S | Pa&Pr |  |  |  |  |  |  | -2.375 | 0.08 | 1.456 | 0.028 | 0.95 | [199] |
| *Choriserphus*  *C. bellus* | S | Pa&Pr |  |  |  |  |  |  | -2.375 | 0.08 | 1.456 | 0.028 | 0.95 | [199] |
| *C. gigantus* | S | Pa&Pr |  |  |  |  |  |  | -2.375 | 0.08 | 1.456 | 0.028 | 0.95 | [199] |
| *Juraserphus*  *J. modicus* | S | Pa&Pr | 341000 | 318 | 375 | 2 | 16018 | 10074 | -2.375 | 0.08 | 1.456 | 0.028 | 0.95 | [200] |
| *Karataoserphus*  *K. adaequatus* | S | Pa&Pr |  |  |  |  |  |  | -2.375 | 0.08 | 1.456 | 0.028 | 0.95 | [199] |
| *K. gracilentus* | S | Pa&Pr |  |  |  |  |  |  | -2.375 | 0.08 | 1.456 | 0.028 | 0.95 | [199] |
| *Mesoserphus*  *M. venustus* | S | Pa&Pr |  |  |  |  |  |  | -2.375 | 0.08 | 1.456 | 0.028 | 0.95 | [199] |
| *Novserphus*  *N. ningchengensis* | S | Pa&Pr |  |  |  |  |  |  | -2.375 | 0.08 | 1.456 | 0.028 | 0.95 | [199] |
| *Ozososerphus*  *O. cuboidus* | S | Pa&Pr |  |  |  |  |  |  | -2.375 | 0.08 | 1.456 | 0.028 | 0.95 | [199] |
| *O. lepidus* | S | Pa&Pr |  |  |  |  |  |  | -2.375 | 0.08 | 1.456 | 0.028 | 0.95 | [199] |
| *O. ovatus* | S | Pa&Pr |  |  |  |  |  |  | -2.375 | 0.08 | 1.456 | 0.028 | 0.95 | [199] |
| *Sinoserphus*  *S. flexilis* | S | Pa&Pr |  |  |  |  |  |  | -2.375 | 0.08 | 1.456 | 0.028 | 0.95 | [199] |
| *S. grossus* | S | Pa&Pr |  |  |  |  |  |  | -2.375 | 0.08 | 1.456 | 0.028 | 0.95 | [199] |
| *S. lillianae* | S | Pa&Pr | 223357 | 298 | 353 | 2 | 10685 | 6113 | -2.375 | 0.08 | 1.456 | 0.028 | 0.95 | [193] |
| *S. petilus* | S | Pa&Pr |  |  |  |  |  |  | -2.375 | 0.08 | 1.456 | 0.028 | 0.95 | [199] |
| *S. shihae* | S | Pa&Pr | 292670 | 331 | 365 | 2 | 11577 | 6898 | -2.375 | 0.08 | 1.456 | 0.028 | 0.95 | [193] |
| *S. wui* | S | Pa&Pr | 268224 | 589 | 627 | 2 | 6559 |  | -2.375 | 0.08 | 1.456 | 0.028 | 0.95 | [193] |
| *Yanliaoserphus*  *Y. jurassicus* | S | Pa&Pr | 289842 | 590 | 642 | 2 | 6608 | 3408 | -2.375 | 0.08 | 1.456 | 0.028 | 0.95 | [193] |
| Mirolydidae  *Mirolyda*  *M. hirta* | S | Ph | 320606 | 123 | 143 | 2 | 5871 |  | -2.375 | 0.08 | 1.456 | 0.028 | 0.95 | [201] |
| Nevaniidae  *Eonevania*  *E. robusta* | S | Pa&Pr | 166608 | 294 | 449 | 5 | 23335 |  | -2.375 | 0.08 | 1.456 | 0.028 | 0.95 | [186] |
| *Nevania*  *N. aspectabilis* | S | Pa&Pr | 262150 | 304 | 346 | 2 | 24154 |  | -2.375 | 0.08 | 1.456 | 0.028 | 0.95 | [202] |
| *N. delicata* | S | Pa&Pr | 236979 | 16 | 69 | 2 | 20190 | 13750 | -2.375 | 0.08 | 1.456 | 0.028 | 0.95 | [203] |
| *N. exquisita* | S | Pa&Pr | 416412 | 43 | 92 | 2 | 23045 | 11893 | -2.375 | 0.08 | 1.456 | 0.028 | 0.95 | [203] |
| *N. ferocula* | S | Pa&Pr | 339090 | 311 | 396 | 2 | 33426 |  | -2.375 | 0.08 | 1.456 | 0.028 | 0.95 | [203] |
| *N. karatau* | S | Pa&Pr |  |  |  |  |  |  | -2.375 | 0.08 | 1.456 | 0.028 | 0.95 | [183] |
| *N. malleata* | S | Pa&Pr | 115747 | 133 | 194 | 2 | 27523 |  | -2.375 | 0.08 | 1.456 | 0.028 | 0.95 | [203] |
| *N. perbella* | S | Pa&Pr | 226642 | 692 | 722 | 3 | 9193 | 2971 | -2.375 | 0.08 | 1.456 | 0.028 | 0.95 | [202] |
| *N. retenta* | S | Pa&Pr | 317340 | 59 | 146 | 2 | 20018 |  | -2.375 | 0.08 | 1.456 | 0.028 | 0.95 | [203] |
| *N. robusta* | S | Pa&Pr | 306324 | 51 | 135 | 2 | 30523 | 16367 | -2.375 | 0.08 | 1.456 | 0.028 | 0.95 | [203] |
| Pamphiliidae  *Scabolyda*  *S. orientalis* | S | Ph |  |  |  |  |  |  | -2.375 | 0.08 | 1.456 | 0.028 | 0.95 | [204] |
| Pelecinidae  *Archaeopelecinus*  *A. jinzhouensis* | S | Pa&Pr | 607943 | 413 | 443 | 2 | 4266 |  | -2.375 | 0.08 | 1.456 | 0.028 | 0.95 | [205] |
| *A. tebbei* | S | Pa&Pr | 157140 | 186 | 230 | 3 | 4577 |  | -2.375 | 0.08 | 1.456 | 0.028 | 0.95 | [205] |
| *Cathaypelecinus*  *C. daohugouensis* | S | Pa&Pr | 609920 | 143 | 185 | 2 | 2951 |  | -2.375 | 0.08 | 1.456 | 0.028 | 0.95 | [205] |
| Praeaulacidae  *Archaulacus*  *A. probus* | S | Pa&Pr | 340087 | 279 | 360 | 1 | 24526 |  | -2.375 | 0.08 | 1.456 | 0.028 | 0.95 | [206] |
| *Aulacogastrinus*  *A. hebeiensis* | S | Pa&Pr |  |  |  |  |  |  | -2.375 | 0.08 | 1.456 | 0.028 | 0.95 | [183] |
| *A. insculptus* | S | Pa&Pr |  |  |  |  |  |  | -2.375 | 0.08 | 1.456 | 0.028 | 0.95 | [183] |
| *A. longaciculatus* | S | Pa&Pr |  |  |  |  |  |  | -2.375 | 0.08 | 1.456 | 0.028 | 0.95 | [183] |
| *Eosaulacus*  *E. giganteus* | S | Pa&Pr |  |  |  |  |  |  | -2.375 | 0.08 | 1.456 | 0.028 | 0.95 | [186] |
| *E. granulatus* | S | Pa&Pr |  |  |  |  |  |  | -2.375 | 0.08 | 1.456 | 0.028 | 0.95 | [183] |
| *Praeaulacon*  *P. elegantulus* | S | Pa&Pr |  |  |  |  |  |  | -2.375 | 0.08 | 1.456 | 0.028 | 0.95 | [183] |
| *P. ningchengensis* | S | Pa&Pr |  |  |  |  |  |  | -2.375 | 0.08 | 1.456 | 0.028 | 0.95 | [183] |
| *Praeaulacus*  *P. afflatus* | S | Pa&Pr |  |  |  |  |  |  | -2.375 | 0.08 | 1.456 | 0.028 | 0.95 | [183] |
| *P. byssinus* | S | Pa&Pr |  |  |  |  |  |  | -2.375 | 0.08 | 1.456 | 0.028 | 0.95 | [192] |
| *P. daohugouensis* | S | Pa&Pr |  |  |  |  |  |  | -2.375 | 0.08 | 1.456 | 0.028 | 0.95 | [183] |
| *P. exquisitus* | S | Pa&Pr |  |  |  |  |  |  | -2.375 | 0.08 | 1.456 | 0.028 | 0.95 | [183] |
| *P. obtutus* | S | Pa&Pr | 325992 | 54 | 96 | 1 | 15343 |  | -2.375 | 0.08 | 1.456 | 0.028 | 0.95 | 207 |
| *P. orientalis* | S | Pa&Pr |  |  |  |  |  |  | -2.375 | 0.08 | 1.456 | 0.028 | 0.95 | [183] |
| *P. rectus* | S | Pa&Pr | 526746 | 563 | 595 | 1 | 23560 | 8207 | -2.375 | 0.08 | 1.456 | 0.028 | 0.95 | [207] |
| *P. robustus* | S | Pa&Pr |  |  |  |  |  |  | -2.375 | 0.08 | 1.456 | 0.028 | 0.95 | [183] |
| *P. scabratus* | S | Pa&Pr |  |  |  |  |  |  | -2.375 | 0.08 | 1.456 | 0.028 | 0.95 | [183] |
| *P. sculptus* | S | Pa&Pr |  |  |  |  |  |  | -2.375 | 0.08 | 1.456 | 0.028 | 0.95 | [183] |
| *P. subrhombeus* | S | Pa&Pr | 301576 | 74 | 103 | 1 | 12548 | 9883 | -2.375 | 0.08 | 1.456 | 0.028 | 0.95 | [206] |
| *P. tenellus* | S | Pa&Pr | 254800 | 274 | 297 | 1 | 9456 |  | -2.375 | 0.08 | 1.456 | 0.028 | 0.95 | [206] |
| *Sinaulacogastrinus*  *S. eucallus* | S | Pa&Pr |  |  |  |  |  |  | -2.375 | 0.08 | 1.456 | 0.028 | 0.95 | [183] |
| *S. solidus* | S | Pa&Pr | 106560 | 64 | 253 | 5 | 14348 | 9588 | -2.375 | 0.08 | 1.456 | 0.028 | 0.95 | [186] |
| *Sinevania*  *S. speciosa* | S | Pa&Pr |  |  |  |  |  |  | -2.375 | 0.08 | 1.456 | 0.028 | 0.95 | [186] |
| Xyelidae  *Abrotoxyela*  *A. lepida* | S | Ph | 358940 | 536 | 593 | 2 | 48129 |  | -2.375 | 0.08 | 1.456 | 0.028 | 0.95 | [208] |
| *A. multiciliata* | S | Ph | 383080 | 475 | 555 | 2 | 75645 |  | -2.375 | 0.08 | 1.456 | 0.028 | 0.95 | [208] |
| *Aequixyela*  *A. immensa* | S | Ph | 278300 | 552 | 581 | 1 | 6144 |  | -2.375 | 0.08 | 1.456 | 0.028 | 0.95 | [209] |
| *Cathayxyela*  *C. extensa* | S | Ph | 339309 | 281 | 305 | 2 | 9780 |  | -2.375 | 0.08 | 1.456 | 0.028 | 0.95 | [209] |
| *Platyxyela*  *P. unica* | S | Ph | 266700 | 722 | 742 | 2 | 7655 | 8614 | -2.375 | 0.08 | 1.456 | 0.028 | 0.95 | [210] |
| Xyelotomidae  *Abrotoma*  *A. robusta* | S | Ph | 331128 | 673 | 708 | 2 | 20775 |  | -2.375 | 0.08 | 1.456 | 0.028 | 0.95 | [211] |
| *Aethotoma*  *A. aninomorpha* | S | Ph | 393966 | 534 | 564 | 2 | 9246 |  | -2.375 | 0.08 | 1.456 | 0.028 | 0.95 | [212] |
| *Paradoxotoma*  *P. tsaiae* | S | Ph | 308856 | 300 | 359 | 2 | 17290 |  | -2.375 | 0.08 | 1.456 | 0.028 | 0.95 | [211] |
| *Synaptotoma*  *S. limi* | S | Ph | 254045 | 639 | 679 | 2 | 14280 |  | -2.375 | 0.08 | 1.456 | 0.028 | 0.95 | [211] |
| *Xyelocerus*  *X. diaphanus* | S | Ph | 282734 | 637 | 667 | 2 | 18855 |  | -2.375 | 0.08 | 1.456 | 0.028 | 0.95 | [211] |
| *Xyelotoma*  *X. macroclada* | S | Ph | 323334 | 668 | 712 | 2 | 17717 | 9203 | -2.375 | 0.08 | 1.456 | 0.028 | 0.95 | [211] |
| Xyelydidae  *Brevilyda*  *B. provecta* | S | Ph | 374814 | 537 | 567 | 3 | 5440 |  | -2.375 | 0.08 | 1.456 | 0.028 | 0.95 | [213] |
| *Ferganolyda*  *F. charybdis* | S | Ph | 386400 | 173 | 244 | 5 | 33170 | 16335 | -2.375 | 0.08 | 1.456 | 0.028 | 0.95 | [214] |
| *F. chungkuei* | S | Ph | 236360 | 288 | 377 | 10 | 19163 |  | -2.375 | 0.08 | 1.456 | 0.028 | 0.95 | [214] |
| *F. eucalla* | S | Ph |  |  |  |  |  |  | -2.375 | 0.08 | 1.456 | 0.028 | 0.95 | [215] |
| *F. insolita* | S | Ph |  |  |  |  |  |  | -2.375 | 0.08 | 1.456 | 0.028 | 0.95 | [215] |
| *F. scylla* | S | Ph | 102312 | 179 | 276 | 5 | 39673 |  | -2.375 | 0.08 | 1.456 | 0.028 | 0.95 | [214] |
| *Medilyda*  *M. distorta* | S | Ph | 245660 | 312 | 336 | 2 | 2866 |  | -2.375 | 0.08 | 1.456 | 0.028 | 0.95 | [213] |
| *M. procera* | S | Ph | 268800 | 555 | 577 | 2 | 7359 |  | -2.375 | 0.08 | 1.456 | 0.028 | 0.95 | [213] |
| *Strenolyda*  *S. marginalis* | S | Ph | 314699 | 314 | 339 | 3 | 5510 |  | -2.375 | 0.08 | 1.456 | 0.028 | 0.95 | [213] |
| *S. retrorsa* | S | Ph | 188916 | 272 | 312 | 2 | 8811 |  | -2.375 | 0.08 | 1.456 | 0.028 | 0.95 | [213] |
| **Lepidoptera**  Ascololepidopterigidae  *Ascololepidopterix*  *A. multinerve* | S | Ph | 288840 | 57 | 126 | 2 | 13241 | 24280 | -3.138 | 0.129 | 1.483 | 0.031 | 0.96 | [216] |
| *Pegolepidopteron*  *P. latiala* | S | Ph | 98256 | 24 | 50 | 1 | 5127 | 9426 | -3.138 | 0.129 | 1.483 | 0.031 | 0.96 | [216] |
| *Trionolepidopteron*  *T. admarginis* | S | Ph | 304500 | 36 | 117 | 1 | 24279 | 35644 | -3.138 | 0.129 | 1.483 | 0.031 | 0.96 | [216] |
| Eolepidopterigidae  *Aclemus*  *A. patulus* | S | Ph | 156122 | 250 | 282 | 1 | 6660 | 6224 | -3.138 | 0.129 | 1.483 | 0.031 | 0.96 | [217] |
| *Akainalepidopteron*  *A. elachipteron* | S | Ph | 342045 | 58 | 145 | 2 | 20840 | 35269 | -3.138 | 0.129 | 1.483 | 0.031 | 0.96 | [216] |
| *Dynamilepidopteron*  *D. aspinosus* | S | Ph | 233025 | 356 | 425 | 2 | 11510 | 13203 | -3.138 | 0.129 | 1.483 | 0.031 | 0.96 | [216] |
| *Grammikolepidopteron*  *G. extensus* | S | Ph | 114218 | 47 | 92 | 1 | 5196 | 6381 | -3.138 | 0.129 | 1.483 | 0.031 | 0.96 | [216] |
| *Longcapitalis*  *L. excelsus* | S | Ph | 273042 | 58 | 140 | 2 | 13703 | 26191 | -3.138 | 0.129 | 1.483 | 0.031 | 0.96 | [217] |
| *Petilicorpus*  *P. cristatus* | S | Ph | 309657 | 260 | 386 | 2 | 12951 |  | -3.138 | 0.129 | 1.483 | 0.031 | 0.96 | [216] |
| *Quadruplecivena*  *Q. celsa* | S | Ph | 240416 | 450 | 499 | 2 | 9388 | 11727 | -3.138 | 0.129 | 1.483 | 0.031 | 0.96 | [216] |
| *Seresilepidopteron*  *S. dualis* | S | Ph | 152218 | 43 | 84 | 1 | 3860 | 8411 | -3.138 | 0.129 | 1.483 | 0.031 | 0.96 | [216] |
| Mesokristenseniidae  *Kladolepidopteron*  *K. oviformis* | S | Ph | 356384 | 370 | 412 | 1 | 7296 | 9320 | -3.138 | 0.129 | 1.483 | 0.031 | 0.96 | [216] |
| *K. parva* | S | Ph | 236980 | 566 | 620 | 1 | 11651 | 12421 | -3.138 | 0.129 | 1.483 | 0.031 | 0.96 | [216] |
| *K. subaequalis* | S | Ph | 349776 | 50 | 111 | 1 |  | 23069 | -3.138 | 0.129 | 1.483 | 0.031 | 0.96 | [216] |
| *Mesokristensenia*  *M. angustipenna* | S | Ph | 99224 | 204 | 263 | 1 |  | 18418 | -3.138 | 0.129 | 1.483 | 0.031 | 0.96 | [218] |
| *M. latipenna* | S | Ph | 124872 | 30 | 91 | 1 |  | 22291 | -3.138 | 0.129 | 1.483 | 0.031 | 0.96 | [218] |
| *M. sinica* | S | Ph | 103874 | 252 | 298 | 1 |  | 18960 | -3.138 | 0.129 | 1.483 | 0.031 | 0.96 | [218] |
| *M. trichophora* | S | Ph | 298592 | 42 | 112 | 1 | 25176 | 26442 | -3.138 | 0.129 | 1.483 | 0.031 | 0.96 | [216] |
| **Mantophasmatodea**  Mantophasmatidae  *Juramantophasma*  *J. sinica* | S | Ph | 216820 | 113 | 206 | 10 | 18307 |  | -2.14 | 0.036 | 1.331 | 0.011 | 0.94 | [219] |
| **Mecoptera**  Incertae sedis  *Fortiholcorpa*  *F. paradoxa* | S | Pr | 375154 | 16 | 47 | 5 | 4476 |  | -1.801 | 0.758 | 1.219 | 0.254 | 0.76 | [220] |
| *Miriholcorpa*  *M. forcipata* | S | Pr | 372232 | 352 | 373 | 2 | 5852 | 7284 | -1.801 | 0.758 | 1.219 | 0.254 | 0.76 | [220] |
| Bittacidae  *Composibittacus*  *C. bipunctatus* | E&S | Pr | 338373 | 9 | 26 | 1 |  | 29644 | -1.801 | 0.758 | 1.219 | 0.254 | 0.76 | [221] |
| *C. reticulatus* | E&S | Pr | 285904 | 13 | 28 | 1 |  | 21052 | -1.801 | 0.758 | 1.219 | 0.254 | 0.76 | [221] |
| *Decoribittacus*  *D. euneurus* | E&S | Pr | 102992 | 121 | 171 | 5 | 8491 | 10381 | -1.801 | 0.758 | 1.219 | 0.254 | 0.76 | [222] |
| *D. stictus* | E&S | Pr | 180540 | 123 | 218 | 5 |  | 40180 | -1.801 | 0.758 | 1.219 | 0.254 | 0.76 | [222] |
| *Exilibittacus*  *E. foliaceus* | E&S | Pr | 436840 | 599 | 643 | 3 | 3931 | 4340 | -1.801 | 0.758 | 1.219 | 0.254 | 0.76 | [223] |
| *E. lii* | E&S | Pr | 87009 | 57 | 92 | 2 | 7329 | 3104 | -1.801 | 0.758 | 1.219 | 0.254 | 0.76 | [224] |
| *E. plagioneurus* | E&S | Pr | 464607 | 17 | 75 | 3 | 6321 |  | -1.801 | 0.758 | 1.219 | 0.254 | 0.76 | [223] |
| *Formosibittacus*  *F. macularis* | E&S | Pr | 316030 | 60 | 141 | 5 | 9156 | 12336 | -1.801 | 0.758 | 1.219 | 0.254 | 0.76 | [225] |
| *Jurahylobittacus*  *J. astictus* | E&S | Pr | 286965 | 39 | 137 | 3.5 | 9692 | 10008 | -1.801 | 0.758 | 1.219 | 0.254 | 0.76 | [225] |
| *Karattacus*  *K. longialatus* | E&S | Pr | 449480 | 167 | 214 | 3 | 11674 | 23233 | -1.801 | 0.758 | 1.219 | 0.254 | 0.76 | [222] |
| *Mongolbittacus*  *M. daohugouensis* | E&S | Pr | 436078 | 546 | 589 | 2 | 11888 | 21734 | -1.801 | 0.758 | 1.219 | 0.254 | 0.76 | [226] |
| *M. oligophlebius* | E&S | Pr | 404055 | 30 | 95 | 2 | 31449 | 20104 | -1.801 | 0.758 | 1.219 | 0.254 | 0.76 | [226] |
| *M. speciosus* | E&S | Pr | 356940 | 339 | 384 | 3 | 2564 | 4682 | -1.801 | 0.758 | 1.219 | 0.254 | 0.76 | [223] |
| *Orthobittacus*  *O. maculosus* | E&S | Pr | 342540 | 263 | 277 | 1 | 2544 | 3739 | -1.801 | 0.758 | 1.219 | 0.254 | 0.76 | [221] |
| *O. suni* | E&S | Pr | 253638 | 119 | 181 | 5 | 13816 | 22719 | -1.801 | 0.758 | 1.219 | 0.254 | 0.76 | [227] |
| *Preanabittacus*  *P. validus* | E&S | Pr | 736386 | 126 | 202 | 4 | 35749 | 25438 | -1.801 | 0.758 | 1.219 | 0.254 | 0.76 | [228] |
| Choristopsychidae  *Choristopsyche*  *C. asticta* | S | Pr | 171021 | 216 | 228 | 2 | 2449 | 6839 | -1.801 | 0.758 | 1.219 | 0.254 | 0.76 | [229] |
| *C. perfecta* | S | Pr | 356553 | 186 | 229 | 5 | 4016 | 9199 | -1.801 | 0.758 | 1.219 | 0.254 | 0.76 | [229] |
| *C. tenuinervis* | S | Pr |  |  |  |  |  |  | -1.801 | 0.758 | 1.219 | 0.254 | 0.76 | [229] |
| *Paristopsyche*  *P. angelineae* | S | Pr | 341250 | 433 | 499 | 5 | 5344 | 10038 | -1.801 | 0.758 | 1.219 | 0.254 | 0.76 | [229] |
| Cimbrophlebiidae  *Bellicimbrophlebia*  *B. angusta* | S | Pr | 128169 | 230 | 256 | 5 | 6739 | 6135 | -1.801 | 0.758 | 1.219 | 0.254 | 0.76 | [230] |
| *B. cruciata* | S | Pr | 174096 | 18 | 53 | 5 |  | 15776 | -1.801 | 0.758 | 1.219 | 0.254 | 0.76 | [230] |
| *B. disvena* | S | Pr | 212065 | 241 | 277 | 5 | 7980 | 11352 | -1.801 | 0.758 | 1.219 | 0.254 | 0.76 | [230] |
| *B. eumorpha* | S | Pr | 182726 | 40 | 78 | 5 | 5721 | 8702 | -1.801 | 0.758 | 1.219 | 0.254 | 0.76 | [230] |
| *B. heteroneura* | S | Pr | 165600 | 501 | 570 | 5 |  | 52408 | -1.801 | 0.758 | 1.219 | 0.254 | 0.76 | [231] |
| *Cimbrophlebia*  *C. amoena* | S | Pr | 297018 | 240 | 276 | 5 | 5374 | 5839 | -1.801 | 0.758 | 1.219 | 0.254 | 0.76 | [231] |
| *C. gracilenta* | S | Pr | 71336 | 245 | 281 | 5 | 6763 | 6008 | -1.801 | 0.758 | 1.219 | 0.254 | 0.76 | [231] |
| *Juracimbrophlebia*  *J. ginkgofolia* | S | Pr | 326610 | 261 | 300 | 5 | 8341 | 12174 | -1.801 | 0.758 | 1.219 | 0.254 | 0.76 | [232] |
| *Mirorcimbrophlebia*  *M. daohugouensis* | S | Pr |  |  |  |  |  |  | -1.801 | 0.758 | 1.219 | 0.254 | 0.76 | [230] |
| *Perfecticimbrophlebia*  *P. laetus* | S | Pr | 168500 | 79 | 113 | 5 | 8016 | 6977 | -1.801 | 0.758 | 1.219 | 0.254 | 0.76 | [228] |
| *Telobittacus*  *T. bellus* | S | Pr | 289000 | 156 | 181 | 5 | 5145 |  | -1.801 | 0.758 | 1.219 | 0.254 | 0.76 | [230] |
| *T. decorus* | S | Pr | 174420 | 533 | 605 | 5 |  | 59200 | -1.801 | 0.758 | 1.219 | 0.254 | 0.76 | [231] |
| Eomeropidae  *Jurathauma*  *J. simplex* | E&S | Sa | 493661 | 351 | 421 | 4 | 18813 | 22659 | -1.801 | 0.758 | 1.219 | 0.254 | 0.76 | [233] |
| *Tsuchingothauma*  *T. shihi* | E&S | Sa | 182972 | 78 | 134 | 5 | 10063 | 22771 | -1.801 | 0.758 | 1.219 | 0.254 | 0.76 | [234] |
| Holcorpidae  *Conicholcorpa*  *C. stigmosa* | S | Pr | 540132 | 285 | 320 | 2 | 16761 | 14656 | -1.801 | 0.758 | 1.219 | 0.254 | 0.76 | [235] |
| Mesopsychidae  *Epicharmesopsyche*  *E. pentavenulosa* | E&S | Ph | 511864 | 36 | 91 | 5 | 7305 | 18364 | -1.801 | 0.758 | 1.219 | 0.254 | 0.76 | [236] |
| *Lichnomesopsyche*  *L. daohugouensis* | E&S | Ph |  |  |  |  |  |  | -1.801 | 0.758 | 1.219 | 0.254 | 0.76 | [229] |
| *L. gloriae* | E&S | Ph | 139920 | 11 | 104 | 10 | 7682 | 16995 | -1.801 | 0.758 | 1.219 | 0.254 | 0.76 | [237] |
| *L. prochorista* | E&S | Ph | 507650 | 26 | 59 | 5 | 6006 | 7661 | -1.801 | 0.758 | 1.219 | 0.254 | 0.76 | [238] |
| *Vitimopsyche*  *V. pristina* | E&S | Ph | 577100 | 513 | 561 | 5 | 4872 | 7184 | -1.801 | 0.758 | 1.219 | 0.254 | 0.76 | [238] |
| Nannochoristidae  *Itaphlebia*  *I. amoena* | H&S | Pr | 186984 | 1 | 39 | 1 | 19751 | 25770 | -1.801 | 0.758 | 1.219 | 0.254 | 0.76 | [239] |
| *I. decorosa* | H&S | Pr | 155610 | 168 | 208 | 1 |  | 24904 | -1.801 | 0.758 | 1.219 | 0.254 | 0.76 | [239] |
| *I. exquisita* | H&S | Pr | 101088 | 173 | 205 | 2 | 3994 | 6156 | -1.801 | 0.758 | 1.219 | 0.254 | 0.76 | [240] |
| *I. jeniseica* | H&S | Pr | 293216 | 367 | 440 | 2 |  | 28003 | -1.801 | 0.758 | 1.219 | 0.254 | 0.76 | [239] |
| *I. laeta* | H&S | Pr | 398463 | 104 | 174 | 2 | 10703 | 19680 | -1.801 | 0.758 | 1.219 | 0.254 | 0.76 | [240] |
| *I. longiovata* | H&S | Pr | 160380 | 410 | 442 | 1 | 4994 | 1269 | -1.801 | 0.758 | 1.219 | 0.254 | 0.76 | [239] |
| *I. multa* | H&S | Pr | 391251 | 40 | 78 | 1 |  | 22950 | -1.801 | 0.758 | 1.219 | 0.254 | 0.76 | [239] |
| *I. ruderalis* | H&S | Pr |  |  |  |  |  |  | -1.801 | 0.758 | 1.219 | 0.254 | 0.76 | [239] |
| Orthophlebiidae  *Gigaphlebia*  *G. riccardii* | E&S | Sa&Om | 422928 | 319 | 358 | 3 | 60631 | 58840 | -1.801 | 0.758 | 1.219 | 0.254 | 0.76 | [241] |
| *Longiphlebia*  *L. stigmosa* | E&S | Sa&Om | 450826 | 495 | 536 | 5 |  | 14513 | -1.801 | 0.758 | 1.219 | 0.254 | 0.76 | [242] |
| *Orthophlebia*  *O. (Dolichophlebia) xiaofangzhangziensis* | E&S | Sa&Om |  |  |  |  |  |  | -1.801 | 0.758 | 1.219 | 0.254 | 0.76 | [16] |
| *O. nervulosa* | E&S | Sa&Om | 365971 | 40 | 76 | 5 |  | 8377 | -1.801 | 0.758 | 1.219 | 0.254 | 0.76 | [243] |
| Panorpidae  *Jurassipanorpa*  *J. impunctata* | S | Sa | 369334 | 14 | 38 | 1 | 9720 |  | -1.801 | 0.758 | 1.219 | 0.254 | 0.76 | [244] |
| *J. sticta* | S | Sa | 366628 | 13 | 40 | 1 |  | 18851 | -1.801 | 0.758 | 1.219 | 0.254 | 0.76 | [244] |
| Pseudopolycentropodidae  *Pseudopolycentropus*  *P. janeannae* | E&S | Ph |  |  |  |  |  |  | -1.801 | 0.758 | 1.219 | 0.254 | 0.76 | [245] |
| *P. novokshonovi* | E&S | Ph |  |  |  |  |  |  | -1.801 | 0.758 | 1.219 | 0.254 | 0.76 | [246] |
| *P. daohugouensis* | E&S | Ph |  |  |  |  |  |  | -1.801 | 0.758 | 1.219 | 0.254 | 0.76 | [245] |
| *Sinopolycentropus*  *S. rasnitsyni* | E&S | Ph | 473625 | 345 | 383 | 1 | 13774 |  | -1.801 | 0.758 | 1.219 | 0.254 | 0.76 | [247] |
| *Tsuchingothauma*  *T. shihi* | E&S | Ph |  |  |  |  |  |  | -1.801 | 0.758 | 1.219 | 0.254 | 0.76 | [234] |
| **Megaloptera**  Corydalidae  *Eochauliodes*  *E. striolatus* | H&S | Pr | 130548 | 23 | 92 | 5 | 19909 | 29268 | -2.14 | 0.036 | 1.331 | 0.011 | 0.94 | [248] |
| Jurochauliodes  J. ponomarenkoi | H&S | Pr | 190930 | 232 | 300 | 5 | 31402 |  | -2.14 | 0.036 | 1.331 | 0.011 | 0.94 | [249] |
| **Neuroptera**  Aetheogrammatidae  *Ectopogramma*  *E. kalligrammoides* | S | Pr |  |  |  |  |  |  | -1.684 | 0.146 | 1.172 | 0.033 | 0.98 | [250] |
| Berothidae  *Sinosmylites*  *S. fumosus* | H&S | Pr | 256530 | 24 | 101 | 1 |  | 69972 | -1.684 | 0.146 | 1.172 | 0.033 | 0.98 | [251] |
| *S. rasnitsyni* | H&S | Pr | 313296 | 414 | 482 | 2 | 6700 | 16195 | -1.684 | 0.146 | 1.172 | 0.033 | 0.98 | [251] |
| Chrysopidae  *Mesypochrysa*  *M. intermedia* | S | Pr | 298302 | 56 | 103 | 3 | 8658 | 18046 | -0.951 | 0.736 | 0.222 | 0.279 | 0.31 | [252] |
| *M. sinica* | S | Pr | 288408 | 388 | 475 | 4 | 11706 | 31442 | -0.951 | 0.736 | 0.222 | 0.279 | 0.31 | [252] |
| Grammolingiidae  *Chorilingia*  *C. euryptera* | S | Pr | 117111 | 329 | 362 | 10 | 2429 | 10092 | -1.684 | 0.146 | 1.172 | 0.033 | 0.98 | [253] |
| *C. parvica* | S | Pr | 84316 | 271 | 371 | 10 |  | 32278 | -1.684 | 0.146 | 1.172 | 0.033 | 0.98 | [253] |
| *C. peregrina* | S | Pr | 77250 | 264 | 359 | 10 |  | 34362 | -1.684 | 0.146 | 1.172 | 0.033 | 0.98 | [253] |
| *C. translucida* | S | Pr | 458044 | 557 | 680 | 10 |  | 93987 | -1.684 | 0.146 | 1.172 | 0.033 | 0.98 | [253] |
| *Grammolingia*  *G. binervis* | S | Pr | 161231 | 381 | 483 | 10 |  | 57699 | -1.684 | 0.146 | 1.172 | 0.033 | 0.98 | [254] |
| *G. boi* | S | Pr | 390145 | Eosesatheris | 686 |  |  | 31373 | -1.684 | 0.146 | 1.172 | 0.033 | 0.98 | [255] |
| *G. sticta* | S | Pr | 152725 | 405 | 485 | 10 |  | 54482 | -1.684 | 0.146 | 1.172 | 0.033 | 0.98 | [254] |
| *G. uniserialis* | S | Pr | 271364 | 411 | 494 | 10 |  | 19614 | -1.684 | 0.146 | 1.172 | 0.033 | 0.98 | [254] |
| *Leptolingia*  *L. calonervis* | S | Pr | 255651 | 599 | 659 | 10 | 2928 | 21939 | -1.684 | 0.146 | 1.172 | 0.033 | 0.98 | [256] |
| *L. imminuta* | S | Pr | 316834 | 511 | 610 | 5 |  | 101446 | -1.684 | 0.146 | 1.172 | 0.033 | 0.98 | [257] |
| *L. jurassica* | S | Pr | 425430 | Eosesatheris | 1588 |  |  | 31023 | -1.684 | 0.146 | 1.172 | 0.033 | 0.98 | [255] |
| *L. tianyiensis* | S | Pr | 333802 | Eosesatheris | 1832 |  |  | 44506 | -1.684 | 0.146 | 1.172 | 0.033 | 0.98 | [255] |
| *Litholingia*  *L. eumorpha* | S | Pr | 315119 | Eosesatheris | 2841 |  |  | 139608 | -1.684 | 0.146 | 1.172 | 0.033 | 0.98 | [255] |
| *L. polychotoma* | S | Pr | 323429 | Eosesatheris | 1356 |  |  | 50711 | -1.684 | 0.146 | 1.172 | 0.033 | 0.98 | [255] |
| *L. ptesa* | S | Pr | 150274 | 257 | 323 | 10 | 2715 | 24784 | -1.684 | 0.146 | 1.172 | 0.033 | 0.98 | [256] |
| *L. rhora* | S | Pr | 248624 | Eosesatheris | 2317 |  |  | 115181 | -1.684 | 0.146 | 1.172 | 0.033 | 0.98 | [252] |
| Ithonidae  *Guithone*  *G. bethouxi* | S | Pr | 227766 | 122 | 192 | 5 | 11153 |  | -1.684 | 0.146 | 1.172 | 0.033 | 0.98 | [258] |
| Kalligrammatidae  *Affinigramma*  *A. myrioneura* | S | Pr | 353304 | 285 | 317 | 10 |  | 17444 | -1.684 | 0.146 | 1.172 | 0.033 | 0.98 | [259] |
| *Apochrysogramma*  *A. rotundum* | S | Pr | 407690 | 71 | 213 | 10 |  | 262303 | -1.684 | 0.146 | 1.172 | 0.033 | 0.98 | [260] |
| *Huiyingogramma*  *H. formosum* | S | Pr | 566800 | 467 | 607 | 20 |  | 111961 | -1.684 | 0.146 | 1.172 | 0.033 | 0.98 | [261] |
| *Kalligramma*  *K. albifasciatum* | S | Pr | 191544 | 127 | 201 | 10 |  | 79038 | -1.684 | 0.146 | 1.172 | 0.033 | 0.98 | [262] |
| *K. brachyrhyncha* | S | Pr | 366263 | 520 | 574 | 10 |  | 52271 | -1.684 | 0.146 | 1.172 | 0.033 | 0.98 | [259] |
| *K. circularia* | S | Pr | 268128 | 286 | 323 | 10 |  | 22420 | -1.684 | 0.146 | 1.172 | 0.033 | 0.98 | [259] |
| *K. delicatum* | S | Pr | 606958 | 292 | 593 | 10 |  | 115502 | -1.684 | 0.146 | 1.172 | 0.033 | 0.98 | [263] |
| *K. elegans* | S | Pr | 181720 | 95 | 156 | 10 |  | 102157 | -1.684 | 0.146 | 1.172 | 0.033 | 0.98 | [262] |
| *K. paradoxum* | S | Pr | 489464 | 487 | 614 | 20 |  | 103328 | -1.684 | 0.146 | 1.172 | 0.033 | 0.98 | [261] |
| *Kalligrammula*  *K. hani* | S | Pr | 329688 | 546 | 597 | 10 |  | 22340 | -1.684 | 0.146 | 1.172 | 0.033 | 0.98 | [264] |
| *K. lata* | S | Pr | 394752 | 549 | 706 | 20 |  | 145000 | -1.684 | 0.146 | 1.172 | 0.033 | 0.98 | [265] |
| *K. mongolica* | S | Pr | 357707 | 327 | 424 | 10 |  | 31438 | -1.684 | 0.146 | 1.172 | 0.033 | 0.98 | [264] |
| *Kallihemerobius*  *K. aciedentatus* | S | Pr | 267344 | 309 | 331 | 10 |  | 1724 | -1.684 | 0.146 | 1.172 | 0.033 | 0.98 | [259] |
| *K. almacellus* | S | Pr | 380016 | 491 | 581 | 10 |  | 59842 | -1.684 | 0.146 | 1.172 | 0.033 | 0.98 | [259] |
| *K. feroculus* | S | Pr | 358845 | 503 | 590 | 10 |  | 60146 | -1.684 | 0.146 | 1.172 | 0.033 | 0.98 | [259] |
| *K. pleioneurus* | S | Pr |  |  |  |  |  |  | -1.684 | 0.146 | 1.172 | 0.033 | 0.98 | [266] |
| *Protokalligramma*  *P. bifasciatum* | S | Pr | 356535 | 55 | 159 | 10 |  | 216007 | -1.684 | 0.146 | 1.172 | 0.033 | 0.98 | [260] |
| *Sinokalligramma*  *S. jurassicum* | S | Pr | 261120 | 127 | 198 | 10 |  | 102989 | -1.684 | 0.146 | 1.172 | 0.033 | 0.98 | [267] |
| *Stelligramma*  *S. allochroma* | S | Pr | 275406 | 553 | 594 | 10 |  | 77785 | -1.684 | 0.146 | 1.172 | 0.033 | 0.98 | [259] |
| Mantispidae  *Clavifemora*  *C. rotundata* | S | Pa&Pr | 139278 | 160 | 220 | 5 | 11848 |  | -1.684 | 0.146 | 1.172 | 0.033 | 0.98 | [268] |
| Mesochrysopidae  *Protoaristenymphes*  *P. daohugouensis* | S | Pr |  |  |  |  |  |  | -1.684 | 0.146 | 1.172 | 0.033 | 0.98 | [269] |
| Nymphidae  *Daonymphes*  *D. bisulca* | S | Pr | 170229 | 37 | 209 | 10 |  | 79680 | -1.684 | 0.146 | 1.172 | 0.033 | 0.98 | [270] |
| *Liminympha*  *L. makarkini* | S | Pr | 270227 | 495 | 583 | 5 | 11719 | 44098 | -1.684 | 0.146 | 1.172 | 0.033 | 0.98 | [271] |
| *Nymphites*  *N. bimaculatus* | S | Pr | 391866 | 6 | 191 | 10 |  | 11459 | -1.684 | 0.146 | 1.172 | 0.033 | 0.98 | [272] |
| *Osmylidae*  *Allotriosmylus*  *A. uniramosus* | H&S | Pr | 152600 | 265 | 292 | 1 |  | 21666 | -1.684 | 0.146 | 1.172 | 0.033 | 0.98 | [273] |
| *Arbusella*  *A. magna* | H&S | Pr | 169598 | 526 | 583 | 5 | 23475 | 36163 | -1.684 | 0.146 | 1.172 | 0.033 | 0.98 | [274] |
| *Archaeosmylidia*  *A. fusca* | H&S | Pr | 245810 | 20 | 183 | 5 |  | 69502 | -1.684 | 0.146 | 1.172 | 0.033 | 0.98 | [275] |
| *Enodinympha*  *E. imperfecta* | H&S | Pr | 192764 | 13 | 87 | 5 | 8887 | 16106 | -1.684 | 0.146 | 1.172 | 0.033 | 0.98 | [271] |
| *E. translucida* | H&S | Pr | 184621 | 277 | 382 | 10 | 4188 | 15815 | -1.684 | 0.146 | 1.172 | 0.033 | 0.98 | [271] |
| *Epiosmylus*  *E. panfilovi* | H&S | Pr | 202740 | 381 | 452 | 2 |  | 48122 | -1.684 | 0.146 | 1.172 | 0.033 | 0.98 | [276] |
| *Juraheterosmylus*  *J. antiquates* | H&S | Pr | 135900 | 48 | 106 | 5 | 5700 | 14296 | -1.684 | 0.146 | 1.172 | 0.033 | 0.98 | [277] |
| *J. astictus* | H&S | Pr | 149611 | 49 | 111 | 5 | 3290 | 13771 | -1.684 | 0.146 | 1.172 | 0.033 | 0.98 | [277] |
| *J. minor* | H&S | Pr | 176400 | 38 | 124 | 5 | 6558 | 17406 | -1.684 | 0.146 | 1.172 | 0.033 | 0.98 | [277] |
| *Jurakempynus*  *J. bellatulus* | H&S | Pr | 108072 | 231 | 333 | 10 |  | 28877 | -1.684 | 0.146 | 1.172 | 0.033 | 0.98 | [278] |
| *J. epunctatus* | H&S | Pr | 99034 | 204 | 332 | 10 |  | 25545 | -1.684 | 0.146 | 1.172 | 0.033 | 0.98 | [278] |
| *J. sinensis* | H&S | Pr | 103824 | 68 | 145 | 10 | 2581 | 16337 | -1.684 | 0.146 | 1.172 | 0.033 | 0.98 | [278] |
| *Nilionympha*  *N. pulchella* | H&S | Pr | 183928 | 122 | 206 | 10 | 2711 | 7771 | -1.684 | 0.146 | 1.172 | 0.033 | 0.98 | [271] |
| *N. shantouensis* | H&S | Pr | 122000 | 23 | 64 | 3 | 5789 | 16915 | -1.684 | 0.146 | 1.172 | 0.033 | 0.98 | [279] |
| *Palaeothyridosmylus*  *P. septemaculatus* | H&S | Pr | 241509 | 421 | 544 | 5 |  | 113549 | -1.684 | 0.146 | 1.172 | 0.033 | 0.98 | [280] |
| *Ponomarenkius*  *P. excellens* | H&S | Pr | 192427 | 254 | 311 | 5 |  | 23696 | -1.684 | 0.146 | 1.172 | 0.033 | 0.98 | [274] |
| *Tenuosmylus*  *T. brevineurus* | H&S | Pr | 125080 | 454 | 510 | 5 |  | 9036 | -1.684 | 0.146 | 1.172 | 0.033 | 0.98 | [281] |
| Osmylopsychopidae  *Daopsychops*  *D. bifasciatus* | S | Pr | 282285 | 559 | 657 | 5 |  | 138204 | -1.684 | 0.146 | 1.172 | 0.033 | 0.98 | [282] |
| *D. clausus* | S | Pr | 240524 | 552 | 639 | 5 |  | 107318 | -1.684 | 0.146 | 1.172 | 0.033 | 0.98 | [282] |
| *D. cubitalis* | S | Pr | 220110 | 365 | 428 | 5 |  | 62205 | -1.684 | 0.146 | 1.172 | 0.033 | 0.98 | [282] |
| *D. dissectus* | S | Pr | 257868 | 555 | 632 | 5 |  | 113533 | -1.684 | 0.146 | 1.172 | 0.033 | 0.98 | [282] |
| *D. inanis* | S | Pr | 286101 | 562 | 654 | 5 |  | 143912 | -1.684 | 0.146 | 1.172 | 0.033 | 0.98 | [282] |
| *Eupypsychops*  *E. confinis* | S | Pr | 291000 | 555 | 675 | 5 |  | 125156 | -1.684 | 0.146 | 1.172 | 0.033 | 0.98 | [282] |
| *E. ferox* | S | Pr | 359400 | 447 | 416 | 5 |  | 105641 | -1.684 | 0.146 | 1.172 | 0.033 | 0.98 | [282] |
| *Nematopsychops*  *N. unicus* | S | Pr | 332804 | 428 | 431 | 5 |  | 91576 | -1.684 | 0.146 | 1.172 | 0.033 | 0.98 | [282] |
| *Ochropsychops*  *O. multus* | S | Pr | 169120 | 430 | 516 | 5 |  | 75064 | -1.684 | 0.146 | 1.172 | 0.033 | 0.98 | [282] |
| *Stenopteropsychops*  *S. trifasciatus* | S | Pr | 151892 | 421 | 507 | 5 |  | 66520 | -1.684 | 0.146 | 1.172 | 0.033 | 0.98 | [282] |
| Panfiloviidae  *Epipanfilovia*  *E. oviformis* | S | Pr | 246753 | 47 | 100 | 10 |  | 52229 | -1.684 | 0.146 | 1.172 | 0.033 | 0.98 | [283] |
| Parakseneuridae  *Parakseneura*  *P. albadelta* | S | Pr | 289800 | 48 | 118 | 10 |  | 44139 | -1.684 | 0.146 | 1.172 | 0.033 | 0.98 | [284] |
| *P. albomacula* | S | Pr | 115928 | 25 | 76 | 10 |  | 38184 | -1.684 | 0.146 | 1.172 | 0.033 | 0.98 | [284] |
| *P. cavomaculata* | S | Pr | 382659 | 34 | 107 | 10 |  | 75564 | -1.684 | 0.146 | 1.172 | 0.033 | 0.98 | [284] |
| *P. curvivenis* | S | Pr | 182160 | 227 | 307 | 10 |  | 55117 | -1.684 | 0.146 | 1.172 | 0.033 | 0.98 | [284] |
| *P. directa* | S | Pr | 331890 | 42 | 113 | 10 |  | 73642 | -1.684 | 0.146 | 1.172 | 0.033 | 0.98 | [284] |
| *P. emarginata* | S | Pr | 390104 | 316 | 406 | 10 |  | 77599 | -1.684 | 0.146 | 1.172 | 0.033 | 0.98 | [284] |
| *P. inflata* | S | Pr | 191301 | 207 | 304 | 10 |  | 54067 | -1.684 | 0.146 | 1.172 | 0.033 | 0.98 | [284] |
| *P. metallica* | S | Pr | 200705 | 36 | 139 | 10 |  | 74954 | -1.684 | 0.146 | 1.172 | 0.033 | 0.98 | [284] |
| *P. nigrolinea* | S | Pr | 268830 | 46 | 143 | 10 |  | 69032 | -1.684 | 0.146 | 1.172 | 0.033 | 0.98 | [284] |
| *P. nigromacula* | S | Pr | 220725 | 49 | 149 | 10 |  | 101337 | -1.684 | 0.146 | 1.172 | 0.033 | 0.98 | [284] |
| *P. undula* | S | Pr | 160270 | 15 | 72 | 10 |  | 37893 | -1.684 | 0.146 | 1.172 | 0.033 | 0.98 | [284] |
| *Pseudorapisma*  *P. angustipenne* | S | Pr | 170400 | 41 | 104 | 10 |  | 32509 | -1.684 | 0.146 | 1.172 | 0.033 | 0.98 | [284] |
| *P. jurassicum* | S | Pr | 319676 | 41 | 133 | 10 |  | 77333 | -1.684 | 0.146 | 1.172 | 0.033 | 0.98 | [284] |
| *P. maculatum* | S | Pr | 302526 | 29 | 110 | 10 |  | 47144 | -1.684 | 0.146 | 1.172 | 0.033 | 0.98 | [284] |
| Polystoechotidae  *Jurapolystoechotes*  *J. melanolomus* | S | Pr | 171666 | 20 | 85 | 5 |  | 87992 | -1.684 | 0.146 | 1.172 | 0.033 | 0.98 | [285] |
| *Meilingius*  *M. giganteus* | S | Pr | 162060 | 24 | 67 | 5 |  | 70794 | -1.684 | 0.146 | 1.172 | 0.033 | 0.98 | [285] |
| Psychopsidae  *Cretapsychops*  *C. decipiens* | S | Pr | 333693 | 25 | 108 | 5 |  | 48851 | -1.684 | 0.146 | 1.172 | 0.033 | 0.98 | [286] |
| *Gigantopsychops*  *G. reticulatus* | S | Pr | 211323 | 453 | 509 | 5 |  | 122638 | -1.684 | 0.146 | 1.172 | 0.033 | 0.98 | [282] |
| Saucrosmylidae  *Bellinympha*  *B. dancei* | H&S | Pr | 34020 | 9 | 26 | 10 | 775 | 2196 | -1.684 | 0.146 | 1.172 | 0.033 | 0.98 | [287] |
| *B. filicifolia* | H&S | Pr | 38086 | 229 | 255 | 10 | 1019 | 4251 | -1.684 | 0.146 | 1.172 | 0.033 | 0.98 | [287] |
| *Daohugosmylus*  *D. castus* | H&S | Pr | 318828 | 387 | 508 | 20 |  | 59928 | -1.684 | 0.146 | 1.172 | 0.033 | 0.98 | [288] |
| *Huiyingosmylus*  *H. bellus* | H&S | Pr | 364496 | 350 | 462 | 20 |  | 60083 | -1.684 | 0.146 | 1.172 | 0.033 | 0.98 | [289] |
| *Laccosmylus*  *L. calophlebius* | H&S | Pr | 290540 | 53 | 141 | 10 |  | 133090 | -1.684 | 0.146 | 1.172 | 0.033 | 0.98 | [290] |
| *L. cicatricatus* | H&S | Pr | 396921 | 681 | 802 | 10 |  | 155138 | -1.684 | 0.146 | 1.172 | 0.033 | 0.98 | [291] |
| *L. latizonus* | H&S | Pr | 92664 | 27 | 93 | 10 |  | 48850 | -1.684 | 0.146 | 1.172 | 0.033 | 0.98 | [291] |
| *Rudiosmylus*  *R. ningchengensis* | H&S | Pr | 174106 | 59 | 105 | 5 |  | 89663 | -1.684 | 0.146 | 1.172 | 0.033 | 0.98 | [290] |
| *Saucrosmylus*  *S. sambneurus* | H&S | Pr | 363654 | 150 | 243 | 10 | 12273 | 61789 | -1.684 | 0.146 | 1.172 | 0.033 | 0.98 | [290] |
| *Ulrikezza*  *U. aspoeckae* | H&S | Pr | 305240 | 420 | 463 | 10 |  | 17620 | -1.684 | 0.146 | 1.172 | 0.033 | 0.98 | [292] |
| **Odonata**  Incertae sedis  *Sinocymatophlebiella*  *S. hasticercus* | H | Pr |  |  |  |  |  |  | -2.14 | 0.036 | 1.331 | 0.011 | 0.94 | [293] |
| Campterophlebiidae  *Amnifleckia*  *A. guttata* | H | Pr | 124080 | 283 | 331 | 10 | 4015 | 6145 | -2.14 | 0.036 | 1.331 | 0.011 | 0.94 | [294] |
| *A. splendida* | H | Pr | 193200 | 496 | 546 | 5 |  | 80947 | -2.14 | 0.036 | 1.331 | 0.011 | 0.94 | [294] |
| *Angustiphlebia*  *A. mirabilis* | H | Pr | 295488 | 76 | 109 | 10 |  | 10265 | -2.14 | 0.036 | 1.331 | 0.011 | 0.94 | [295] |
| *Ctenogampsophlebia*  *C. reni* | H | Pr | 174932 | 348 | 386 | 5 | 8181 | 11391 | -2.14 | 0.036 | 1.331 | 0.011 | 0.94 | [296] |
| *Hsiufua*  *H. chaoi* | H | Pr | 200906 | 68 | 119 | 10 |  | 32886 | -2.14 | 0.036 | 1.331 | 0.011 | 0.94 | [297] |
| *Karatawia*  *K. sinica* | H | Pr | 109430 | 236 | 284 | 10 | 5750 | 7830 | -2.14 | 0.036 | 1.331 | 0.011 | 0.94 | [298] |
| *Parabrunetia*  *P. celinea* | H | Pr | 337952 | 464 | 529 | 10 |  | 41678 | -2.14 | 0.036 | 1.331 | 0.011 | 0.94 | [294] |
| *Parazygokaratawia*  *P. azari* | H | Pr | 103459 | 404 | 561 | 10 |  | 26838 | -2.14 | 0.036 | 1.331 | 0.011 | 0.94 | [299] |
| *Qibinlina*  *Q. sinica* | H | Pr | 202560 | 493 | 528 | 5 |  | 40747 | -2.14 | 0.036 | 1.331 | 0.011 | 0.94 | [300] |
| *Sinokaratawia*  *S. daohugouica* | H | Pr | 305728 | 120 | 181 | 10 |  | 20239 | -2.14 | 0.036 | 1.331 | 0.011 | 0.94 | [301] |
| *S. gloriosa* | H | Pr | 163236 | 93 | 173 | 10 |  | 22042 | -2.14 | 0.036 | 1.331 | 0.011 | 0.94 | [301] |
| *S. magica* | H | Pr | 229152 | 357 | 429 | 10 |  | 19128 | -2.14 | 0.036 | 1.331 | 0.011 | 0.94 | [301] |
| *S. prokopi* | H | Pr | 243525 | 150 | 222 | 20 | 4541 | 12676 | -2.14 | 0.036 | 1.331 | 0.011 | 0.94 | [301] |
| *Zygokaratawia*  *Z. reni* | H | Pr | 123134 | 110 | 169 | 10 | 5009 | 6600 | -2.14 | 0.036 | 1.331 | 0.011 | 0.94 | [302] |
| *Z. incompleta* | H | Pr | 181137 | 379 | 476 | 10 | 11351 | 19098 | -2.14 | 0.036 | 1.331 | 0.011 | 0.94 | [299] |
| Daohugoulibellulidae  *Daohugoulibellula*  *D. lini* | H | Pr | 211992 | 424 | 500 | 5 |  | 48512 | -2.14 | 0.036 | 1.331 | 0.011 | 0.94 | [303] |
| Euthemistidae  *Sinoeuthemis*  *S. daohugouensis* | H | Pr | 193749 | 53 | 135 | 10 | 13911 | - | -2.14 | 0.036 | 1.331 | 0.011 | 0.94 | [304] |
| Juraheterophlebiidae  *Juraheterophlebia*  *J. cancellosa* | H | Pr | 254740 | 358 | 460 | 2 | 26626 | 25922 | -2.14 | 0.036 | 1.331 | 0.011 | 0.94 | [305] |
| *J. sinica* | H | Pr | 167328 | Eosesatheris | 725 |  | 4769 | 11035 | -2.14 | 0.036 | 1.331 | 0.011 | 0.94 | [306] |
| Juralibellulidae  *Juralibellula*  *J. ningchengensis* | H | Pr | 190384 | 152 | 231 | 5 |  | 81621 | -2.14 | 0.036 | 1.331 | 0.011 | 0.94 | [307] |
| Paracymatophlebiidae  *Linqibinia*  *L. panae* | H | Pr | 227728 | 419 | 472 | 5 |  | 64215 | -2.14 | 0.036 | 1.331 | 0.011 | 0.94 | [308] |
| *Sinacymatophlebia*  *S. mongolica* | H | Pr |  |  |  |  |  |  | -2.14 | 0.036 | 1.331 | 0.011 | 0.94 | [309] |
| **Orthoptera**  Haglidae  *Archaboilus*  *A. musicus* | S | Ph | 1023300 | 286 | 315 | 5 |  | 43056 | -2.14 | 0.036 | 1.331 | 0.011 | 0.94 | [310] |
| *Liassophyllum*  *L. caii* | S | Ph | 590133 | 54 | 102 | 10 | 19724 | 12986 | -2.14 | 0.036 | 1.331 | 0.011 | 0.94 | [311] |
| Locustopsidae  *Locustopsis*  *L. rhytofemoralis* | S | Ph | 539534 | 615 | 673 | 2 | 40725 | 36690 | -2.14 | 0.036 | 1.331 | 0.011 | 0.94 | [312] |
| Prophalangopsidae  *Aboilus*  *A. chinensis* | S | Ph | 234500 | 87 | 211 | 10 |  | 91743 | -2.14 | 0.036 | 1.331 | 0.011 | 0.94 | [313] |
| *A. cornutus* | S | Ph | 145329 | 488 | 564 | 5 |  | 56344 | -2.14 | 0.036 | 1.331 | 0.011 | 0.94 | [314] |
| *A. perbellus* | S | Ph | 487872 | 129 | 266 | 10 |  | 108903 | -2.14 | 0.036 | 1.331 | 0.011 | 0.94 | [315] |
| *A. stratosus* | S | Ph | 156348 | 69 | 152 | 5 |  | 48061 | -2.14 | 0.036 | 1.331 | 0.011 | 0.94 | [314] |
| *Allaboilus*  *A. dicrus* | S | Ph | 344844 | 118 | 184 | 5 |  | 142944 | -2.14 | 0.036 | 1.331 | 0.011 | 0.94 | [316] |
| *A. gigantus* | S | Ph | 579867 | 700 | 767 | 10 | 131154 |  | -2.14 | 0.036 | 1.331 | 0.011 | 0.94 | [317] |
| *A. robustus* | S | Ph | 279225 | 317 | 352 | 10 |  | 38252 | -2.14 | 0.036 | 1.331 | 0.011 | 0.94 | [318] |
| *Angustaboilus*  *A. fangianus* | S | Ph |  |  |  |  |  |  | -2.14 | 0.036 | 1.331 | 0.011 | 0.94 | [319] |
| *Ashangopsis*  *A. daohugouensis* | S | Ph | 399555 | 155 | 327 | 10 |  | 62065 | -2.14 | 0.036 | 1.331 | 0.011 | 0.94 | [320] |
| *Bacharaboilus*  *B. jurassicus* | S | Ph | 101311 | 51 | 108 | 5 | 38060 | 41597 | -2.14 | 0.036 | 1.331 | 0.011 | 0.94 | [314] |
| *B. lii* | S | Ph | 433794 | 609 | 690 | 10 |  | 42467 | -2.14 | 0.036 | 1.331 | 0.011 | 0.94 | [321] |
| *Circulaboilus*  *C. aureus* | S | Ph | 141220 | 64 | 120 | 5 |  | 53312 | -2.14 | 0.036 | 1.331 | 0.011 | 0.94 | [319] |
| *C. priscus* | S | Ph | 356694 | 538 | 617 | 10 |  | 102647 | -2.14 | 0.036 | 1.331 | 0.011 | 0.94 | [318] |
| *Novaboilus*  *N. multifurcatus* | S | Ph |  |  |  |  |  |  | -2.14 | 0.036 | 1.331 | 0.011 | 0.94 | [319] |
| *Protaboilus*  *P. amblus* | S | Ph | 283582 | 497 | 558 | 10 |  | 97861 | -2.14 | 0.036 | 1.331 | 0.011 | 0.94 | [316] |
| *P. lini* | S | Ph | 291332 | 384 | 481 | 5 |  | 102381 | -2.14 | 0.036 | 1.331 | 0.011 | 0.94 | [316] |
| *P. rudis* | S | Ph | 291842 | 343 | 441 | 5 |  | 92663 | -2.14 | 0.036 | 1.331 | 0.011 | 0.94 | [316] |
| *Pseudohagala*  *P. shihi* | S | Ph | 371126 | 89 | 160 | 5 |  | 59898 | -2.14 | 0.036 | 1.331 | 0.011 | 0.94 | [319] |
| *Scalpellaboilus*  *S. angustus* | S | Ph | 301720 | 540 | 596 | 10 |  | 26488 | -2.14 | 0.036 | 1.331 | 0.011 | 0.94 | [318] |
| *Sigmaboilus*  *S. calophlebius* | S | Ph | 165750 | 38 | 202 | 10 |  | 82262 | -2.14 | 0.036 | 1.331 | 0.011 | 0.94 | [322] |
| *S. fuscus* | S | Ph |  |  |  |  |  |  | -2.14 | 0.036 | 1.331 | 0.011 | 0.94 | [323] |
| *S. gorochovi* | S | Ph | 231628 | 29 | 205 | 10 |  | 101570 | -2.14 | 0.036 | 1.331 | 0.011 | 0.94 | [324] |
| *S. longus* | S | Ph | 213840 | 47 | 149 | 10 |  | 101582 | -2.14 | 0.036 | 1.331 | 0.011 | 0.94 | [324] |
| *S. peregrinus* | S | Ph |  |  |  |  |  |  | -2.14 | 0.036 | 1.331 | 0.011 | 0.94 | [318] |
| *S. sinensis* | S | Ph | 236196 | 26 | 201 | 10 |  | 108949 | -2.14 | 0.036 | 1.331 | 0.011 | 0.94 | [324] |
| **Permopsocida**  Archipsyllidae  *Archipsylla*  *A. sinica* | S | Ph | 189937 | 108 | 189 | 1 |  | 5235 | -2.14 | 0.036 | 1.331 | 0.011 | 0.94 | [325] |
| **Phasmatodea**  Susumaniidae  *Adjacivena*  *A. rasnitsyni* | S | Ph | 429970 | 40 | 115 | 10 | 16386 | 21458 | -2.14 | 0.036 | 1.331 | 0.011 | 0.94 | [326] |
| **Plecoptera**  Baleyopterygidae  *Aristoleuctra*  *A. yehae* | H&A | Pr | 359898 | 487 | 679 | 5 | 15856 | 36390 | -2.024 | 0.204 | 1.206 | 0.071 | 0.94 | [327] |
| Capniidae  *Dobbertiniopteryx*  *D. juracapnia* | H&A | Ph | 132858 | 449 | 578 | 2 | 25753 | 34516 | -2.024 | 0.204 | 1.206 | 0.071 | 0.94 | [328] |
| Notonemouridae  *Paranotonemoura*  *P. zwicki* | H&A | Ph | 116910 | 479 | 524 | 3 | 2339 | 4091 | -2.024 | 0.204 | 1.206 | 0.071 | 0.94 | [329] |
| Perlariopseidae  *Karanemoura*  *K. abrupta* | H&A | Pr | 350080 | 122 | 259 | 5 | 12962 | 19758 | -2.024 | 0.204 | 1.206 | 0.071 | 0.94 | [328] |
| *K. manca* | H&A | Pr | 126852 | 193 | 379 | 3 | 41056 |  | -2.024 | 0.204 | 1.206 | 0.071 | 0.94 | [328] |
| Pronemouridae  *Pronemoura*  *P. angustithorax* | H&A | Ph | 315744 | 414 | 454 | 2 |  | 21114 | -2.024 | 0.204 | 1.206 | 0.071 | 0.94 | [330] |
| *P. longialata* | H&A | Ph | 218160 | 228 | 262 | 2 | 11559 | 13460 | -2.024 | 0.204 | 1.206 | 0.071 | 0.94 | [330] |
| *P. minuta* | H&A | Ph | 166590 | 136 | 183 | 2 | 3294 | 7603 | -2.024 | 0.204 | 1.206 | 0.071 | 0.94 | [330] |
| *P. peculiaris* | H&A | Ph | 199564 | 206 | 261 | 2 | 7838 | 12783 | -2.024 | 0.204 | 1.206 | 0.071 | 0.94 | [330] |
| *P. shii* | H&A | Ph | 377804 | 380 | 446 | 2 | 13901 | 22860 | -2.024 | 0.204 | 1.206 | 0.071 | 0.94 | [330] |
| Pteronarcyidae  *Pteroliriope*  *P. sinitshenkovae* | H&A | Ph | 107310 | 30 | 65 | 5 | 1827 | 7897 | -2.024 | 0.204 | 1.206 | 0.071 | 0.94 | [331] |
| Taeniopterygidae  *Jurataenionema*  *J. inornatus* | H&A | Ph | 150350 | 203 | 247 | 2 | 15965 | 12447 | -2.024 | 0.204 | 1.206 | 0.071 | 0.94 | [332] |
| *J. stigmaeus* | H&A | Ph | 393680 | 273 | 320 | 2 | 19488 |  | -2.024 | 0.204 | 1.206 | 0.071 | 0.94 | [332] |
| *Mengitaenioptera*  *M. multiramis* | H&A | Ph | 55680 | 120 | 169 | 5 | 5800 | 7205 | -2.024 | 0.204 | 1.206 | 0.071 | 0.94 | [333] |
| *Noviramonemoura*  *N. trinervis* | H&A | Ph | 77088 | 44 | 139 | 5 | 11075 | 14321 | -2.024 | 0.204 | 1.206 | 0.071 | 0.94 | [333] |
| *Protaenionema*  *P. fuscalatus* | H&A | Ph | 133770 | 300 | 333 | 2 | 20717 | 159499 | -2.024 | 0.204 | 1.206 | 0.071 | 0.94 | [332] |
| **Raphidioptera**  Juroraphidiidae  *Juroraphidia*  *J. longicollum* | A&S | Pr | 410350 | 35 | 68 | 1 | 7174 | 10184 | -2.14 | 0.036 | 1.331 | 0.011 | 0.94 | [334] |
| Mesoraphidiidae  *Mesoraphidia*  *M. daohugouensis* | A&S | Pr | 243820 | 55 | 83 | 1 | 8474 |  | -2.14 | 0.036 | 1.331 | 0.011 | 0.94 | [335] |
| *Ororaphidia*  *O. bifurcata* | A&S | Pr | 193110 | 46 | 93 | 1 | 31708 |  | -2.14 | 0.036 | 1.331 | 0.011 | 0.94 | [336] |
| *O. megalocephala* | A&S | Pr |  |  |  |  |  |  | -2.14 | 0.036 | 1.331 | 0.011 | 0.94 | [337] |
| *Styporaphidia*  *S. magia* | A&S | Pr |  |  |  |  |  |  | -2.14 | 0.036 | 1.331 | 0.011 | 0.94 | [337] |
| **Siphonaptera**  Pseudopulicidae  *Hadropsylla*  *H. sinica* | S | Pa | 351995 | 216 | 238 | 2 | 11460 |  | -2.14 | 0.036 | 1.331 | 0.011 | 0.94 | [338] |
| *Pseudopulex*  *P. jurassicus* | S | Pa | 182600 | 171 | 185 | 1 | 14464 |  | -2.14 | 0.036 | 1.331 | 0.011 | 0.94 | [339] |
| *P. wangi* | S | Pa | 125730 | 224 | 259 | 2 | 18506 |  | -2.14 | 0.036 | 1.331 | 0.011 | 0.94 | [338] |
| **Thripida (superord)**  Lophioneurida (subord.)  *Lophiosina*  *L. lini* | S | Ph | 351505 | 301 | 359 | 1 | 2656 | 2964 | -2.14 | 0.036 | 1.331 | 0.011 | 0.94 | [340] |
| Undacypha  U. bournieri | S | Ph | 340615 | 128 | 210 | 0.5 |  | 31471 | -2.14 | 0.036 | 1.331 | 0.011 | 0.94 | [340] |
| U. kreiteri | S | Ph | 222650 | 298 | 354 | 1 |  | 11459 | -2.14 | 0.036 | 1.331 | 0.011 | 0.94 | [340] |
| **Trichoptera**  Hydrobiosidae  *Juraphilopotamus*  *J. lubricus* | H&S | Ph&Pr |  |  |  |  |  |  | -2.369 | 0.132 | 1.501 | 0.055 | 0.95 | [341] |
| *Pulchercylindratus*  *P. punctatus* | H&S | Ph&Pr | 135072 | 190 | 245 | 2 | 14837 | 18677 | -2.369 | 0.132 | 1.501 | 0.055 | 0.95 | [342] |
| Necrotauliidae  Acisarcuatus  A. locellatus | H&S | Ph&Pr | 293643 | 60 | 126 | 1 | 29370 | 35882 | -2.369 | 0.132 | 1.501 | 0.055 | 0.95 | [343] |
| A. variradius | H&S | Ph&Pr | 72252 | 219 | 286 | 4 | 6151 | 7158 | -2.369 | 0.132 | 1.501 | 0.055 | 0.95 | [344] |
| Philopotamidae  Juraphilopotamus  J. lubricus | H&S | Ph&Pr | 82656 | 410 | 458 | 1 | 27581 |  | -2.369 | 0.132 | 1.501 | 0.055 | 0.95 | [341] |
| Liadotaulius  L. daohugouensis | H&S | Ph&Pr | 81774 | 90 | 166 | 2 |  | 31187 | -2.369 | 0.132 | 1.501 | 0.055 | 0.95 | [345] |
| L. limus | H&S | Ph&Pr | 179550 | 121 | 154 | 1 |  | 10622 | -2.369 | 0.132 | 1.501 | 0.055 | 0.95 | [343] |
| Rhyacophilidae  Declinimodus  D. setulosus | H&S | Ph&Pr | 264120 | 343 | 393 | 2 | 30980 | 31159 | -2.369 | 0.132 | 1.501 | 0.055 | 0.95 | [346] |

^1^Habitat and feeding class designations.

| Habitat |  |  | Feeding  Class |  |  | Eosesatheris/mm^2^ |
| --- | --- | --- | --- | --- | --- | --- |
| A: | Alpine |  | Om: | Omnivory |  | 17.65 |
| E: | Edaphic |  | Pa: | Parasitism |  |  |
| H: | Hydrophytic | | Ph: | Phytophagy | |  |
| S: | Silvan |  | Pr: | Predacity |  |  |
|  |  |  | Sa: | Saprophagy | |  |

References:

1. Xw Z, Dong R, Pang H. A Water-Skiing Chresmodid From The Middle Jurassic In Daohugou, Inner Mongolia, China (Polyneoptera: Orthopterida). *Zootaxa*. 2008; **1762**: 53–62. doi: 10.5281/zenodo.181955

2. Zhang X, Ren D, Pang H *et al.* Late Mesozoic Chresmodids with Forewing from Inner Mongolia, China (Polyneoptera: Archaeorthoptera). *Acta Geologica Sinica - English Edition*. 2010; **84**(1): 38–46. doi: 10.1111/j.1755-6724.2010.00168.x

3. Huang D, Petrulevicius J, Nel A. New morphological data from the Jurassic of Inner Mongolia confirms the damselfly aspect of Protomyrmeleontidae (Insecta: Odonatoptera). *European Journal of Entomology*. 2010; **107**: 615–620.

4. Wei D, Shih C, Ren D. Arcofuzia cana gen. et sp. n. (Insecta, Blattaria, Fuziidae) from the Middle Jurassic sediments of Inner Mongolia, China. *Zootaxa*. 2012; **3597**(1): 25–32. doi: 10.11646/zootaxa.3597.1.3

5. Wei D, Liang J, Ren D. A new fossil genus of Fuziidae (Insecta, Blattida) from the Middle Jurassic of Jiulongshan Formation, China. *Geodiversitas*. 2013; **35**(2): 335–343. doi: 10.5252/g2013n2a3

6. Vršanský P, Liang J-H, Ren D. Advanced morphology and behaviour of extinct earwig-like cockroaches (Blattida: Fuziidae fam. nov.). *Geologica Carpathica*. 2009; **60**(6): 449–462. doi: 10.2478/v10096-009-0033-0

7. Guo Y, Ren D. A New Cockroach Genus of the Family Fuziidae from Northeastern China (Insecta: Blattida). *Acta Geologica Sinica - English Edition*. 2011; **85**(2): 501–506. doi: 10.1111/j.1755-6724.2011.00418.x

8. Dong R, Dan W, Junhui L. A new species of Fuziidae (Insecta, Blattida) from the Inner Mongolia, China. *ZooKeys*. 2012; **217**: 53–61. doi: 10.3897/zookeys.217.3508

9. Vršanský P, Liang J-H, Ren D. Malformed cockroach (Blattida: Liberiblattinidae) in the Middle Jurassic sediments from China. *Oriental Insects*. 2012; **46**(1): 12–18. doi: 10.1080/00305316.2012.675482

10. Liang J, Vrsansky P, Ren D. Variability and symmetry of a Jurassic nocturnal predatory cockroach (Blattida: Raphidiomimidae). *Revista Mexicana de Ciencias Geologicas*. 2012; **29**: 411–421.

11. Liang J, Shih C, Ren D. New Jurassic predatory cockroaches (Blattaria: Raphidiomimidae) from Daohugou, China and Karatau, Kazakhstan. *Alcheringa: An Australasian Journal of Palaeontology*. 2017; **42**(1): 101–109. doi: 10.1080/03115518.2017.1374460

12. Liang J-H, VrŠAnsku P, Ren D *et al.* A new Jurassic carnivorous cockroach (Insecta, Blattaria, Raphidiomimidae) from the Inner Mongolia in China *Zootaxa*. 2009; **1974**(1): 17–30. doi: 10.11646/zootaxa.1974.1.2

13. Liang J-H, Huang W-L, Ren D. Graciliblatta bella gen. et sp.n. - a rare carnivorous cockroach (Insecta, Blattida, Raphidiomimidae) from the Middle Jurassic sediments of Daohugou in Inner Mongolia, China. *Zootaxa*. 2012; **3449**(1): 62–68. doi: 10.11646/zootaxa.3449.1.4

14. Tan J, Ren D. Two Exceptionally Well-Preserved Catiniids (Coleoptera: Archostemata: Catiniidae) from the Late Mesozoic of Northeastern China. *Annals of the Entomological Society of America*. 2007; **100**(5): 666–672. doi: 10.1603/0013-8746(2007)100[666:Tewcca]2.0.Co;2

15. Kolibac J, Huang D. New cleroid beetles from the Middle-Late Jurassic of China. *Acta Palaeontologica Polonica*. 2019; **64**: 143–155. doi: 10.4202/app.00550.2018

16. Hong Y. *Middle Jurassic Fossil Insects in North China*. Beijing: Geological Publishing House, 1983.

17. Yan EV, Wang B, Jarzembowski EA *et al.* The earliest byrrhoids (Coleoptera, Elateriformia) from the Jurassic of China and their evolutionary implications. *Proceedings of the Geologists' Association*. 2015; **126**(2): 211–219. doi: 10.1016/j.pgeola.2015.01.009

18. Yu Y, Leschen RAB, Slipinski A *et al.* The First Fossil Bark-Gnawing Beetle from the Middle Jurassic of Inner Mongolia, China (Coleoptera: Trogossitidae). *Annales Zoologici*. 2012; **62**(2): 245–252. doi: 10.3161/000345412x652765

19. Wang B, Zhang H. The oldest Tenebrionoidea (Coleoptera) from the Middle Jurassic of China. *Journal of Paleontology*. 2011; **85**: 266–270. doi: 10.2307/23020043

20. Bai M, Ahrens D, Yang XK *et al.* New fossil evidence of the early diversification of scarabs:Alloioscarabaeus cheni(Coleoptera: Scarabaeoidea) from the Middle Jurassic of Inner Mongolia, China. *Insect Science*. 2012; **19**(2): 159–171. doi: 10.1111/j.1744-7917.2011.01460.x

21. Cai CY, Lawrence JF, ŚLipiŃSki A *et al.* Jurassic artematopodid beetles and their implications for the early evolution of Artematopodidae (Coleoptera). *Systematic Entomology*. 2015; **40**(4): 779–788. doi: 10.1111/syen.12131

22. Zhang J-F. The first find of chrysomelids (Insecta: Coleopetra: Chrysomeloidea) from Callovian–Oxfordian Daohugou biota of China. *Geobios*. 2005; **38**(6): 865–871. doi: 10.1016/j.geobios.2004.05.003

23. Tan J, Ren D. *Mesozoic Archostematan Fauna from China*2009.

24. Liu Z, Ślipiński A, Lawrence JF *et al.* Palaeoboganium gen. nov. from the Middle Jurassic of China (Coleoptera: Cucujoidea: Boganiidae): the first cycad pollinators. *Journal of Systematic Palaeontology*. 2017; **16**(4): 351–360. doi: 10.1080/14772019.2017.1304459

25. Pan X, Chang H, Ren D *et al.* The first fossil buprestids from the Middle Jurassic Jiulongshan Formation of China (Coleoptera: Buprestidae). *Zootaxa*. 2011; **2745**(1): 53–62. doi: 10.11646/zootaxa.2745.1.4

26. Yu Y, Ślipiński A, Lawrence JF *et al.* Reconciling past and present: Mesozoic fossil record and a new phylogeny of the family Cerophytidae (Coleoptera: Elateroidea). *Cretaceous Research*. 2019; **99**: 51–70. doi: 10.1016/j.cretres.2019.02.024

27. KolibÁČ J, Huang D. The oldest known clerid fossils from the Middle Jurassic of China, with a review of Cleridae systematics (Coleoptera). *Systematic Entomology*. 2016; **41**(4): 808–823. doi: 10.1111/syen.12192

28. Wang B, Ponomarenko AG, Zhang HC. A new coptoclavid larva (Coleoptera: Adephaga: Dytiscoidea) from the Middle Jurassic of China, and its phylogenetic implication. *Paleontological Journal*. 2009; **43**(6): 652–659. doi: 10.1134/s0031030109060082

29. Tan J, Ren D, Shih C. New cupedids from the Middle Jurassic of Inner Mongolia, China (Coleoptera: Archostemata). *Annales Zoologici*. 2006; **56**: 1–6.

30. Jingjing T, Diying H, Dong R. First Record of Fossil Mesocupes from China (Coleoptera: Archostemata: Cupedidae). *Acta Geologica Sinica - English Edition*. 2011; **81**(5): 688–696. doi: 10.1111/j.1755-6724.2007.tb00993.x

31. Deng C, Ślipiński A, Ren D *et al.* The Oldest Dermestid Beetle from the Middle Jurassic of China (Coleoptera: Dermestidae). *Annales Zoologici*. 2017; **67**(1): 109–112. doi: 10.3161/00034541anz2017.67.1.012

32. Cai C, Lawrence JF, ŚLipiŃSki A *et al.* First fossil tooth-necked fungus beetle (Coleoptera: Derodontidae): Juropeltastica sinica gen. n. sp. n. from the Middle Jurassic of China. *European Journal of Entomology*. 2014; **111**(2): 299–302. doi: 10.14411/eje.2014.034

33. Fabing D, Diying H. A New Elaterid from the Middle Jurassic Daohugou Biota (Coleoptera: Elateridae: Protagrypninae). *Acta Geologica Sinica - English Edition*. 2011; **85**(6): 1224–1230. doi: 10.1111/j.1755-6724.2011.00583.x

34. Chang H, Kirejtshuk AG, Ren D *et al.* First Fossil Click Beetles from the Middle Jurassic of Inner Mongolia, China (Coleoptera: Elateridae). *Annales Zoologici*. 2009; **59**(1): 7–14. doi: 10.3161/000345409x432547

35. Chang H, Zhao Y, Ren D. New fossil elaterids (Insect: Coleoptera: Polyphaga: Elateridae) from the Middle Jurassic of Inner Mongolia, China. *Progress in Natural Science*. 2009; **19**(10): 1433–1437. doi: 10.1016/j.pnsc.2009.04.006

36. Yan EV, Wang B, Ponomarenko AG *et al.* The most mysterious beetles: Jurassic Jurodidae (Insecta: Coleoptera) from China. *Gondwana Research*. 2014; **25**(1): 214–225. doi: 10.1016/j.gr.2013.04.002

37. Kirejtshuk AG, Chang H, Ren D *et al.* Family Lasiosynidae n. fam., new palaeoendemic Mesozoic family from the infraorder Elateriformia (Coleoptera: Polyphaga). *Annales de la Société entomologique de France (NS)*. 2013; **46**(1-2): 67–87. doi: 10.1080/00379271.2010.10697640

38. Tan J, Dong R, Shih C. New beetles (Insecta: Coleoptera: Archostemata) from the Late Mesozoic of North China. *Annales Zoologici*. 2007; **57**(2): 231–247.

39. Yan EV, Wang B, Zhang H. A new lasiosynid beetle from the Middle Jurassic of China with remarks on the systematic position of Lasiosynidae. *Comptes Rendus Palevol*. 2014; **13**(1): 1–8. doi: 10.1016/j.crpv.2013.06.001

40. Yan EV, Wang B. A new genus of Elateriform beetles (Coleoptera, Polyphaga) from the Jurassic of Daohugou, China. *Paleontological Journal*. 2010; **44**(3): 297–302. doi: 10.1134/s0031030110030093

41. Nikolajev GV, Wang B, Liu Y *et al.* Stag beetles from the Mesozoic of Inner Mongolia, China (Scarabaeoidea: Lucanidae). *Acta Palaeontologica Sinica*. 2011; **50**: 41–47.

42. Cai C-Y, Ślipiński A, Huang D-Y. The oldest root-eating beetle from the Middle Jurassic of China (Coleoptera, Monotomidae). *Alcheringa: An Australasian Journal of Palaeontology*. 2015; **39**(4): 488–493. doi: 10.1080/03115518.2015.1037173

43. Nikolajev GV, Ren D. The oldest fossil Ochodaeidae (Coleoptera: Scarabaeoidea) from the Middle Jurassic of China. *Zootaxa*. 2010; **2553**: 65–68. doi: 10.5281/zenodo.196844

44. Ponomarenko A. First record of Notocupes (Coleoptera: Cupedidae) in locality Daohugou, Middle Jurassic of Inner Mongolia, China. *Annales Zoologici*. 2010; **60**: 169–171.

45. Tan J, Wang Y, Ren D *et al.* New fossil species of ommatids (Coleoptera: Archostemata) from the Middle Mesozoic of China illuminating the phylogeny of Ommatidae. *BMC Evolutionary Biology*. 2012; **12**(1): 1–19. doi: 10.1186/1471-2148-12-113

46. Lu T, Zhao Y, Shih C *et al.* Three new species of Parandrexis (Coleoptera: Parandrexidae) from the Middle Jurassic Jiulongshan Formation of Inner Mongolia, China. *Entomotaxonomia*. 2015; **37**(2): 111–122. doi: 10.11680/entomotax.2015017

47. Liu Z, Ślipiński A, Leschen RAB *et al.* The Oldest Prionoceridae (Coleoptera: Cleroidea) from the Middle Jurassic of China. *Annales Zoologici*. 2015; **65**(1): 41–52. doi: 10.3161/00034541anz2015.65.1.004

48. Hsiao Y, Yu Y, Deng C *et al.* The first fossil wedge-shaped beetle (Coleoptera, Ripiphoridae) from the middle Jurassic of China. *European Journal of Taxonomy*. 2017(277). doi: 10.5852/ejt.2017.277

49. Tan J, Ren D, Shih C *et al.* New schizophorid fossils from China and possible evolutionary scenarios for Jurassic archostematan beetles. *Journal of Systematic Palaeontology*. 2013; **11**(1): 47–62. doi: 10.1080/14772019.2011.637515

50. Jarzembowski EA, Yan EV, Wang B *et al.* A new flying water beetle (Coleoptera: Schizophoridae) from the Jurassic Daohugou lagerstätte. *Palaeoworld*. 2012; **21**(3-4): 160–166. doi: 10.1016/j.palwor.2012.09.002

51. Cai C, Huang D, Thayer MK *et al.* Glypholomatine rove beetles (Coleoptera: Staphylinidae): a southern hemisphere Recent group recorded from the Middle Jurassic of China. *Journal of the Kansas Entomological Society*. 2012; **85**(3): 239–244. doi: 10.2307/23355954

52. Cai C, Huang D, Newton AF *et al.* Mesapatetica aenigmatica, a New Genus and Species of Rove Beetles (Coleoptera, Staphylinidae) from the Middle Jurassic of China. *Journal of the Kansas Entomological Society*. 2014; **87**(2): 219–224. doi: 10.2317/jkes130427.1

53. Cai C, Beattie R, Huang D. Jurassic olisthaerine rove beetles (Coleoptera: Staphylinidae): 165 million years of morphological and probably behavioral stasis. *Gondwana Research*. 2015; **28**(1): 425–431. doi: 10.1016/j.gr.2014.03.007

54. Cai C-Y, Huang D-Y. Sinanthobium daohugouense, a tiny new omaliine rove beetle (Coleoptera: Staphylinidae) from the Middle Jurassic of China. *The Canadian Entomologist*. 2013; **145**(5): 496–500. doi: 10.4039/tce.2013.33

55. Wang BO, Zhang H, Ponomarenko AG. Mesozoic Trachypachidae (Insecta: Coleoptera) from China. *Palaeontology*. 2012; **55**(2): 341–353. doi: 10.1111/j.1475-4983.2012.01128.x

56. Yu Y, Ślipiński A, Leschen RAB *et al.* Enigmatic Mesozoic Bark-Gnawing Beetles (Coleoptera: Trogossitidae) from the Jiulongshan Formation in China. *Annales Zoologici*. 2014; **64**(4): 667–676. doi: 10.3161/000345414x685947

57. Zhao JX, Ren D, Shih C. Enigmatic earwig‐like fossils from Inner Mongolia, China. *Insect Science*. 2010; **17**(5): 459–464. doi: 10.1111/j.1744-7917.2010.01315.x

58. Zhao J, Zhao Y, Shih C *et al.* Transitional fossil earwigs - a missing link in Dermaptera evolution. *BMC Evolutionary Biology*. 2010; **10**(1): 1–10. doi: 10.1186/1471-2148-10-344

59. Zhang J. The most primitive earwigs (Archidermaptera, Dermaptera, Insecta) from the Upper Jurassic of the Nei Monggol Autonomous Region, northeastern China Acta Micropalaeontologica Sinica. *Acta Micropalaeontologica Sinica*. 2002; **19**(4): 348–362.

60. Jingxia Z, Chungkun S, Dong REN *et al.* New Primitive Fossil Earwig from Daohugou, Inner Mongolia, China (Insecta: Dermaptera: Archidermaptera). *Acta Geologica Sinica - English Edition*. 2011; **85**(1): 75–80. doi: 10.1111/j.1755-6724.2011.00380.x

61. Yin Y, Shih C, Engel MS *et al.* New Earwigs from the Middle Jurassic Jiulongshan Formation of Northeastern China (Dermaptera). *Insects*. 2023; **14**(7): 180–188. doi: 10.3390/insects14070614

62. Zhang J. Some anisopodoids (Insecta: Diptera: Anisopodoidea) from late Mesozoic deposits of northeast China. *Cretaceous Research*. 2007; **28**(2): 281–288. doi: 10.1016/j.cretres.2006.05.008

63. Wang F, Shih C, Ren D *et al.* Quantitative assessments and taxonomic revision of the genus Archirhagio with a new species from Daohugou, China (Diptera: Archisargidae). *Systematic Entomology*. 2017; **42**(1): 230–239. doi: 10.1111/syen.12204

64. Zhang K, Li J, Yang D *et al.* A new species of Archirhagio Rohdendorf, 1938 from the Middle Jurassic of Inner Mongolia of China (Diptera: Archisargidae). *Zootaxa*. 2009; **1984**(1984): 65–61. doi: 10.5281/zenodo.185382

65. Zhang K, Yang D, Ren D *et al.* The earliest species of the extinct genus Archisargus from China (Diptera: Brachycera: Archisargidae). *Annales Zoologici*. 2006; **57**(4): 827–832. doi: 10.3161/000345407783742006

66. Zhang K, Yang D, Ren D *et al.* The oldest Calosargus Mostovski, 1997 from the Middle Jurassic of China (Diptera: Brachycera: Archisargidae). *Zootaxa*. 2007; **1645**: 1–17. doi: 10.5281/zenodo.179727

67. Zhang JF. Records of bizarre Jurassic brachycerans in the Daohugou biota, China (Diptera, Brachycera, Archisargidae and Rhagionemestriidae). *Palaeontology*. 2010; **53**(2): 307–317. doi: 10.1111/j.1475-4983.2010.00934.x

68. Zhang K, Yang D, Ren D. Middle Jurassic fossils of the genus Sharasargus from Inner Mongolia, China (Diptera: Archisargidae). *Entomological Science*. 2008; **11**(2): 269–272. doi: 10.1111/j.1479-8298.2008.00271.x

69. Zhang J. Distinct but rare archisargid flies from Jurassic of China (Diptera, Brachycera, Archisargidae) with discussion of the systematic position of Origoasilus pingquanensis Zhang et al., 2011. *Journal of Paleontology*. 2016; **86**(5): 878–885. doi: 10.1666/12-028.1

70. Zhang J, Zhang H. Two new species of archisargids (Insecta: Diptera: Archisargidae) from the Upper Jurassic Daohugou Formation (Inner Mongolia, northeastern China). *Paleontological Journal*. 2003; **37**: 409–412.

71. Zhang K, Yang D, Ren D *et al.* New archisargids from China (Insecta: Diptera). *Entomological Science*. 2010; **13**(1): 75–80. doi: 10.1111/j.1479-8298.2009.00350.x

72. Zhang J. Archisargoid flies (Diptera, Brachycera, Archisargidae and Kovalevisargidae) from the Jurassic Daohugou biota of China, and the related biostratigraphical correlation and geological age. *Journal of Systematic Palaeontology*. 2014; **13**(10): 857–881. doi: 10.1080/14772019.2014.960902

73. Junfeng Z. Orientisargidae fam. n., a new Jurassic family of Archisargoidea (Diptera, Brachycera), with review of Archisargidae from China. *ZooKeys*. 2012; **238**: 57–76. doi: 10.3897/zookeys.238.3624

74. Zhang K, Yang D, Ren D *et al.* An evolutional special case in the lower Orthorrhapha: some attractive fossil flies from the Middle Jurassic of China (Insecta: Diptera: Brachycera). *Zoological Journal of the Linnean Society*. 2010; **158**(3): 563–572. doi: 10.1111/j.1096-3642.2009.00552.x

75. Chen J, Wang B, Engel MS *et al.* Extreme adaptations for aquatic ectoparasitism in a Jurassic fly larva. *eLife*. 2014; **3**. doi: 10.7554/eLife.02844

76. Zhang J. Two New Genera and One New Species of Jurassic Axymyiidae (Diptera: Nematocera), with Revision and Redescription of the Extinct Taxa. *Annals of the Entomological Society of America*. 2010; **103**(4): 455–464. doi: 10.1603/an09073

77. Zhang J-F. First description of axymyiid fossils (Insecta: Diptera: Axymyiidae). *Geobios*. 2004; **37**(5): 687–694. doi: 10.1016/j.geobios.2003.04.007

78. Guifeng S, Yan Z, Chungkun S *et al.* A New Axymyiid Genus with Two New Species from the Middle Jurassic of China (Diptera: Nematocera: Axymyiidae). *Acta Geologica Sinica - English Edition*. 2013; **87**(5): 1228–1234. doi: 10.1111/1755-6724.12123

79. Lukashevich ED, Huang D, Lin QB. Rare families of lower Diptera (Hennigmatidae, Blephariceridae, Perissommatidae) from the Jurassic of China. *Studia Dipterologica*. 2006; **13**: 127–143.

80. Zhang J, Lukashevich ED. The oldest known net-winged midges (Insecta: Diptera: Blephariceridae) from the late Mesozoic of northeast China. *Cretaceous Research*. 2007; **28**: 302–309. doi: 10.1016/j.cretres.2006.05.012

81. Zhang J. Three distinct but rare kovalevisargid flies from the Jurassic Daohugou biota, China (Insecta, Diptera, Brachycera, Kovalevisargidae). *Palaeontology*. 2011; **54**(1): 163–170. doi: 10.1111/j.1475-4983.2010.01010.x

82. Zhang J. Nematoceran dipterans from the Jurassic of China (Insecta, Diptera: Limoniidae, Tanyderidae). *Paleontological Journal*. 2004; **5**(5): 53–57.

83. Hao J-y, Ren D. Two New Fossil Species of Limoniidae (Diptera: Nematocera) from the Middle Jurassic of Northeastern, China. *Entomological News*. 2009; **120**(2): 171–178. doi: 10.3157/021.120.0206

84. Zhang J. Jurassic Limoniid dipterans from China (Diptera: Limoniidae). *Oriental Insects*. 2012; **40**(1): 115–126. doi: 10.1080/00305316.2006.10417463

85. Jiaqi G, Chungkun S, Yunyun Z *et al.* New Species of Cretolimonia and Mesotipula (Diptera: Limoniidae) from the Middle Jurassic of Northeastern China. *Acta Geologica Sinica - English Edition*. 2015; **89**(6): 1789–1796. doi: 10.1111/1755-6724.12597

86. Li T, Ren D. A new fossil genus of Mesosciophilidae (Diptera, Nematocera) with two new species from the Middle Jurassic of Inner Mongolia, China. *Progress in Natural Science*. 2009; **19**(12): 1837–1841. doi: 10.1016/j.pnsc.2009.06.011

87. Zhang J. Three New Species of Mesosciophilid Gnats from the Middle-Late Jurassic of China (Insecta: Diptera: Nematocera: Mesosciophilidae). *Pakistan Journal of Biological Sciences*. 2008; **11**(22): 2567–2572. doi: 10.3923/pjbs.2008.2567.2572

88. Hao J, Ren D. Middle Jurassic Protopleciidae from Daohugou, Inner Mongolia, China (Insecta, Diptera). *Acta Zootaxonomica Sinica*. 2009; **34**: 554–559.

89. Zhang J. New mesosciophilid gnats (Insecta: Diptera: Mesosciophilidae) in the Daohugou biota of Inner Mongolia, China. *Cretaceous Research*. 2007; **28**(2): 297–301. doi: 10.1016/j.cretres.2006.05.007

90. Wang Q, Zhao Y, Ren D. Two new species of Mesosciophilidae (Insecta: Diptera: Nematocera) from the Yanliao biota of Inner Mongolia, China. *Alcheringa: An Australasian Journal of Palaeontology*. 2012; **36**(4): 509–514. doi: 10.1080/03115518.2012.685434

91. Dong R, Gao J, Guifeng S *et al.* Two new species of Paramesosciophilodes (Diptera, Nematocera, Mesosciophilidae) from the Middle Jurassic of China. *ZooKeys*. 2015; **511**: 117–129. doi: 10.3897/zookeys.511.8425

92. Shi G, Shih C, Ren D. A new genus with two new species of mesosciophilids from the Middle Jurassic of China (Diptera: Nematocera: Mesosciophilidae). *Journal of Natural History*. 2014; **49**(19-20): 1147–1158. doi: 10.1080/00222933.2014.951085

93. Zhang K, Yang D, Ren D *et al.* New Middle Jurassic Tangle-Veined Flies from Inner Mongolia, China. *Acta Palaeontologica Polonica*. 2008; **53**(1): 161–164. doi: 10.4202/app.2008.0112

94. Gao J, Shih C, KopeĆ K *et al.* New species and revisions of Pediciidae (Diptera) from the Middle Jurassic of northeastern China and Russia. *Zootaxa*. 2015; **3964**(1): 240–249. doi: 10.11646/zootaxa.3964.1.4

95. Zhang K, Yang D, Ren D. The first Middle Jurassic Protobrachyceron Handlirsch fly (Diptera: Brachycera: Protobrachyceridae) from Inner Mongolia (China). *Zootaxa*. 2008; **1879**: 61–64. doi: 10.5281/zenodo.184177

96. Dong R, Lin X, Shih C. Revision of the genus Epimesoplecia Zhang, 2007 (Diptera, Nematocera, Protopleciidae) with five new species. *ZooKeys*. 2015; **492**: 123–143. doi: 10.3897/zookeys.492.6852

97. Zhang J. New Mesozoic Protopleciidae (Insecta: Diptera: Nematocera) from China. *Cretaceous Research*. 2007; **28**(2): 289–296. doi: 10.1016/j.cretres.2006.05.009

98. Lin X, Shih C, Ren D. Two new species of Mesoplecia (Insecta: Diptera: Protopleciidae) from the late Middle Jurassic of China. *Zootaxa*. 2014; **3838**(5): 545–556. doi: 10.11646/zootaxa.3838.5.3

99. Hao J, Dong K, Ren D. Middle Jurassic Eoptychopteridae from Daohugou, Inner Mongolia, China (Insecta, Diptera, Eoptychopteridae). *Acta Zootaxonomica Sinica*. 2009; **34**(1): 106–110.

100. Ren D, Krzeminski W. Eoptychopteridae (Diptera) from the Middle Jurassic of China. *Annales Zoologici*. 2002; **52**(2): 207–210.

101. Hao J, Ren D, Shih C. New Fossils of Eoptychopteridae (Diptera) from the Middle Jurassic of Northeastern China. *Acta Geologica Sinica - English Edition*. 2009; **83**(2): 222–228. doi: 10.1111/j.1755-6724.2009.00022.x

102. Liu L, Shih C, Ren D. Two new species of Ptychopteridae and Trichoceridae from the Middle Jurassic of northeastern China (Insecta: Diptera: Nematocera). *Zootaxa*. 2012; **3501**(1): 55–62. doi: 10.11646/zootaxa.3501.1.2

103. Lin Q, Lukashevich ED. Proptychopterina (Diptera: Eoptychopteridae) from the Jurassic of Northeastern China. *Paleontological Journal*. 2006; **40**(3): 290–294. doi: 10.1134/s0031030106030099

104. Zhang J-F. New species of Palaeobolbomyia Kovalev and Ussatchovia Kovalev (Diptera, Brachycera, Rhagionidae) from the Callovian-Oxfordian (Jurassic) Daohugou biota of China: Biostratigraphic and paleoecologic implications. *Geobios*. 2010; **43**(6): 663–669. doi: 10.1016/j.geobios.2010.06.004

105. Zhang K, Yang D, Ren D. A new genus and species of Middle Jurassic rhagionids from China (Diptera, Rhagionidae). *Biologia*. 2008; **63**(1): 113–116. doi: 10.2478/s11756-008-0012-4

106. Zhang J. Snipe flies (Diptera: Rhagionidae) from the Daohugou Formation (Jurassic), Inner Mongolia, and the systematic position of related records in China. *Palaeontology*. 2013; **56**(1): 217–228. doi: 10.1111/j.1475-4983.2012.01192.x

107. Zhang J, Li H. New taxa of snipe flies (Diptera: Brachycera: Rhagionidae) in the Daohugou Biota, China. *Paleontological Journal*. 2012; **46**(2): 157–163. doi: 10.1134/s0031030112020128

108. Zhang J. Two new species of Palaeoarthroteles Kovalev and Mostovski (Diptera, Rhagionidae) from the Callovian-Oxfordian (Jurassic) Daohugou biota of China. *Geobios*. 2011; **44**(6): 635–639. doi: 10.1016/j.geobios.2011.01.006

109. Zhang K, Yang D, Ren D. The first snipe fly (Diptera: Rhagionidae) from the Middle Jurassic of Inner Mongolia, China. *Zootaxa*. 2006; **1134**: 51–57. doi: 10.5281/zenodo.171918

110. Huang D, Nel A, Cai C *et al.* Amphibious flies and paedomorphism in the Jurassic period. *Nature*. 2013; **495**(7439): 94–97. doi: 10.1038/nature11898

111. Lukashevich ED, Krzeminski W. New Jurassic Tanyderidae (Diptera) from Asia with first find of larvae. *Zoosymposia*. 2009; **3**(1): 155–172. doi: 10.11646/zoosymposia.3.1.13

112. Dong F, Shih C, Skibińska K *et al.* New species of Tanyderidae (Diptera) from the Jiulongshan Formation of China. *Alcheringa: An Australasian Journal of Palaeontology*. 2015; **39**(4): 494–507. doi: 10.1080/03115518.2015.1041308

113. Dong F, Shih C, Ren D. Two new species of Trichoceridae from the Middle Jurassic Jiulongshan Formation of Inner Mongolia, China. *ZooKeys*. 2014; **411**: 145–160. doi: 10.3897/zookeys.411.6858

114. Krzemiska E, Krzeminski W, Dahl C. *Monograph of Fossil Trichoceridae (Diptera): Over 180 Million Years of Evolution*: Polish Academy of Sciences, 2009.

115. Zhang J. New winter crane flies (Insecta: Diptera: Trichoceridae) from the Jurassic Daohugou Formation (Inner Mongolia, China) and their associated biota. *Canadian Journal of Earth Sciences*. 2006; **43**(1): 9–22. doi: 10.1139/e05-092

116. Huang D-Y, Nel A. Oldest webspinners from the Middle Jurassic of Inner Mongolia, China (Insecta: Embiodea). *Zoological Journal of the Linnean Society*. 2009; **156**(4): 889–895. doi: 10.1111/j.1096-3642.2008.00499.x

117. Zhang J-F, Kluge NJ. Jurassic larvae of mayflies (Ephemeroptera) from the Daohugou formation in Inner Mongolia, China. *Oriental Insects*. 2007; **41**(1): 351–366. doi: 10.1080/00305316.2007.10417519

118. Lin Q, Huang D. New Middle Jurassic Mayflies (Insecta: Ephemeroptera: Siphlonuridae) from Inner Mongolia, China. *Annales Zoologici*. 2008; **58**(3): 521–527. doi: 10.3161/000345408x364346

119. Huang JD, Ren D, Sinitshenkova ND *et al.* New fossil mayflies (Insecta: Ephemeroptera) from the Middle Jurassic of Daohugou, Inner Mongolia, China. *Insect Science*. 2008; **15**(2): 193–198. doi: 10.1111/j.1744-7917.2008.00200.x

120. Huang J, Liu Y, Sinitshenkova ND *et al.* A new fossil genus of Siphlonuridae (Insecta: Ephemeroptera) from the Daohugou, Inner Mongolia, China. *Annales Zoologici*. 2007; **57**(2): 221–225.

121. Zhang J. New mayfly nymphs from the Jurassic of Northern and Northeastern China (Insecta: Ephemeroptera). *Paleontological Journal*. 2006; **40**(5): 553–559. doi: 10.1134/s0031030106050091

122. Cui Y, Béthoux O, Klass K-D *et al.* The Jurassic Bajanzhargalanidae (Insecta: Grylloblattida?): New genera and species, and data on postabdominal morphology. *Arthropod Structure & Development*. 2015; **44**(6): 688–716. doi: 10.1016/j.asd.2015.04.008

123. Cui Y. New data on the Blattogryllidae-Plesioblattogryllidae-Grylloblattidae complex (Insecta: Grylloblattida: Blattogryllopterida tax.n.). *Arthropod Systematics & Phylogeny*. 2012; **70**(3): 167–180. doi: 10.3897/asp.70.e31760

124. Huang D-Y, Nel A, PetruleviČIus JF. New evolutionary evidence of Grylloblattida from the Middle Jurassic of Inner Mongolia, north-east China (Insecta, Polyneoptera). *Zoological Journal of the Linnean Society*. 2008; **152**(1): 17–24. doi: 10.1111/j.1096-3642.2007.00351.x

125. Ren D, Aristov DS. A new species of Plesioblattogryllus Huang, Nel et Petrulevičius (Grylloblattida: Plesioblattogryllidae) from the Middle Jurassic of China. *Paleontological Journal*. 2011; **45**(3): 273–274. doi: 10.1134/s0031030111030178

126. Cui Y, Storozhenko SY, Ren D. New and little-known species of Geinitziidae (Insecta: Grylloblattida) from the Middle Jurassic of China, with notes on taxonomy, habitus and habitat of these insects. *Alcheringa: An Australasian Journal of Palaeontology*. 2012; **36**(2): 251–261. doi: 10.1080/03115518.2012.628806

127. Huang D-Y, Nel A. A new Middle Jurassic "grylloblattodean" family from China (Insecta: Juraperlidae fam. n.). *European Journal of Entomology*. 2007; **104**(4): 837–840. doi: 10.14411/eje.2007.104

128. Yingying C, BÉThoux O, Chungkun S *et al.* A New Species of the Family Juraperlidae (Insecta: Grylloblattida) from the Middle Jurassic of China. *Acta Geologica Sinica - English Edition*. 2010; **84**(4): 710–713. doi: 10.1111/j.1755-6724.2010.00274.x

129. Zhang J. Revision and description of water boatmen from the Middle-Upper Jurassic of Northern and Northeastern China (Insecta: Hemiptera: Heteroptera: Corixidae). *Paleontological Journal*. 2010; **44**(5): 515–525. doi: 10.1134/s0031030110050060

130. Li S, Szwedo J, Ren D *et al.* Fenghuangor imperator gen. et sp. nov. of Fulgoridiidae from the Middle Jurassic of Daohugou Biota (Hemiptera: Fulgoromorpha). *Zootaxa*. 2011; **3094**(1): 52–62. doi: 10.11646/zootaxa.3094.1.4

131. Yao Y, Cai W, Ren D. The oldest known fossil plant bug (Hemiptera: Miridae), from Middle Jurassic of Inner Mongolia, China. *Zootaxa*. 2007; **1442**: 37–41. doi: 10.5281/zenodo.176040

132. Huang D, Wegierek P, ŻYŁA D *et al.* The oldest aphid of the family Oviparosiphidae (Hemiptera: Aphidoidea) from the Middle Jurassic of China. *European Journal of Entomology*. 2015; **112**(1): 187–192. doi: 10.14411/eje.2015.013

133. Lu Y, Yao Y, Ren D. Two new genera and species of fossil true bugs (Hemiptera: Heteroptera: Pachymeridiidae) from northeastern China. *Zootaxa*. 2011; **2835**(1): 41–52. doi: 10.11646/zootaxa.2835.1.3

134. Yunzhi YAO, Wanzhi CAI, Dong REN. New Jurassic Fossil True Bugs of the Pachymeridiidae (Hemiptera: Pentatomomorpha) from Northeast China. *Acta Geologica Sinica - English Edition*. 2010; **82**(1): 35–47. doi: 10.1111/j.1755-6724.2008.tb00322.x

135. Yao Y, Cai W, Ren D. Sinopachymeridium popovi gen. and sp. nov. – a new fossil true bug (Heteroptera: Pachymeridiidae) from the Middle Jurassic of Inner Mongolia, China. *Annales Zoologici*. 2006; **56**: 753–756. doi: 10.3161/000345406779508552

136. Wang B, Zhang H, Fang Y. Middle Jurassic Palaeontinidae (Insecta, Hemiptera) from Daohugou of China. *Alavesia*. 2007; **1**: 89–104.

137. Wang Y, Ren D. New Fossil Palaeontinids from the Middle Jurassic of Daohugou, Inner Mongolia, China (Insecta, Hemiptera). *Acta Geologica Sinica - English Edition*. 2009; **83**(1): 33–38. doi: 10.1111/j.1755-6724.2009.00004.x

138. Wang Y, Ren D, Shih C. New discovery of Palaeontinid fossils from the Middle Jurassic in Daohugou, Inner Mongolia (Homoptera, Palaeontinidae). *Science in China Series D: Earth Sciences*. 2007; **50**(4): 481–486. doi: 10.1007/s11430-007-0029-5

139. Wang B, Zhang H, Fang Y. Gansucossus, a replacement name for Yumenia Hong, 1982 (Insecta, Hemiptera, Palaeontinidae), with description of a new genus. *Zootaxa*. 2006; **1268**(1): 59–68. doi: 10.11646/zootaxa.1268.1.3

140. Wang Y, Liang J, Shih C *et al.* Revision ofEoiocossus(Insecta, Hemiptera, Palaeontinidae) from the Middle Jurassic of Northeastern China. *Alcheringa: An Australasian Journal of Palaeontology*. 2021; **45**(3): 329–334. doi: 10.1080/03115518.2021.1964038

141. Wang B, Zhang H, Fang Y *et al.* A new genus and species of Palaeontinidae (Insecta: Hemiptera) from the Middle Jurassic of Daohugou, China. *Annales Zoologici*. 2006; **53**: 757–762. doi: 10.3161/000345406779508606

142. Wang Y, Ren D. Middle Jurassic Pseudocossus fossils from Daohugou, Inner Mongolia in China (Homoptera, Palaeontinidae). *Acta Zootaxonomica Sinica*. 2006; **31**: 289–293.

143. Bo W, Haichun Z, Yan F *et al.* A revision of Palaeontinidae (Insecta: Hemiptera: Cicadomorpha) from the Jurassic of China with descriptions of new taxa and new combinations. *Geological Journal*. 2007; **43**(1): 1–18. doi: 10.1002/gj.1092

144. Wang B, Zhang H, Szwedo J. Jurassic Palaeontinidae from China and the higher systematics of Palaeontinoidea (Insecta: Hemiptera: Cicadomorpha). *Palaeontology*. 2009; **52**: 53–64. doi: 10.1111/j.1475-4983.2008.00826.x

145. Wang B, Zhang H-C, Fang Y. Some Jurassic Palaeontinidae (Insecta, Hemiptera) from Daohugou, Inner Mongolia, China. *Palaeoworld*. 2006; **15**(1): 115–125. doi: 10.1016/j.palwor.2006.03.006

146. Becker-Migdisova EE, Wootton RD. Novye Paleontinoidy Azii Paleontologicheskii Zhurnal. 63–79.

147. Wang BO, Zhang H, Fang YAN. Palaeontinodes reshuitangensis, a new species of Palaeontinidae (Hemiptera, Cicadomorpha) from the Middle Jurassic of Reshuitang and Daohugou of China. *Zootaxa*. 2007; **1500**(1): 61–68. doi: 10.11646/zootaxa.1500.1.3

148. Ying W, Dong R, ChungKun S. Discovery of Middle Jurassic palaeontinids from Inner Mongolia, China (Homoptera: Palaeontinidae). *Progress in Natural Science*. 2007; **17**(1): 112–116. doi: 10.1080/10020070612331343234

149. Chen J, Zhang H, Wang B *et al.* New Jurassic Sinopalaeocossus and related genera with notes on their evolutionary implications (Hemiptera, Palaeontinidae). *Insect Systematics & Evolution*. 2016; **47**(2): 113–129. doi: 10.1163/1876312x-47022136

150. Wang Y, Wang L, Ren D. Revision of genera Quadraticossus, Martynovocossus and Fletcheriana (Insecta, Hemiptera) from the Middle Jurassic of China with description of a new species. *Zootaxa*. 2008; **1855**: 56–64. doi: 10.5281/zenodo.183524

151. Wang Y, Ren D. Two new genera of fossil palaeontinids from the Middle Jurassic in Daohugou, Inner Mongolia, China (Hemiptera, Palaeontinidae). *Zootaxa*. 2007; **1390**: 41–49. doi: 10.5281/zenodo.175235

152. Wang B, Zhang H, Fang Y *et al.* Revision of the genus Sinopalaeocossus Hong (Hemiptera: Palaeontinidae), with description of a new species from the Middle Jurassic of China. *Zootaxa*. 2006; **1349**: 37–45. doi: 10.5281/zenodo.174498

153. Wang Y, Ren D. Revision of the genus Suljuktocossus Becker-Migdisova, 1949 (Hemiptera, Palaeontinidae), with description of a new species from Daohugou, Inner Mongolia, China. *Zootaxa*. 2007; **1576**(1): 57–62. doi: 10.11646/zootaxa.1576.1.4

154. Wang Y, Shih C, Szwedo J *et al.* New fossil palaeontinids (Hemiptera, Cicadomorpha, Palaeontinidae) from the Middle Jurassic of Daohugou, China. *Alcheringa: An Australasian Journal of Palaeontology*. 2012; **37**(1): 19–30. doi: 10.1080/03115518.2012.690972

155. Chen J, Wang B, Zhang H *et al.* New fossil Procercopidae (Hemiptera: Cicadomorpha) from the Middle Jurassic of Daohugou, Inner Mongolia, China. *European Journal of Entomology*. 2015; **112**(2): 373–380. doi: 10.14411/eje.2015.044

156. Nascimento FS, Li S, Shih C *et al.* Forever Love: The Hitherto Earliest Record of Copulating Insects from the Middle Jurassic of China. *PLoS ONE*. 2013; **8**(11). doi: 10.1371/journal.pone.0078188

157. Fu Y, Huang D, Engel MS. A new species of the extinct family Procercopidae (Hemiptera: Cercopoidea) from the Jurassic of northeastern China. *Palaeoentomology*. 2018; **1**(1): 51–57. doi: 10.11646/palaeoentomology.1.1.7

158. Wang B, Zhang H. A remarkable new genus of Procercopidae (Hemiptera: Cercopoidea) from the Middle Jurassic of China. *Comptes Rendus Palevol*. 2009; **8**(4): 389–394. doi: 10.1016/j.crpv.2009.01.003

159. Chen J, Zhang H, Wang B *et al.* High variability in tegminal venation of primitive cercopoids (Hemiptera: Cicadomorpha), as implied by the new discovery of fossils from the Middle Jurassic of China. *Entomological Science*. 2015; **18**(2): 147–152. doi: 10.1111/ens.12103

160. Dong Q, Yao Y, Ren D. New fossil Progonocimicidae (Hemiptera: Coleorrhyncha: Progonocimicoidea) from the Upper Mesozoic of northeastern China, with a phylogeny of Coleorrhyncha. *Systematic Entomology*. 2014; **39**(4): 773–782. doi: 10.1111/syen.12085

161. Dong Q-p, Yao Y-z, Ren D. A new species of Progonocimicidae (Hemiptera, Coleorrhyncha) from the Middle Jurassic of China. *Alcheringa: An Australasian Journal of Palaeontology*. 2013; **37**(1): 31–37. doi: 10.1080/03115518.2012.701486

162. Wang B, Szwedo J, Zhang H. Jurassic Progonocimicidae (Hemiptera) from China and phylogenetic evolution of Coleorrhyncha. *Science in China Series D: Earth Sciences*. 2009; **52**(12): 1953–1961. doi: 10.1007/s11430-009-0160-6

163. Jiang J, Huang D. New species of Cicadocoris (Hemiptera: Coleorrhyncha: Progonocimicidae) from mid-Jurassic deposits in northeastern China. *European Journal of Entomology*. 2017; **114**: 355–364. doi: 10.14411/eje.2017.045

164. Dong Q, Yao Y, Ren D. A new species of Progonocimicidae (Hemiptera: Coleorrhyncha) from northeastern China. *Zootaxa*. 2012; **3495**(1): 73–78. doi: 10.11646/zootaxa.3495.1.4

165. Yang G, Yao Y, Ren D. A new species of Protopsyllidiidae (Hemiptera, Sternorrhyncha) from the Middle Jurassic of China. *Zootaxa*. 2013; **3274** 36–42.

166. Yang G, Yao Y, Ren D. Poljanka strigosa, a new species of Protopsyllidiidae (Hemiptera, Sternorrhyncha) from the Middle Jurassic of China. *Alcheringa: An Australasian Journal of Palaeontology*. 2013; **37**(1): 125–130. doi: 10.1080/03115518.2012.715325

167. Yao Y, Cai W, Ren D. New fossil rhopalids (Heteroptera: Coreoidea) from the Middle Jurassic of Inner Mongolia, China. *Zootaxa*. 2006; **1269**(1384): 41–58. doi: 10.5281/zenodo.173217

168. Yao Y, Cai W, Ren D. The first discovery of fossil rhopalids (Heteroptera: Coreoidea) from Middle Jurassic of Inner Mongolia, China. *Zootaxa*. 2006; **1269**(1): 57–68. doi: 10.11646/zootaxa.1269.1.5

169. Wang BO, Szwedo J, Zhang H. New Jurassic Cercopoidea from China and their evolutionary significance (Insecta: Hemiptera). *Palaeontology*. 2013; **55**(6): 1223–1243. doi: 10.1111/j.1475-4983.2012.01185.x

170. Fu Y, Cai C, Huang D. A new fossil sinoalid species from the Middle Jurassic Daohugou beds (Insecta: Hemiptera: Cercopoidea). *Alcheringa: An Australasian Journal of Palaeontology*. 2017; **42**(1): 94–100. doi: 10.1080/03115518.2017.1374458

171. Chen J, Zheng Y, Wei G *et al.* New data on Jurassic Sinoalidae from northeastern China (Insecta, Hemiptera). *Journal of Paleontology*. 2017; **91**(5): 994–1000. doi: 10.1017/jpa.2017.27

172. Fu Y-Z, Huang D-Y. New fossil genus and species of Sinoalidae (Hemiptera: Cercopoidea) from the Middle to Upper Jurassic deposits in northeastern China. *European Journal of Entomology*. 2018; **115**: 127–133. doi: 10.14411/eje.2018.011

173. Huang D, Nel A. A New Middle Jurassic Aphid Family (Insecta: Hemiptera: Sternorrhyncha: Sinojuraphididae Fam. Nov.) from Inner Mongolia, China. *Palaeontology*. 2008; **51**(3): 715–719. doi: 10.1111/j.1475-4983.2008.00773.x

174. Liu X-H, Li Y, Yao Y-Z *et al.* A hairy-bodied tettigarctid (Hemiptera: Cicadoidea) from the latest Middle Jurassic of northeast China. *Alcheringa: An Australasian Journal of Palaeontology*. 2016; **40**(3): 383–389. doi: 10.1080/03115518.2016.1145390

175. Chen J, Zheng Y, Wang X. A new genus and species of Tettigarctidae from the Mesozoic of northeastern China (Insecta, Hemiptera, Cicadoidea). *ZooKeys*. 2016; **632**: 47–55. doi: 10.3897/zookeys.632.10076

176. Chen J, Zhang H, Wang B *et al.* New Jurassic tettigarctid cicadas from China with a novel example of disruptive coloration. *Acta Palaeontologica Polonica*. 2016; **61**: 853–862. doi: 10.4202/app.00238.2015

177. Wang B, Zhang H. Tettigarctidae (Insecta: Hemiptera: Cicadoidea) from the Middle Jurassic of Inner Mongolia, China. *Geobios*. 2009; **42**(2): 243–253. doi: 10.1016/j.geobios.2008.09.003

178. Li S, Wang Y, Ren D *et al.* Revision of the genusSunotettigarctaHong, 1983 (Hemiptera, Tettigarctidae), with a new species from Daohugou, Inner Mongolia, China. *Alcheringa: An Australasian Journal of Palaeontology*. 2012; **36**(4): 501–507. doi: 10.1080/03115518.2012.680722

179. Hou W, Yao Y, Zhang W *et al.* The earliest fossil flower bugs (Heteroptera: Cimicomorpha: Cimicoidea: Vetanthocoridae) from the Middle Jurassic of Inner Mongolia, China. *European Journal of Entomology*. 2012; **109**(2): 281–288. doi: 10.14411/eje.2012.036

180. Wang M, Wang C, Shih C *et al.* Two new species of Prolyda from the Middle Jurassic of China (Hymenoptera, Pamphilioidea). *ZooKeys*. 2016; **569**: 71–80. doi: 10.3897/zookeys.569.7249

181. Wang T, Li L, Rasnitsyn AP *et al.* Anomopterellidae Restored, with Two New Genera and Its Phylogeny in Evanioidea (Hymenoptera). *PLoS ONE*. 2013; **8**(12). doi: 10.1371/journal.pone.0082587

182. Li L, Shih C, Ren D. Revision of Anomopterella Rasnitsyn, 1975 (Insecta, Hymenoptera, Anomopterellidae) with two new Middle Jurassic species from northeastern China. *Geologica Carpathica*. 2014; **65**(5): 368–377. doi: 10.2478/geoca-2014-0025

183. Zhang H, Rasnitsyn AP. Middle Jurassic Praeaulacidae (Insecta: Hymenoptera: Evanioidea) of Inner Mongolia and Kazakhstan. *Journal of Systematic Palaeontology*. 2008; **6**(4): 463–487. doi: 10.1017/s1477201907002428

184. Rasnitsyn AP. Hymenoptera Apocrita of Mesozoic *Trudy Paleontologicheskogo Instituta*. 1975; **147**: 1–134. doi: 10.5281/ZENODO.24283

185. Rasnitsyn A, Zhang H. A new family, Daohugoidae fam. n, of siricomorph hymenopteran (Hymenoptera=Vespida) from the Middle Jurassic of Daohugou in Inner Mongolia (China). *Proceedings of the Russian Entomological Society*. 2004; **75**: 12–16.

186. Rasnttsyn AP, Haichun Z. Early Evolution of Apocrita (Insecta, Hymenoptera) as Indicated by New Findings in the Middle Jurassic of Daohugou, Northeast China. *Acta Geologica Sinica - English Edition*. 2010; **84**(4): 834–873. doi: 10.1111/j.1755-6724.2010.00254.x

187. Zhang Q, Zhang H-C, Rasnitsyn AP *et al.* New Ephialtitidae (Insecta: Hymenoptera) from the Jurassic Daohugou Beds of Inner Mongolia, China. *Palaeoworld*. 2014; **23**(3-4): 276–284. doi: 10.1016/j.palwor.2014.11.001

188. Li L, Shih C, Ren D. Two New Wasps (Hymenoptera: Stephanoidea: Ephialtitidae) from the Middle Jurassic of China. *Acta Geologica Sinica - English Edition*. 2013; **87**(6): 1486–1494. doi: 10.1111/1755-6724.12152

189. Li L, Shih C. Two new fossil wasps (Insecta: Hymenoptera: Apocrita) from northeastern China. *Journal of Natural History*. 2014; **49**(13-14): 829–840. doi: 10.1080/00222933.2014.953223

190. Li L, Shih C, Rasnitsyn AP *et al.* New fossil ephialtitids elucidating the origin and transformation of the propodeal-metasomal articulation in Apocrita (Hymenoptera). *BMC Evolutionary Biology*. 2015; **15**(1): 1–17. doi: 10.1186/s12862-015-0317-1

191. Ding M, Zheng D, Zhang Q *et al.* A new species of Ephialtitidae (Insecta: Hymenoptera: Stephanoidea) from the Middle Jurassic of Inner Mongolia, China. *Acta Palaeontologica Sinica*. 2013; **52**: 51–56.

192. Wang M, Li L, Shih C. New fossil wasps (Hymenoptera, Apocrita) from the Middle Jurassic of China. *Insect Systematics & Evolution*. 2015; **46**(5): 471–484. doi: 10.1163/1876312x-45032127

193. Shih C, Feng H, Ren D. New Fossil Heloridae and Mesoserphidae Wasps (Insecta, Hymenoptera, Proctotrupoidea) from the Middle Jurassic of China. *Annals of the Entomological Society of America*. 2011; **104**(6): 1334–1348. doi: 10.1603/an10194

194. Dong R, Shi X, Zhao Y *et al.* Two new species of Archaeohelorus (Hymenoptera, Proctotrupoidea, Heloridae) from the Middle Jurassic of China. *ZooKeys*. 2014; **369**: 49–59. doi: 10.3897/zookeys.369.6561

195. Rasnitsyn AP. *Proiskhozdenie ivolyutsiya nizshikh pereponchatokrylykh. (Nauka, 1969)*1980.

196. Shih MJH, Li L, Ren D. Application of geometric morphometric analyses to confirm two new species of Karatavitidae (Hymenoptera: Karatavitoidea) from northeastern China. *Alcheringa: An Australasian Journal of Palaeontology*. 2017; **41**(4): 499–508. doi: 10.1080/03115518.2017.1316872

197. Ansorge J, Rasnitsyn A, Zhang H. Ancestry of the orussoid wasps, with description of three new genera and species of Karatavitidae (Hymenoptera = Vespida: Karatavitoidea stat. nov.). *Insect Systematics & Evolution*. 2006; **37**(2): 179–190. doi: 10.1163/187631206788831137

198. Wang MEI, Rasnitsyn AP, Shih C *et al.* New fossils from China elucidating the phylogeny of Praesiricidae (Insecta: Hymenoptera). *Systematic Entomology*. 2015; **41**(1): 41–55. doi: 10.1111/syen.12142

199. Li L, Rasnitsyn AP, Shih C *et al.* The Mesozoic family Mesoserphidae and its phylogeny (Hymenoptera: Apocrita: Proctotrupoidea). *Journal of Systematic Palaeontology*. 2016; **15**(8): 617–639. doi: 10.1080/14772019.2016.1217949

200. Zheng Y, Chen J. A new mesoserphid wasp from the Middle Jurassic of northeastern China (Hymenoptera, Proctotrupoidea). *European Journal of Taxonomy*. 2017; **379**(379): 1–8. doi: 10.5852/ejt.2017.379

201. Wang M, Rasnitsyn AP, Yang Z *et al.* Mirolydidae, a new family of Jurassic pamphilioid sawfly (Hymenoptera) highlighting mosaic evolution of lower Hymenoptera. *Scientific Reports*. 2017; **7**(1): 1–9. doi: 10.1038/srep43944

202. Li L, Shih C, Ren D. Two new species ofNevania(Hymenoptera: Evanioidea: Praeaulacidae: Nevaniinae) from the Middle Jurassic of China. *Alcheringa: An Australasian Journal of Palaeontology*. 2013; **38**(1): 140–147. doi: 10.1080/03115518.2014.843376

203. Rasnitsyn A, Zhang H. Nevaniinae subfam. n., a new fossil taxon (Insecta: Hymenoptera: Evanioidea: Praeaulacidae) from the Middle Jurassic of Daohugou in Inner Mongolia, China. *Insect Systematics & Evolution*. 2007; **38**(2): 149–166. doi: 10.1163/187631207788783987

204. Wang M, Shih C, Ren D *et al.* A new fossil genus in Pamphiliidae (Hymenoptera) from China. *Alcheringa: An Australasian Journal of Palaeontology*. 2014; **38**(3): 391–397. doi: 10.1080/03115518.2014.884366

205. Shih C, Liu C, Ren D. The Earliest Fossil Record of Pelecinid Wasps (Inseta: Hymenoptera: Proctotrupoidea: Pelecinidae) from Inner Mongolia, China. *Annals of the Entomological Society of America*. 2009; **102**(1): 20–38. doi: 10.1603/008.102.0103

206. Li L, Shih C, Ren D. New fossil Praeaulacinae wasps (Insect: Hymenoptera: Evanioidea: Praeaulacidae) from the Middle Jurassic of China. *Zootaxa*. 2014; **3814**(3): 432–442. doi: 10.11646/zootaxa.3814.3.10

207. Li L, Rasnitsyn AP, Shih C *et al.* Phylogeny of Evanioidea (Hymenoptera, Apocrita), with descriptions of new Mesozoic species from China and Myanmar. *Systematic Entomology*. 2018; **43**(4): 810–842. doi: 10.1111/syen.12315

208. Gao T, Ren D, Shih C. Abrotoxyela gen. nov. (Insecta, Hymenoptera, Xyelidae) from the Middle Jurassic of Inner Mongolia, China. *Zootaxa*. 2009; **2094**: 52–59. doi: 10.5281/zenodo.187569

209. Wang M, Rasnitsyn AP, Ren D. Two New Fossil Sawflies (Hymenoptera, Xyelidae, Xyelinae) from the Middle Jurassic of China. *Acta Geologica Sinica - English Edition*. 2014; **88**(4): 1027–1033. doi: 10.1111/1755-6724.12269

210. Wang M, Shih C, Ren D. Platyxyela gen. nov. (Hymenoptera, Xyelidae, Macroxyelinae) from the Middle Jurassic of China. *Zootaxa*. 2012; **3456**(1): 82–88. doi: 10.11646/zootaxa.3456.1.4

211. Gao T, Ren D, Shih C. The First Xyelotomidae (Hymenoptera) from the Middle Jurassic in China. *Annals of the Entomological Society of America*. 2009; **102**(4): 588–596. doi: 10.1603/008.102.0402

212. Gao T, Shih C, Engel MS *et al.* A new xyelotomid (Hymenoptera) from the Middle Jurassic of China displaying enigmatic venational asymmetry. *BMC Evolutionary Biology*. 2016; **16**(1): 1–7. doi: 10.1186/s12862-016-0730-0

213. Wang M, Rasnitsyn AP, Li H *et al.* Phylogenetic analyses elucidate the inter‐relationships of Pamphilioidea (Hymenoptera, Symphyta). *Cladistics*. 2015; **32**(3): 239–260. doi: 10.1111/cla.12129

214. Rasnitsyn AP, Zhang H, Wang BO. Bizarre fossil insects: web‐spinning sawflies of the genus Ferganolyda (Vespida, Pamphilioidea) from the Middle Jurassic of Daohugou, Inner Mongolia, China. *Palaeontology*. 2006; **49**(4): 907–916. doi: 10.1111/j.1475-4983.2006.00574.x

215. Wang M, Rasnitsyn AP, Shih C *et al.* New fossil records of bizarre Ferganolyda (Hymenoptera: Xyelydidae) from the Middle Jurassic of China. *Alcheringa: An Australasian Journal of Palaeontology*. 2014; **39**(1): 99–108. doi: 10.1080/03115518.2015.958286

216. Frayer D, Zhang W, Shih C *et al.* New Fossil Lepidoptera (Insecta: Amphiesmenoptera) from the Middle Jurassic Jiulongshan Formation of Northeastern China. *PLoS ONE*. 2013; **8**(11). doi: 10.1371/journal.pone.0079500

217. Zhang W-T, Shih C-K, Labandeira CC *et al.* A new taxon of a primitive moth (Insecta: Lepidoptera: Eolepidopterigidae) from the latest Middle Jurassic of northeastern China. *Journal of Paleontology*. 2015; **89**(4): 617–621. doi: 10.1017/jpa.2015.39

218. Diying H, Nel A, Minet J. A New Family of Moths from the Middle Jurassic (Insecta: Lepidoptera). *Acta Geologica Sinica - English Edition*. 2010; **84**(4): 874–885. doi: 10.1111/j.1755-6724.2010.00233.x

219. Huang D-y, Nel A, Zompro O *et al.* Mantophasmatodea now in the Jurassic. *Naturwissenschaften*. 2008; **95**(10): 947–952. doi: 10.1007/s00114-008-0412-x

220. Carrier D, Wang Q, Shih C *et al.* The Earliest Case of Extreme Sexual Display with Exaggerated Male Organs by Two Middle Jurassic Mecopterans. *PLoS ONE*. 2013; **8**(8). doi: 10.1371/journal.pone.0071378

221. Liu S, Shih C, Bashkuev A *et al.* New Jurassic Hangingflies (Insecta: Mecoptera: Bittacidae) from Inner Mongolia, China. *Zootaxa*. 2016; **4067**(1): 65–78. doi: 10.11646/zootaxa.4067.1.5

222. Li Y, Ren D. Middle Jurassic Bittacidae (Insecta, Mecoptera) from Daohugou, Inner Mongolia, China. *Acta Zootaxonomica Sinica*. 2009; **34**: 560–567.

223. Liu S, Shih C, Ren D. Four new species of hangingflies (Insecta, Mecoptera, Bittacidae) from the Middle Jurassic of northeastern China. *ZooKeys*. 2014; **466**: 77–94. doi: 10.3897/zookeys.466.8047

224. Yang X, Ren D, Shih C. New fossil hangingflies (Mecoptera, Raptipeda, Bittacidae) from the Middle Jurassic to Early Cretaceous of Northeastern China. *Geodiversitas*. 2012; **34**(4): 785–799. doi: 10.5252/g2012n4a4

225. Li Y, Ren D, Shih C. Two Middle Jurassic hanging-flies (Insecta: Mecoptera: Bittacidae) from Northeast China. *Zootaxa*. 2008; **1929**(1): 38–46. doi: 10.11646/zootaxa.1929.1.2

226. Petrulevicius JF, Huang D, Ren D. A new hangingfly (Insecta: Mecoptera: Bittacidae) from the Middle Jurassic of Inner Mongolia, China. *African Invertebrates*. 2007; **48**(1): 145–152. doi: <https://hdl.handle.net/10520/EJC84585>

227. Kopeć K, Krzemiński W, Soszyńska-Maj A *et al.* A new species ofOrthobittacus(Mecoptera, Bittacidae) from the Middle Jurassic of Daohugou, Inner Mongolia (China). *Earth and Environmental Science Transactions of the Royal Society of Edinburgh*. 2017; **107**(2-3): 157–162. doi: 10.1017/s1755691017000251

228. Yang X-G, Shih C-K, Ren D *et al.* New Middle Jurassic hangingflies (Insecta: Mecoptera) from Inner Mongolia, China. *Alcheringa: An Australasian Journal of Palaeontology*. 2012; **36**(2): 195–201. doi: 10.1080/03115518.2012.622143

229. Dong R, Qiao X, Shih C *et al.* Fossils from the Middle Jurassic of China shed light on morphology of Choristopsychidae (Insecta, Mecoptera). *ZooKeys*. 2013; **318**: 91–111. doi: 10.3897/zookeys.318.5226

230. Yang X, Shih C, Ren D. New fossil hangingflies (Insecta: Mecoptera: Raptipedia: Cimbrophlebiidae) from the Middle Jurassic of Inner Mongolia, China. *Palaeontology*. 2013; **56**(4): 711–726. doi: 10.1111/pala.12009

231. Xiao Z, Chungkun S, Yunyun Z *et al.* New Species of Cimbrophlebiidae (Insecta: Mecoptera) from the Middle Jurassic of Northeastern China. *Acta Geologica Sinica - English Edition*. 2015; **89**(5): 1482–1496. doi: 10.1111/1755-6724.12559

232. Wang Y, Labandeira CC, Shih C *et al.* Jurassic mimicry between a hangingfly and a ginkgo from China. *Proceedings of the National Academy of Sciences*. 2012; **109**(50): 20514–20519. doi: 10.1073/pnas.1205517109

233. Zhang J, Shih C, Petrulevičius JF *et al.* A new fossil eomeropid (Insecta, Mecoptera) from the Jiulongshan Formation, Inner Mongolia, China. *Zoosystema*. 2011; **33**(4): 443–450. doi: 10.5252/z2011n4a2

234. Ren D, Shih C. The first discovery of fossil eomeropids from China (Insecta, Mecoptera). *Acta Zootaxonomica Sinica*. 2005; **30**: 275–280. doi: 10.3969/j.issn.1000-0739.2005.02.009

235. Li L, Shih C, Wang C *et al.* A New Fossil Scorpionfly (Insecta: Mecoptera: Holcorpidae) with Extremely Elongate Male Genitalia from Northeastern China. *Acta Geologica Sinica - English Edition*. 2017; **91**(3): 797–805. doi: 10.1111/1755-6724.13310

236. ChungKun S, Xiao Q, Labandeira CC *et al.* A New Mesopsychid (Mecoptera) from the Middle Jurassic of Northeastern China. *Acta Geologica Sinica - English Edition*. 2013; **87**(5): 1235–1241. doi: 10.1111/1755-6724.12124

237. Dong R, Labandeira CC, ChungKun S. New Mesozoic Mesopsychidae (Mecoptera) from Northeastern China. *Acta Geologica Sinica - English Edition*. 2010; **84**(4): 720–731. doi: 10.1111/j.1755-6724.2010.00244.x

238. Lin X, Shih MJH, Labandeira CC *et al.* New data from the Middle Jurassic of China shed light on the phylogeny and origin of the proboscis in the Mesopsychidae (Insecta: Mecoptera). *BMC Evolutionary Biology*. 2016; **16**(1): 1–22. doi: 10.1186/s12862-015-0575-y

239. Cao Y, Shih C, Bashkuev A *et al.* Revision and two new species ofItaphlebia(Nannochoristidae: Mecoptera) from the Middle Jurassic of Inner Mongolia, China. *Alcheringa: An Australasian Journal of Palaeontology*. 2015; **40**(1): 24–33. doi: 10.1080/03115518.2015.1079692

240. Cao Y, Lin X, Shih C *et al.* ﻿Two new species of Itaphlebia (Insecta, Mecoptera, Nannochoristidae) from the late Middle Jurassic of China. *ZooKeys*. 2022; **1108**: 175–188. doi: 10.3897/zookeys.1108.85378

241. Petrulevičius JF, Ren D. A new species of Orthophlebiidae" (Insecta: Mecoptera) from the Middle Jurassic of Inner Mongolia, China. *Revue de Palobiologie*. 2012; **11**: 311–315.

242. Soszyńska-Maj A, Krzemiński W, Kopeć K *et al.* Large Jurassic Scorpionflies Belonging to a New Subfamily of the Family Orthophlebiidae (Mecoptera). *Annales Zoologici*. 2018; **68**(1): 85–92. doi: 10.3161/00034541anz2018.68.1.004

243. Qiao X, Shih C, Ren D. Two new Middle Jurassic species of orthophlebiids (Insecta: Mecoptera) from Inner Mongolia, China. *Alcheringa: An Australasian Journal of Palaeontology*. 2012; **36**(4): 469–475. doi: 10.1080/03115518.2012.671689

244. Dong R, Ding H, Shih C *et al.* The earliest fossil record of Panorpidae (Mecoptera) from the Middle Jurassic of China. *ZooKeys*. 2014; **431**: 79–92. doi: 10.3897/zookeys.431.7561

245. Ren D, Shih C, Labandeira CC. New Jurassic Pseudopolycentropodids from China (Insecta: Mecoptera). *Acta Geologica Sinica - English Edition*. 2010; **84**(1): 22–30. doi: 10.1111/j.1755-6724.2010.00166.x

246. Ren D, Labandeira CC, Santiago-Blay JA *et al.* A Probable Pollination Mode Before Angiosperms: Eurasian, Long-Proboscid Scorpionflies. *Science*. 2009; **326**(5954): 840–847. doi: 10.1126/science.1178338

247. Ren D, Shih C, Yang X *et al.* A new long-proboscid genus of Pseudopolycentropodidae (Mecoptera) from the Middle Jurassic of China and its plant-host specializations. *ZooKeys*. 2011; **130**: 281–297. doi: 10.3897/zookeys.130.1641

248. Moreau CS, Liu X, Wang Y *et al.* Early Evolution and Historical Biogeography of Fishflies (Megaloptera: Chauliodinae): Implications from a Phylogeny Combining Fossil and Extant Taxa. *PLoS ONE*. 2012; **7**(7). doi: 10.1371/journal.pone.0040345

249. Wang B, Zhang H. Earliest evidence of fishflies (Megaloptera: Corydalidae): an exquisitely preserved larva from the Middle Jurassic of China. *Journal of Paleontology*. 2016; **84**(4): 774–780. doi: 10.1666/09-162.1

250. Engel MS, Huang D, Lin Q. A new genus and species of Aetheogrammatidae from the Jurassic of Inner Mongolia, China (Neuroptera). *Journal of the Kansas Entomological Society*. 2011; **84**: 315–319. doi: 10.2307/41353894

251. Yang Q, Makarkin V, Ren D. Two new species of Sinosmylites Hong (Neuroptera, Berothidae) from the Middle Jurassic of China, with notes on Mesoberothidae. *ZooKeys*. 2011; **130**: 199–215. doi: 10.3897/zookeys.130.1418

252. Khramov AV, Liu Q, Zhang H *et al.* Early green lacewings (Insecta: Neuroptera: Chrysopidae) from the Jurassic of China and Kazakhstan. *Papers in Palaeontology*. 2015; **2**(1): 25–39. doi: 10.1002/spp2.1024

253. Shi C, Wang Y, Yang Q *et al.* Chorilingia(Neuroptera: Grammolingiidae): a new genus of lacewings with four species from the Middle Jurassic of Inner Mongolia, China. *Alcheringa: An Australasian Journal of Palaeontology*. 2012; **36**(3): 309–318. doi: 10.1080/03115518.2012.644994

254. Shi C, Wang Y, Ren D. New species ofGrammolingiaRen, 2002 from the Middle Jurassic of Inner Mongolia, China (Neuroptera: Grammolingiidae). *Fossil Record*. 2013; **16**(2): 171–178. doi: 10.5194/fr-16-171-2013

255. Dong R. A New Lacewing Family (Neuroptera)from the Middle Jurassic of Inner Mongolia, China. *Insect Science*. 2008; **9**(4): 53–67. doi: 10.1111/j.1744-7917.2002.tb00172.x

256. Shi C, Yang Q, Ren D. Two New Fossil Lacewing Species from the Middle Jurassic of Inner Mongolia, China (Neuroptera: Grammolingiidae). *Acta Geologica Sinica - English Edition*. 2011; **85**(2): 482–489. doi: 10.1111/j.1755-6724.2011.00416.x

257. Liu Y, Shi C, Ren D. A new lacewing (Insecta: Neuroptera: Grammolingiidae) from the Middle Jurassic of Inner Mongolia, China. *Zootaxa*. 2011; **2897**(1): 51–56. doi: 10.11646/zootaxa.2897.1.5

258. Zheng B, Ren D, Wang Y. Earliest true moth lacewing from the Middle Jurassic of Inner Mongolia, China. *Acta Palaeontologica Polonica*. 2016; **61**: 847–851. doi: 10.4202/app.00259.2016

259. Yang Q, Wang Y, Labandeira CC *et al.* Mesozoic lacewings from China provide phylogenetic insight into evolution of the Kalligrammatidae (Neuroptera). *BMC Evolutionary Biology*. 2014; **14**(1): 1–30. doi: 10.1186/1471-2148-14-126

260. Yang Q, Makarkin VN, Ren D. Two interesting new genera of Kalligrammatidae (Neuroptera) from the Middle Jurassic of Daohugou, China. *Zootaxa*. 2011; **2873**(1): 60–68. doi: 10.11646/zootaxa.2873.1.5

261. Liu Q, Zheng D, Zhang Q *et al.* Two new kalligrammatids (Insecta, Neuroptera) from the Middle Jurassic of Daohugou, Inner Mongolia, China. *Alcheringa: An Australasian Journal of Palaeontology*. 2013; **38**(1): 65–69. doi: 10.1080/03115518.2013.828251

262. Yang Q, Makarkin VN, Ren D. Two New Species ofKalligrammaWalther (Neuroptera: Kalligrammatidae) from the Middle Jurassic of China. *Annals of the Entomological Society of America*. 2014; **107**(5): 917–925. doi: 10.1603/an14032

263. Liu Q, Khramov AV, Zhang H. A new species ofKalligrammaWalther, 1904 (Insecta, Neuroptera, Kalligrammatidae) from the Middle–Upper Jurassic of Daohugou, Inner Mongolia, China. *Alcheringa: An Australasian Journal of Palaeontology*. 2015; **39**(3): 438–442. doi: 10.1080/03115518.2015.1021564

264. Makarkin VN, Ren D, Yang Q. Two New Species of Kalligrammatidae (Neuroptera) from the Jurassic of China, with Comments on Venational Homologies. *Annals of the Entomological Society of America*. 2009; **102**(6): 964–969. doi: 10.1603/008.102.0606

265. Liu Q, Khramov AV, Zhang H *et al.* Two new species ofKalligrammulaHandlirsch, 1919 (Insecta, Neuroptera, Kalligrammatidae) from the Jurassic of China and Kazakhstan. *Journal of Paleontology*. 2015; **89**(3): 405–410. doi: 10.1017/jpa.2015.25

266. Ren D, Oswald J. A new genus of kalligrammatid lacewings from the Middle Jurassic of China (Neuroptera: Kalligrammatidae) *Stuttgarter Beitrge zur Naturkunde Serie B (Geologie und Palaontologie)*. 2002; **317**: 1–8.

267. Junfeng Z. Kalligrammatid Lacewings from the Upper Jurassic Daohugou Formation in Inner Mongolia, China. *Acta Geologica Sinica - English Edition*. 2010; **77**(2): 141–147. doi: 10.1111/j.1755-6724.2003.tb00556.x

268. Jepson JE, Heads SW, Makarkin VN *et al.* New fossil mantidflies (Insecta: Neuroptera: Mantispidae) from the Mesozoic of north‐eastern China. *Palaeontology*. 2013; **56**(3): 603–613. doi: 10.1111/pala.12005

269. Yang Q, Makarkin VN, Ren D. New fossil Mesochrysopidae (Neuroptera) from the Mesozoic of China. *Zootaxa*. 2012; **3597**(1): 1–14. doi: 10.11646/zootaxa.3597.1.1

270. Dong R, Makarkin V, Yang Q *et al.* The presence of the recurrent veinlet in the Middle Jurassic Nymphidae (Neuroptera): a unique character condition in Myrmeleontoidea. *ZooKeys*. 2013; **325**: 1–20. doi: 10.3897/zookeys.325.5453

271. Ren D, Engel MS. A split-footed lacewing and two epiosmylines from the Jurassic of China (Neuroptera). *Annales Zoologici*. 2007; **57**(2): 211–219.

272. Shi C, Makarkin VN, Yang Q *et al.* New species of Nymphites Haase (Neuroptera: Nymphidae) from the Middle Jurassic of China, with a redescription of the type species of the genus *Zootaxa*. 2013; **3700**(3): 393–410. doi: 10.11646/zootaxa.3700.3.4

273. Yang Q, Makarkin VN, Ren D. Remarkable New Genus of Gumillinae (Neuroptera: Osmylidae) from the Jurassic of China. *Annals of the Entomological Society of America*. 2010; **103**(6): 855–859. doi: 10.1603/an10097

274. Khramov AV, Liu Q, Zhang H. Mesozoic diversity of relict subfamily Kempyninae (Neuroptera: Osmylidae). *Historical Biology*. 2017; **31**(7): 938–946. doi: 10.1080/08912963.2017.1411351

275. Makarkin V. A new basal osmylid neuropteran insect from the Middle Jurassic of China linking Osmylidae to the Permian-Triassic Archeosmylidae. *Acta Palaeontologica Polonica*. 2012; **59**: 209–214. doi: 10.4202/app.2011.0018

276. Ren D, Yin J. A new Middle Jurassic species of Epiosmylus from Inner Mongolia, China (Neuroptera: Osmylidae). *Acta Zootaxonomica Sinica*. 2003; **27**: 274–277.

277. Wang Y, Liu Z, Ren D *et al.* A new genus of Protosmylinae from the Middle Jurassic of China (Neuroptera: Osmylidae). *Zootaxa*. 2010; **2480**: 45–83. doi: 10.5281/zenodo.195411

278. Wang Y, Liu Z, Ren D *et al.* New Middle Jurassic Kempynin Osmylid Lacewings from China. *Acta Palaeontologica Polonica*. 2011; **56**(4): 865–869. doi: 10.4202/app.2010.0050

279. Li Q, Ren D, Wang Y. Revision of the gumilline genus Nilionympha with a new species from the Middle Jurassic of China (Neuroptera: Osmylidae). *Zootaxa*. 2018; **4399**(1): 146–150. doi: 10.11646/zootaxa.4399.1.13

280. Wang Y, Liu Z, Ren D. A new fossil lacewing genus from the Middle Jurassic of Inner Mongolia, China (Neuroptera: Osmylidae). *Zootaxa*. 2009; **2034**: 65–68. doi: 10.5281/zenodo.186346

281. Wang Y, Liu Z, Ren D. A New Fossil Lacewing Genus and Species from the Middle Jurassic of Inner Mongolia, China. *Acta Palaeontologica Polonica*. 2009; **54**(3): 557–560. doi: 10.4202/app.2008.0040

282. Peng Y, Makarkin VN, Ren D. Diverse new Middle Jurassic Osmylopsychopidae (Neuroptera) from China shed light on the classification of psychopsoids. *Journal of Systematic Palaeontology*. 2015; **14**(4): 261–295. doi: 10.1080/14772019.2015.1042080

283. Yang Q, Makarkin VN, Ren D. A new genus of the family Panfiloviidae (Insecta, Neuroptera) from the Middle Jurassic of China. *Palaeontology*. 2013; **56**(1): 49–59. doi: 10.1111/j.1475-4983.2012.01157.x

284. O'Grady P, Yang Q, Makarkin VN *et al.* A Remarkable New Family of Jurassic Insects (Neuroptera) with Primitive Wing Venation and Its Phylogenetic Position in Neuropterida. *PLoS ONE*. 2012; **7**(9). doi: 10.1371/journal.pone.0044762

285. Ren D, Engel MS, Lu W. New giant lacewings from the Middle Jurassic of Inner Mongolia, China (Neuroptera: Polystoechotidae). *Journal of the Kansas Entomological Society*. 2002; **75**(3): 188–193.

286. Peng Y, Makarkin VN, Yang Q *et al.* A new silky lacewing (Neuroptera: Psychopsidae) from the Middle Jurassic of Inner Mongolia, China. *Zootaxa*. 2010; **2663**(1): 59–67. doi: 10.11646/zootaxa.2663.1.4

287. Wang Y, Liu Z, Wang X *et al.* Ancient pinnate leaf mimesis among lacewings. *Proceedings of the National Academy of Sciences*. 2010; **107**(37): 16212–16215. doi: 10.1073/pnas.1006460107

288. Liu Q, Zhang H, Wang B *et al.* A new saucrosmylid lacewing (Insecta, Neuroptera) from the Middle Jurassic of Daohugou, Inner Mongolia, China. *Alcheringa: An Australasian Journal of Palaeontology*. 2014; **38**(2): 301–304. doi: 10.1080/03115518.2014.886849

289. Liu Q, Zhang H, Wang B *et al.* A new genus of Saucrosmylinae (Insecta, Neuroptera) from the Middle Jurassic of Daohugou, Inner Mongolia, China. *Zootaxa*. 2013; **3736**(4): 387–391. doi: 10.11646/zootaxa.3736.4.6

290. Ren D, Yin J. New ‘Osmylid-Like’ Fossil Neuroptera from the Middle Jurassic of Inner Mongolia, China. *Journal of the New York Entomological Society*. 2003; **111**(1): 1–11. doi: 10.1664/0028-7199(2003)111[0001:Nofnft]2.0.Co;2

291. Fang H, Ren D, Liu J *et al.* Revision of the lacewing genus Laccosmylus with two new species from the Middle Jurassic of China (Insecta, Neuroptera, Saucrosmylidae). *ZooKeys*. 2018; **790**: 115–126. doi: 10.3897/zookeys.790.28286

292. Zhu C-D, Fang H, Ren D *et al.* Familial Clarification of Saucrosmylidae stat. nov. and New Saucrosmylids from Daohugou, China (Insecta, Neuroptera). *Plos One*. 2015; **10**(10). doi: 10.1371/journal.pone.0141048

293. Li Y, Nel A, Ren D *et al.* A new genus and species of hawker dragonfly of uncertain affinities from the Middle Jurassic of China (Odonata: Aeshnoptera). *Zootaxa*. 2011; **2927**(1): 57–62. doi: 10.11646/zootaxa.2927.1.4

294. Zhang B, Fleck G, Huang D *et al.* New isophlebioid dragonflies (Odonata: Isophlebioptera: Campterophlebiidae) from the Middle Jurassic of China. *Zootaxa*. 2006; **1339**: 51–68. doi: 10.5281/zenodo.174364

295. Li Y, Nel A, Ren D *et al.* A new damsel-dragonfly from the Mesozoic of China with a hook-like male anal angle (Odonata: Isophlebioptera: Campterophlebiidae). *Journal of Natural History*. 2013; **47**(29-30): 1953–1958. doi: 10.1080/00222933.2012.759287

296. PetruleviČIus JF, Huang D, Nel A. A New Genus and Species of Damsel‐Dragonfly (Odonata: Isophlebioidea: Campterophlebiidae) in the Middle Jurassic of Inner Mongolia, China. *Acta Geologica Sinica - English Edition*. 2011; **85**(4): 733–738. doi: 10.1111/j.1755-6724.2011.00478.x

297. Zhang H, Zheng D, Wang B *et al.* The largest known odonate in China: Hsiufua chaoi Zhang et Wang, gen. et sp. nov. from the middle jurassic of inner mongolia. *Chinese Science Bulletin*. 2012; **58**(13): 1579–1584. doi: 10.1007/s11434-012-5567-3

298. Li Y-J, Nel A, Ren D *et al.* Reassessment of the Jurassic damsel-dragonfly genus Karatawia (Odonata: Campterophlebiidae). *Zootaxa*. 2012; **3417**(1): 64–68. doi: 10.11646/zootaxa.3417.1.4

299. Huang D, Cai C, Nel A. New damsel-dragonflies with “calopterygid”-like wing shape from the Middle Jurassic of China (Odonata: Isophlebioidea: Campterophlebiidae). *Geobios*. 2018; **51**(3): 181–186. doi: 10.1016/j.geobios.2018.04.003

300. Nel A, Bechly G, Delclòs X. New and poorly known Mesozoic damsel-dragonflies (Odonata: Isophlebioidea: Campterophlebiidae, Isophlebiidae). *Palaeodiversity*. 2009; **2**: 209–232.

301. Binglan Z, Dong REN, Hong P. New Isophlebioid Dragonflies from the Middle Jurassic of Inner Mongolia, China (Insecta: Odonata: Isophlebioptera: Campterophlebiidae). *Acta Geologica Sinica - English Edition*. 2010; **82**(6): 1104–1114. doi: 10.1111/j.1755-6724.2008.tb00710.x

302. Nel A, Huang D, Lin Q. A new genus of isophlebioid damsel-dragonflies with "calopterygid"-like wing shape from the Middle Jurassic of China (Odonata: Isophlebioidea: Campterophlebiidae). *European Journal of Entomology*. 2007; **105**: 783–787. doi: 10.5281/zenodo.179672

303. Nel A, Huang D. A new family of ‘libelluloid’ dragonflies from the Middle Jurassic of Daohugou, northeastern China (Odonata: Anisoptera: Cavilabiata). *Alcheringa: An Australasian Journal of Palaeontology*. 2015; **39**(4): 525–529. doi: 10.1080/03115518.2015.1050316

304. Li Y, Nel A, Shih C *et al.* The first euthemistid damsel-dragonfly from the Middle Jurassic of China (Odonata, Epiproctophora, Isophlebioptera). *ZooKeys*. 2013; **261**: 41–50. doi: 10.3897/zookeys.261.4371

305. Huang D, Nel A. New fossil damsel–dragonfly clarifies the phylogenetic position of the small Jurassic family Juraheterophlebiidae (Odonata: Epiproctophora). *Alcheringa: An Australasian Journal of Palaeontology*. 2017; **41**(4): 536–542. doi: 10.1080/03115518.2017.1329937

306. Nel A, Azar D, Huang D-Y. A new Middle Jurassic Chinese fossil clarifies the systematic composition of the Heterophlebioptera (Odonata: Trigonoptera). *Alcheringa: An Australasian Journal of Palaeontology*. 2013; **38**(1): 130–134. doi: 10.1080/03115518.2014.843385

307. Huang D-Y, Nel A. Oldest 'libelluloid' dragonfly from the Middle Jurassic of China (Odonata: Anisoptera: Cavilabiata). *Neues Jahrbuch für Geologie und Paläontologie - Abhandlungen*. 2007; **246**(1): 63–68. doi: 10.1127/0077-7749/2007/0246-0063

308. Pinkert S, Nel A, Huang D-Y. A new hawker dragonfly from the Middle Jurassic of China (Odonata: Aeshnoptera). *Comptes Rendus Palevol*. 2017; **16**(4): 378–381. doi: 10.1016/j.crpv.2017.01.006

309. Nel A, Huang D. First Chinese Cymatophlebiidae from the Middle Jurassic of Inner Mongolia (Odonata: Anisoptera: Aeshnoptera). *Palaeodiversity*. 2009; **2**: 199–204.

310. Gu J-J, Montealegre-Z F, Robert D *et al.* Wing stridulation in a Jurassic katydid (Insecta, Orthoptera) produced low-pitched musical calls to attract females. *Proceedings of the National Academy of Sciences*. 2012; **109**(10): 3868–3873. doi: 10.1073/pnas.1118372109

311. Gu J-J, Qiao G-X, Ren D. The first discovery of Cyrtophyllitinae (Orthoptera, Haglidae) from the Middle Jurassic and its morphological implications. *Alcheringa: An Australasian Journal of Palaeontology*. 2012; **36**(1): 27–34. doi: 10.1080/03115518.2011.576535

312. Gu J-J, Yue Y, Shi F *et al.* First Jurassic grasshopper (Insecta, Caelifera) from China. *Zootaxa*. 2016; **4169**(2): 377–380. doi: 10.11646/zootaxa.4169.2.9

313. Fang Y, Zhang H, Wang B. A new species of Aboilus (Insecta, Orthoptera, Prophalangopsidae) from the Middle Jurassic of Daohugou, Inner Mongolia, China. *Zootaxa*. 2009; **2249**(1): 63–68. doi: 10.11646/zootaxa.2249.1.6

314. Li L, Ren D, Wang ZH. New prophalangopsids from late Mesozoic of China (Orthoptera, Prophalangopsidae, Aboilinae). *Acta Zootaxonomica Sinica*. 2007; **32**: 412–422.

315. Wang H, Li S, Zhang Q *et al.* A new species of Aboilus (Insecta, Orthoptera) from the Jurassic Daohugou beds of China, and discussion of forewing coloration in Aboilus. *Alcheringa: An Australasian Journal of Palaeontology*. 2015; **39**(2): 250–258. doi: 10.1080/03115518.2015.993297

316. Ren D, Meng X. New Jurassic protaboilins from China (Orthoptera: Prophalangopsidae, Protaboilinae). *Acta Zootaxonomica Sinica*. 2006; **31**(3): 513–519. doi: 10.3969/j.issn.1000-0739.2006.03.010

317. Wang H, Fang Y, Wang B *et al.* The Jurassic orthopteran Allaboilus gigantus Ren and Meng, 2006 (Prophalangopsidae) from Beipiao, Northeast China and its biostratigraphical significance. *Proceedings of the Geologists' Association*. 2018; **129**(5): 629–634. doi: 10.1016/j.pgeola.2018.04.006

318. Gu J-J, Qiao G-X, Ren D. Revision and New Taxa of Fossil Prophalangopsidae (Orthoptera: Ensifera). *Journal of Orthoptera Research*. 2010; **19**(1): 41–56. doi: 10.1665/034.019.0110

319. Li L, Ren D, Meng X. New fossil prophalangopsids from China (Orthoptera, Prophalangopsidae, Aboilinae). *Acta Zootaxonomica Sinica*. 2007; **32**: 174–181. doi: 10.3969/j.issn.1000-0739.2007.01.030

320. Lin Q-b, Huang D-y, Nel A. A new genus of Chifengiinae (Orthoptera: Ensifera: Prophalangopsidae) from the Middle Jurassic (Jiulongshan Formation) of Inner Mongolia, China. *Comptes Rendus Palevol*. 2008; **7**(4): 205–209. doi: 10.1016/j.crpv.2008.02.003

321. Gu J-J, Qiao G-X, Ren D. A exceptionally-preserved new species of Barchaboilus (Orthoptera: Prophalangopsidae) from the Middle Jurassic of Daohugou, China. *Zootaxa*. 2011; **2909**(1): 64–68. doi: 10.11646/zootaxa.2909.1.7

322. Wang H, Fang Y, Zhang Q *et al.* New material of Sigmaboilus (Insecta, Orthoptera, Prophalangopsidae) from the Jurassic Daohugou Beds, Inner Mongolia, China. *Earth and Environmental Science Transactions of the Royal Society of Edinburgh*. 2018; **107**(2-3): 177–183. doi: 10.1017/s1755691017000172

323. Gu J, Zhao Y, Ren D. New fossil Prophalangopsidae (Orthoptera, Hagloidea) from the Middle Jurassic of Inner Mongolia, China. *Zootaxa*. 2009; **2004**: 16–24. doi: 10.5281/zenodo.185632

324. Fang YAN, Zhang H, Wang BO *et al.* New taxa of Aboilinae (Insecta, Orthoptera, Prophalangopsidae) from the Middle Jurassic of Daohugou, Inner Mongolia, China. *Zootaxa*. 2007; **1637**(1): 55–62. doi: 10.11646/zootaxa.1637.1.5

325. Huang D-Y, Nel A, Azar D *et al.* Phylogenetic relationships of the Mesozoic paraneopteran family Archipsyllidae (Insecta: Psocodea). *Geobios*. 2008; **41**(4): 461–464. doi: 10.1016/j.geobios.2007.11.003

326. Shang L, BÉThoux O, Ren D. New stem-Phasmatodea from the Middle Jurassic of China. *European Journal of Entomology*. 2011; **108**(4): 677–685. doi: 10.14411/eje.2011.086

327. Liu Y, Ren D, Nina D ND *et al.* A new Middle Jurassic stonefly from Daohugou, Inner Mongolia, China (Insecta: Plecoptera). *Annales Zoologici*. 2006; **56**(3): 549–554. doi: 10.3161/000345406778811916

328. Liu Ys, Sinitshenkova ND, Ren D. A revision of the Jurassic Stonefly Genera Dobbertiniopteryx Ansorge and Karanemoura Sinitshenkova (Insecta: Plecoptera), with the description of new species from the Daohugou locality, China. *Paleontological Journal*. 2009; **43**(2): 183–190. doi: 10.1134/s0031030109020099

329. Cui Y, Ren D, Béthoux O. The Pangean journey of ‘south forestflies’ (Insecta: Plecoptera) revealed by their first fossils. *Journal of Systematic Palaeontology*. 2018; **17**(3): 255–268. doi: 10.1080/14772019.2017.1407370

330. Yushuang L, Sinitshenkova ND, Dong R *et al.* Pronemouridae fam. nov. (Insecta: Plecoptera), the stem group of nemouridae and notonemouridae, from the Middle Jurassic of Inner Mongolia, China. *Palaeontology*. 2011; **54**(4): 923–933. doi: 10.1111/j.1475-4983.2011.01063.x

331. Cui Y, Béthoux O, Kondratieff B *et al.* The first fossil salmonfly (Insecta: Plecoptera: Pteronarcyidae), back to the Middle Jurassic. *BMC Evolutionary Biology*. 2016; **16**(1): 1–14. doi: 10.1186/s12862-016-0787-9

332. Liu Y, Ren D, Sinitshenkova ND *et al.* The oldest known record of Taeniopterygidae in the Middle Jurassic of Daohugou, Inner Mongolia, China (Insecta: Plecoptera). *Zootaxa*. 2007; **1521**: 1–8. doi: 10.5281/zenodo.177457

333. Liu Y, Ren D. Two new Jurassic stoneflies (Insecta: Plecoptera) from Daohugou, Inner Mongolia, China. *Progress in Natural Science*. 2008; **18**(8): 1039–1042. doi: 10.1016/j.pnsc.2008.03.014

334. Liu X, Ren D, Yang D. New transitional fossil snakeflies from China illuminate the early evolution of Raphidioptera. *BMC Evolutionary Biology*. 2014; **14**(1): 1–15. doi: 10.1186/1471-2148-14-84

335. LÜ Y-N, Liu X, Dong REN. First record of the fossil snakefly genus Mesoraphidia (Insecta: Raphidioptera: Mesoraphidiidae) from the Middle Jurassic of China, with description of a new species. *Zootaxa*. 2015; **3999**(4): 560–570. doi: 10.11646/zootaxa.3999.4.6

336. Lyu Y-N, Ren D, Liu X. Review of the fossil snakefly family Mesoraphidiidae (Insecta: Raphidioptera) in the Middle Jurassic of China, with description of a new species. *Alcheringa: An Australasian Journal of Palaeontology*. 2017; **41**(3): 403–412. doi: 10.1080/03115518.2017.1298840

337. Engel MS, Ren D. New Snakeflies from the Jiulongshan Formation of Inner Mongolia, China (Raphidioptera). *Journal of the Kansas Entomological Society*. 2008; **81**(3): 188–193. doi: 10.2317/jkes-802.19.1

338. Huang D, Engel MS, Cai C *et al.* Mesozoic giant fleas from northeastern China (Siphonaptera): Taxonomy and implications for palaeodiversity. *Chinese Science Bulletin*. 2013; **58**(14): 1682–1690. doi: 10.1007/s11434-013-5769-3

339. Gao T-p, Shih C-k, Xu X *et al.* Mid-Mesozoic Flea-like Ectoparasites of Feathered or Haired Vertebrates. *Current Biology*. 2012; **22**(8): 732–735. doi: 10.1016/j.cub.2012.03.012

340. Nel P, Retana-Salazar AP, Azar D *et al.* Redefining the Thripida (Insecta: Paraneoptera). *Journal of Systematic Palaeontology*. 2014; **12**(7): 865–878. doi: 10.1080/14772019.2013.841781

341. Wang M, Zhao Y, Ren D. New fossil caddisfly from Middle Jurassic of Daohugou, Inner Mongolia, China (Trichoptera: Philopotamidae). *Progress in Natural Science*. 2009; **19**(10): 1427–1431. doi: 10.1016/j.pnsc.2009.01.012

342. Gao Y, Yao Y, Ren D. A new Middle Jurassic caddisfly (Trichoptera, Hydrobiosidae) from China. *Fossil Record*. 2013; **16**(1): 111–116. doi: 10.1002/mmng.201300005

343. Zhang W, Shih C, Ren D. Two new fossil caddisflies (Amphiesmenoptera: Trichoptera) from the Middle Jurassic of northeastern China. *Alcheringa: An Australasian Journal of Palaeontology*. 2016; **41**(1): 22–29. doi: 10.1080/03115518.2016.1170501

344. Liu Z-J, Liu Y, Zhang W *et al.* A New Fossil of Necrotauliidae (Insecta: Trichoptera) from the Jiulongshan Formation of China and Its Taxonomic Significance. *PLoS ONE*. 2014; **9**(12). doi: 10.1371/journal.pone.0114968

345. Hao W, Diying H. A New Species of Liadotaulius (Insecta: Trichoptera) from the Middle Jurassic of Daohugou, Inner Mongolia. *Acta Geologica Sinica - English Edition*. 2012; **86**(2): 320–324. doi: 10.1111/j.1755-6724.2012.00662.x

346. Gao Y, Yao Y, Ren D. New Genus and Species of Rhyacophilidae (Insecta: Trichoptera) from the Middle Jurassic of China. *Acta Geologica Sinica - English Edition*. 2013; **87**(6): 1495–1500. doi: 10.1111/1755-6724.12153
