# Supplementary material for: Reconstructing vegetation biomass in the Middle Jurassic Yanliao Biota from insect fossil assemblages
Source: Natl Sci Rev. 2026 Jun 2;13(12):nwag329. doi: 10.1093/nsr/nwag329 (PMC13321125; doi:10.1093/nsr/nwag329)
Supplement: nwag329_Supplemental_Files [file nwag329_supplemental_files.zip › supplementary Information.docx]

Supplementary Materials for

Reconstructing vegetation biomass in the Middle Jurassic Yanliao Biota from insect fossil assemblages

Liang Chen^1,2^, Shilong Guo^1,2^, Lifang Xiao^2,3^, Nan Yang^1^, Chungkun Shih^1,2^, Conrad C. Labandeira^1,2,4^**^*^**, Chaofan Shi^5^**^*^**, Dong Ren^1^**^*,^**

1 College of Life Sciences, Capital Normal University; Beijing, 100048, China

2 Department of Paleobiology, National Museum of Natural History; Smithsonian Institution, Washington, DC 20013-7012, USA.

3 Institute of Zoology Guangdong Academy of Science; Guangdong, 510260, Chin

4 Department of Entomology, University of Maryland; College Park, Maryland, 20742, USA.

5 School of Ecology, Sun Yat-sen University; Guangzhou, 510275, China

### * Corresponding authors: Conrad C. Labandeira (email: [labandec@si.edu)](mailto:labandec@si.edu)), Chaofan Shi (email: [shichf5@mail.sysu.edu.cn)](mailto:shichf5@mail.sysu.edu.cn)), and Dong Ren (Lead contact, email: [rendong@mail.cnu.edu.cn)](mailto:rendong@mail.cnu.edu.cn))

**Materials**

**Fossil Insect Dataset**

Our research focuses on the Daohugou locality situated in the Ningcheng area of Inner Mongolia, in Northeastern China. This region has proven to be richly endowed with paleontological discoveries, especially numerous and diverse fossil taxa unearthed and carefully documented over the past four decades [1]. The Daohugou locality holds a prominent place in scientific exploration, providing a rich history of broad studies within China and is recognized internationally [2]. Leveraging the wealth of information derived from the species described at this locality, we have provided species data in this dataset is derived from published literature, encompassing 749 insect species belonging to 417 genera, 154 families, and 25 orders, highlighting the region's biodiversity. Among these, herbivorous insects consist of 318 species, belonging to 183 genera, 72 families, and 16 orders.

These fossilized specimens represent a diverse array of late Middle Jurassic (Callovian), 165-million-year-old [3] insect taxa that highlight the intricate ecosystems present in the Daohugou area. To enhance the accessibility of this dataset, we extracted images of species from the relevant literature, creating a visual repository that aided in the identification and understanding of these ancient insect species. The assembled species-level information, detailed in the attached Insect Data, lays the groundwork for a thorough exploration of the paleontological landscape at the Daohugou locality.

Our fossil insect dataset not only contributes to our understanding of ancient biodiversity but also provides a valuable resource for comparative studies, allowing for insights into the evolutionary dynamics of insect communities over time. The updating of this dataset ensures its reliability and utility as a reference for research endeavors in future studies of fossil insects.

**Live Insect Collection**

To elucidate the patterns of estimated insect weights and to make meaningful comparisons with their fossil counterparts, a wide range of living insects were carefully collected using trap nets from various habitats in the Beijing region. These live insects provided actualistic data. The live insects were then stored in centrifuge tubes; underwent weight measurements and were photographed on the same day as their collection. The specimens were collected from the Baiyanggou Natural Scenic Area, located on Beihe Road in the Changping District of Beijing, with geographical coordinates ranging from 40.19°N to 40.22°N and 115.9°E to 116.1°E.

The collection effort yielded a wide variety of modern insect species, including specimens representing 77 taxonomic groups across multiple orders (Table S4). The collection of varied specimens reflecting numerous lineages of insects ensured that we provided a reference base and calibration for weight estimates of a variety of insect species. This allowed us to be more accurate in our fossil insect estimates.

**Methods**

**Overview**

We developed a probabilistic modelling framework to reconstruct vegetation biomass from fossil insect assemblages by integrating trait-based biomass estimation, ecological scaling relationships, and trophic transfer processes. Uncertainty arising from fossil measurements, ecological parameters, and model structure was explicitly stated using Monte Carlo simulation and bootstrap-based calibration.

All model components were implemented in R (version ≥ 4.2), and all stochastic processes were evaluated through repeated simulations to obtain empirical posterior distributions of key ecological variables.

**Estimation of insect body mass from fossil traits**

Due to the inherent limitations of fossil preservation, direct measurement of insect body mass is not possible. Therefore, insect body mass was estimated using established allometric relationships between body size and body mass. Because many fossil specimens lack complete bodies, body- and wing-related morphological traits were treated separately when available.

To establish and validate the allometric model, 77 species of extant insects representing nine orders were collected. For each specimen, individuals were photographed, measured, and weighed. Body mass was then estimated using the quantitative relationship proposed by Sample et al. [4], and estimated values were compared with directly measured masses.

Although a strong correspondence was observed between estimated and measured body masses, the estimated values were generally slightly lower than the measured ones. This bias may result from the relatively small sample size used for model validation.

Individual insect body mass was estimated from fossil morphological measurements using established relationships. For each fossil specimen, estimated body mass was calculated as:

 S1

In the above equation, *M* is the mass of the insect. Both *a* and *b* are the parameters of the different species, and *S* is the area of the insect from its fossil compression.

To account for uncertainty in the allometric relationship, parameters $a$and $b$were treated as stochastic variables rather than fixed values. For each Monte Carlo iteration, parameter values were sampled from normal distributions defined by their estimated means and standard errors:

$a\sim\mathcal{N(}\mu_{a},\sigma_{a}),b\sim\mathcal{N(}\mu_{b},\sigma_{b})$ S2

$\mu_{a},\mu_{b}$ are the means. $\sigma_{a},\sigma_{b}$ are the standard errors. In addition, calibration uncertainty was incorporated by randomly sampling correction coefficients from a bootstrap-derived distribution of calibration regressions. This approach allows both measurement error and model uncertainty to be propagated into the final insect body mass estimates.

We collected images of the species at the target site and used Adobe Photoshop software to process the images to obtain information on pixel positions. The body size of the species can be calculated by combining the image scale size for scale substitution.

 S3

*S* is the area of the insect species outlined on the fossil slab; *P* is the number of pixels of the insect species on the outlined fossil slab in the image; *P_m_* is the number of pixels of the scaled bar next to the image; *X* is the size of the scale bar in the image.

**Bootstrap calibration of fossil to modern conversion**

To correct for systematic bias between fossil-derived estimates and modern reference data, a bootstrap calibration procedure was applied. For each bootstrap iteration, the calibrated dataset was resampled with replacement, and a linear correction model was fitted:

$W_{\text{calibrated}}=\alpha W_{\text{estimated}}+\beta$ S4

The resulting distributions of calibration parameters $\alpha$and $\beta$were used as empirical priors during Monte Carlo simulations, with one parameter pair randomly sampled per iteration. This procedure propagates calibration uncertainty into all downstream biomass estimates.

**Estimation of insect population density**

Insect population density was estimated using a body mass–abundance scaling relationship, expressed in log–log form:

$\log_{10}(D)=\alpha+ \beta\cdot\log_{10}(W)$ S5

Where *D* is population density, $W$ is individual body mass, $\beta$ is the theoretical scaling exponent, and $\alpha$ is the intercept

The scaling exponent $s$ was fixed at a theoretically derived value (−0.75), consistent with metabolic scaling theory [5–7], while the intercept was estimated from empirical data. Density estimates were rounded to integer values to reflect discrete individual counts.

**Input Data Analysis**

Insect density (ID) [8] refers to the number of insect individuals per unit area. Based on previous studies, insect density is approximately 1788 individuals per square meter [9]; in streams, insect density is 225 (±280) individuals per square meter [10]; and in shallow wetlands, insect density is approximately 205 individuals per square meter [11]. Through comprehensive data analysis, we conclude that the lower limit of modern insect community density is 205 ind/m², and the upper limit is 1788 ind/m². This result is compared with the insect density at Daohugou assessed by the Monte Carlo model.

**Reconstruction of insect biomass**

Total insect biomass (IB) was calculated as the product of individual body mass and estimated population density for each Monte Carlo iteration:

$\text{IB}=W\times D$ S6

Multiple insect assemblages were analysed by resampling fossil datasets at different quantile levels, allowing assessment of sensitivity to sampling structure and preservation bias.

**Trophic transfer model for vegetation biomass**

Plants provide energy for phytophagous animals. The feeding rate (FR) [12], reflects the amount of plant biomass consumed (CB) by phytophagous insects. As phytophagous insects, the energy they consume can be represented by the reduction of plant biomass [12]. It was found that the consumption by herbivores accounted for 5.1–13% of leaf production [13, 14]. In the comprehensive analysis, we set 5.1% as the lower limit and 13% as the upper limit. A Monte Carlo evaluation was used to simulate uncertainty to obtain the net primary productivity (NPP) of plants.

The data of the plant biomass accumulation rate (AR) indicates the relative transformation ability of the plant [15]. The habitat environment in the Middle Jurassic Daohugou area is suggested to be aquatic, semiaquatic, or nearshore wetland inhabited by mayflies, stoneflies, aquatic beetles, lacewings, and dragonflies, among others, such that the environment would contain aquatic plants, wetland equivalent of grasses, shrubs, and other plant types. Based on the published literature, the accumulation rate (AR) of herbaceous plants is about 1.4 times, and that of woody plants is about 3.9 times [15]. The range of AR for forests and shrubs in Brookhaven, New York, USA, is 1.6~8.7 times. The AR of the North American wormwood shrubs along the Southern California coast is 4.0 times. The AR of a forest community can reach 4~10.3 times [15]. In a comprehensive analysis, we use 1.4 times as the lower limit and 10.3 times as the upper limit and performed a simulation to assess the uncertainty and obtain the biomass of the plants in the community.

Vegetation biomass (VB) was reconstructed using a trophic transfer model that links plant biomass to herbivorous insect biomass via three key ecological parameters: Feeding rate (FR) [12–14], Accumulation rate (AR) [15–17], Transfer rate (TR) [18, 19].

Vegetation biomass was calculated as:

$\text{VB}=\frac{\text{IB}}{\text{TR}\times\text{FR}\times\text{AR}}$ S7

Each parameter was treated as uncertain and sampled from log-normal distributions defined by biologically plausible lower and upper bounds. Sampling was performed in log space to ensure positivity and to reflect multiplicative ecological processes.

**Monte Carlo uncertainty propagation**

Uncertainty propagation was conducted using Monte Carlo simulation with $n=10,000$ iterations per model run. In each iteration, all stochastic components—including body mass estimation, calibration parameters, population density, and trophic transfer rates—were independently sampled.

The resulting posterior distributions of IB and VB were summarized using empirical means and 95% uncertainty intervals (2.5–97.5% quantiles). This approach avoids parametric assumptions about output distributions and allows direct quantification of propagated uncertainty.

**Sensitivity and uncertainty attribution analysis**

To quantify the relative contribution of individual parameters to uncertainty in vegetation biomass, standardized regression coefficient (SRC) analysis was applied. All model inputs and outputs were log-transformed and standardized prior to regression:

$\text{scale}(\text{VB})\sim\text{scale}(\text{IB})+\text{scale}(\text{FR})+\text{scale}(\text{AR})+\text{scale}(\text{TR})$ S8

The absolute magnitude of SRC values was used as a measure of parameter sensitivity, while confidence intervals were derived from regression uncertainty.

In addition, scenario-based parameter range combinations were evaluated to assess the robustness of VB estimates under alternative ecological assumptions.

**Reproducibility**

All analyses were conducted using open-source R packages, including *dplyr*, *ggplot2*, *raster*, and *rasterVis*. All scripts and data processing steps were designed to ensure full reproducibility and transparency.

**Table S1. Taxa, body density, and body weight of modern arthropod species similar to the Daohugou insect community.**

| **Taxon** | **Density**  **ind/m^2^** | **Weight**  **/mg** | **Source** | **Taxon** | **Density**  **ind/m^2^** | **Weight**  **/mg** | **Source** |
| --- | --- | --- | --- | --- | --- | --- | --- |
| ***Terrestrial arthropods***  *Anomma nigricans* | 34.9 | 9.17 | [20] | *Cladotanytarsus mancus* | 940 | 0.128 | [21] |
| *Armadillidium vulgare* | 430 | 24 | [22] | *Corixa germari* | 1910 | 22.2 | [23] |
| *Bootettix punctatus* | 0.043 | 86 | [24] | *Crangonyx richmondensis* | 283 | 0.715 | [25] |
| *Camponotus acvapimensis* | 220 | 5.1 | [20] | *Cryptochironomus supplicans* | 428 | 0.301 | [26] |
| *Carabodes minusculus* | 9700 | 0.028 | [27] | *Cypria opthalmica* | 17100 | 0.0056 | [28] |
| *Chomohates schiitzi* | 4200 | 0.0083 | [27] | *Daphnia cucullata* | 11900 | 0.0065 | [29] |
| *Leptopterna dolabrata* | 2.29 | 18.3 | [30] | *Daphnia hyalina* | 22700 | 0.0065 | [29] |
| *Ligidium hypnorum* | 130 | 6.9 | [31] | *Ephemerella subvarica* | 3060 | 2.1 | [32] |
| *Ligidium japonicum* | 55 | 8.7 | [22] | *Erpobdella octoculata* | 117 | 4.23 | [26, 33] |
| *Nanhermannia nana* | 1590 | 0.017 | [27] | *Eurycercus lamellatus* | 105 | 0.0119 | [34, 35] |
| *Narceus americanus* | 0.29 | 2500 | [36] | *Ferrissia rivularts* | 703 | 2.29 | [37] |
| *Neophilaenus lineatus* | 29.4 | 2.54 | [38] | *Gammarus tigrinus* | 3400 | 4.24 | [39] |
| *Olodiscus minima* | 2100 | 0.016 | [27] | *Glyptotendipes glaucus* | 1870 | 0.112 | [28] |
| *Orchelimum fidicinium* | 22.1 | 156 | [40] | *Glyptotendipes paripes* | 2170 | 2.1 | [21, 26] |
| *Philoscia muscorum* | 212 | 2.41 | [41, 42] | *Gyraulus deflectus* | 5370 | 1.14 | [43] |
| *Platynothrus peltifer* | 1300 | 0.056 | [27] | *Gyraulus parvus* | 60 | 0.864 | [44] |
| *Pogonomyrmex badius* | 11.6 | 6.67 | [45] | *Hedriodiscus truquii* | 14 | 75 | [26, 46] |
| *Porcellio scaber* | 70 | 9 | [22] | *Helobdella stagnalis* | 233 | 1.86 | [34] |
| *Tetramorium caespitum* | 4870 | 0.60 | [47] | *Heterotrissocladius oliveri* | 1640 | 0.27 | [36, 48] |
| *Trichoniscus pusillus* | 811 | 0.66 | [42, 49] | *Hexagenia limbata* | 47.4 | 11 | [25, 50] |
| *Tracheoniscus rathkei* | 16.1 | 21 | [36] | *Hyalella azteca* | 1910 | 4.18 | [51, 52] |
| *Trimerotopsis saxatalis* | 0.20 | 144 | [53] | *Hydra oligactis* | 413 | 0.0629 | [26] |
| ***Terrestrial invertebrates***  *Acanthinula aculeata* | 71.5 | 9.43 | [54] | *Hydrozetes lacustris* | 41000 | 0.0045 | [28] |
| *Agriolimax laeuis* | 0.38 | 73.7 | [55] | *Ilyocryptus sordidus* | 3220 | 0.0089 | [28] |
| *Agriolimax reticulatus* | 1.29 | 2070 | [55] | *Ilyodrilus hammoniensis* | 5500 | 9.78 | [34] |
| *Allolobophora caliginosa* | 53.9 | 218 | [56] | *Isoplastis monilis* | 333 | 0.0281 | [28] |
| *Allolobophora chlorotica* | 2.97 | 153 | [56] | *Lacuna vincta* | 10 | 3 | [57] |
| *Allolobophora longa* | 3.58 | 1180 | [56] | *Limnephilus lunatus* | 44 | 1.19 | [28] |
| *Allolobophora muldali* | 12.4 | 15.5 | [56] | *Limnocalanus macrurus* | 25500 | 0.0118 | [58] |
| *Allolobophora rosea* | 34.4 | 116 | [56] | *Limnochironomus pulsus* | 955 | 0.556 | [21, 26] |
| *Arianta arbustorum* | 4.4 | 554 | [54] | *Littorina saxatalis* | 236 | 1.58 | [57] |
| *Arion ater* | 1.21 | 1250 | [55] | *Lumbriculus variegatus* | 658 | 1.81 | [26] |
| *Arion fasciatus* | 6.13 | 158 | [55] | *Lymnaea palustris* | 800 | 36.6 | [44, 59] |
| *Arion hortensis* | 5.88 | 155 | [55] | *Melampus linealus* | 129 | 6 | [57] |
| *Arion intermedim* | 19 | 63.8 | [55] | *Microtendipes chloris* | 4950 | 0.128 | [60] |
| *Arion subjiuscus* | 0.79 | 272 | [55] | *Microtendipes* sp*.* | 895 | 1.71 | [21] |
| *Carychium tridentatum* | 120 | 0.2 | [54] | *Modiolus demissus* | 7.8 | 490 | [61] |
| *Cepaea nemoralis* | 4.03 | 150 | [62] | *Monodacna pontica* | 0.786 | 1490 | [63] |
| *Claudia bidentata* | 0.45 | 9.47 | [54] | *Mytilus edulus* | 471 | 20 | [57] |
| *Cochlicopa lubrica* | 0.9 | 3.83 | [54] | *Nassarius obsoletus* | 35 | 25 | [57] |
| *Columella edentula* | 3.37 | 0.84 | [54] | *Neanthes virens* | 5.19 | 2750 | [64] |
| *Dendrobaena mammalis* | 25.4 | 28.2 | [56] | *Oligophleboides sigma* | 4500 | 1.54 | [65] |
| *Dendrobaena rubida* | 2.31 | 52.9 | [56] | *Orconectes uirilis* | 1.02 | 9000 | [66] |
| *Discus rotundatus* | 13.5 | 5.19 | [54] | *Olomesostoma auditivum* | 240 | 0.133 | [26] |
| *Ena obscura* | 3.82 | 18.4 | [54] | *Pacifastacus lenuisculus* | 0.925 | 20000 | [67] |
| *Euconulus jiuluus* | 1.57 | 1.66 | [54] | *Parachironomus tener* | 778 | 0.156 | [21] |
| *Hygromia hispida* | 0.67 | 1.12 | [54] | *Pentaneura monilis* | 163 | 0.0184 | [26] |
| *Hygromia striolata* | 8.54 | 28.1 | [54] | *Ptysa grina* | 778 | 5.4 | [43] |
| *Lehmannia marginata* | 0.25 | 544 | [55] | *Physa integra* | 311 | 5.7 | [44] |
| *Lumbricus castaneus* | 13.6 | 58.5 | [56] | *Pisaster ochraceur* | 0.2 | 629000 | [68] |
| *Lumbricus terreslris* | 9.09 | 1860 | [56] | *Pisidium casertanum* | 5200 | 0.633 | [28, 34] |
| *Marpessa laminata* | 1.8 | 4.5 | [54] | *Pisidium compressum* | 4000 | 1.02 | [43] |
| *Millsonia anomala* | 1.8E–06 | 944 | [69] | *Potamophylax cingulatus* | 33.8 | 48.6 | [70] |
| *Octolasion cyaneum* | 75.1 | 606 | [56] | *Potamothrix hammoniensis* | 29000 | 3.48 | [71] |
| *Punctum pygmaeum* | 67.2 | 0.186 | [54] | *Pristina idrensis* | 50500 | 0.059 | [28] |
| *Pupilla muscorum* | 0.67 | 0.314 | [54] | *Procladius choreus* | 1830 | 0.439 | [21, 26] |
| *Retinella nitidula* | 7.86 | 4.88 | [54] | *Procladius pectinatus* | 300 | 2.33 | [34] |
| *Retinella pura* | 24.7 | 1.23 | [54] | *Procladius sagittalis* | 889 | 0.0776 | [28] |
| *Retinella radiatula* | 0.45 | 2.4 | [54] | *Pseudodiamensa arctica* | 122 | 2.7 | [48] |
| *Vallonia pulchella* | 0.67 | 1.12 | [54] | *Psilotanypus rufovittatus* | 6700 | 0.556 | [21] |
| *Vitrea contracta* | 39.1 | 0.72 | [54] | *Rhitrogena semicolorata* | 232 | 8.19 | [36, 72] |
| *Vitrea pellucida* | 1.57 | 2.75 | [54] | *Scobicularia plana* | 114 | 0.083 | [73, 74] |
| ***Aquatic invertebrates***  *Alona quadrangularis* | 9270 | 0.0048 | [28] | *Sialis lutaria* | 153 | 5.6 | [28, 75] |
| *Ampelisca brevicornis* | 91.5 | 12.6 | [76] | *Skistodiaptomus oregonensis* | 157000 | 0.015 | [77] |
| *Anatopynia goetghebueri* | 8930 | 0.048 | [28] | *Stronglocentrotus droebachensis* | 36.8 | 2640 | [78] |
| *Anodonta anatina* | 11.7 | 5530 | [79] | *Tanytarsus eminulus* | 4600 | 0.015 | [28] |
| *Anodonta minima* | 0.388 | 5390 | [79] | *Tanytarsus holochlorus* | 4310 | 0.334 | [21] |
| *Aodonta piscinalis* | 1.49 | 6470 | [63] | *Tanytarsus inopterus* | 3590 | 0.284 | [21] |
| *Asellus aquaticus* | 21.9 | 0.94 | [26, 34] | *Tanytarsus jucundus* | 7190 | 2.05 | [52] |
| *Arthripsodes ancylus* | 6 | 0.5 | [80] | *Tanytarsus lugens* | 4510 | 0.426 | [21] |
| *Baetis rhodani* | 290 | 2.16 | [72] | *Tegula funebralis* | 600 | 702 | [36] |
| *Baetis vagans* | 2680 | 0.49 | [81] | *Thermocyclops hyalinus* | 754000 | 0.0022 | [82] |
| *Bithynia tentaculata* | 18.4 | 25.5 | [34, 83] | *Tripha* sp. | 1.56 | 0.0031 | [28] |
| *Chaospecta dives* | 26600 | 1.98 | [36] | *Unio pictorum* | 5.26 | 5840 | [63, 79] |
| *Chaoborus flavicans* | 1750 | 4.46 | [34] | *Unio tumidus* | 2.5 | 6970 | [63, 79] |
| *Chironomus anthracinus* | 8950 | 7.22 | [34] | *Valvata humeralis* | 2090 | 1.92 | [43] |
| *Chironomus longistylus* | 244 | 0.166 | [28] | *Vejdovskyella comaia* | 41900 | 0.0082 | [28] |
| *Chironomus plumosus* | 107 | 4.55 | [21, 26] |  |  |  |  |

1. From ref [6].

**Table S2.** **Parameters for estimating the body weight of various species.**

| **Taxon** | **a** ± SE | **b** ± SE | **r** |
| --- | --- | --- | --- |
| Insecta | 1.331±0.011 | –2.140±0.036 | 0.94 |
| Plecoptera | 1.206±0.071 | –2.024±0.036 | 0.94 |
| Hemiptera | 1.423±0.041 | –2.765±0.135 | 0.97 |
| Neuroptera | 1.172±0.033 | –1.684±0.146 | 0.98 |
| Mecoptera | 1.219±0.254 | –1.801±0.758 | 0.76 |
| Trichoptera | 1.501±0.055 | –2.369±0.132 | 0.95 |
| Coleoptera | 1.296±0.030 | –1.857±0.095 | 0.92 |
| Diptera | 1.309±0.030 | –2.197±0.089 | 0.94 |

1. From ref [4]

2. r = Pearson correlation coefficient.

**Table S3. Body weight estimates and measurements of live insects**

| Taxon | Scale starting pixel | Scale ending pixel | Actual scale size/mm | Body pixels | Body area/mm^2^ | Weight of estimated/mg | Weight of measured/mg |
| --- | --- | --- | --- | --- | --- | --- | --- |
| Coleoptera | 484 | 541 | 1 | 218644 | 67.3 | 73.05 | 68 |
|  | 404 | 469 | 1 | 194689 | 46.08 | 44.72 | 54 |
|  | 259 | 292 | 1 | 80390 | 73.82 | 82.36 | 127 |
|  | 495 | 591 | 1 | 187368 | 20.33 | 15.49 | 28 |
|  | 489 | 544 | 1 | 132269 | 43.73 | 41.78 | 52 |
|  | 307 | 370 | 1 | 132206 | 33.31 | 29.36 | 42 |
|  | 436 | 526 | 1 | 164820 | 20.35 | 15.5 | 21 |
|  | 527 | 579 | 1 | 152292 | 56.32 | 58 | 111 |
|  | 505 | 553 | 1 | 47072 | 20.43 | 15.58 | 22 |
|  | 191 | 217 | 1 | 53975 | 79.84 | 91.17 | 171 |
|  | 509 | 567 | 1 | 281574 | 83.7 | 96.92 | 169 |
|  | 291 | 323 | 1 | 91535 | 89.39 | 105.54 | 205 |
|  | 370 | 524 | 1 | 240597 | 10.14 | 6.29 | 11 |
|  | 395 | 497 | 1 | 181405 | 17.44 | 12.69 | 19 |
|  | 248 | 283 | 1 | 92620 | 75.61 | 84.95 | 205 |
|  | 379 | 430 | 1 | 58509 | 22.49 | 17.65 | 26 |
|  | 480 | 537 | 1 | 90619 | 27.89 | 23.33 | 33 |
|  | 549 | 597 | 1 | 216431 | 93.94 | 112.55 | 327 |
|  | 432 | 483 | 1 | 238462 | 91.68 | 109.06 | 313 |
|  | 323 | 415 | 1 | 134534 | 15.89 | 11.26 | 23 |
|  | 467 | 594 | 1 | 224935 | 13.95 | 9.5 | 15 |
|  | 479 | 585 | 1 | 132253 | 11.77 | 7.63 | 12 |
|  | 431 | 500 | 1 | 119378 | 25.07 | 20.32 | 30 |
|  | 277 | 352 | 1 | 115995 | 20.62 | 15.77 | 26 |
|  | 389 | 515 | 1 | 249075 | 15.69 | 11.07 | 13 |
|  | 478 | 550 | 1 | 117034 | 22.58 | 17.74 | 37 |
| Diptera | 186 | 238 | 1 | 36534 | 13.51 | 6.71 | 10 |
|  | 262 | 364 | 1 | 167666 | 16.12 | 8.46 | 14 |
|  | 436 | 488 | 1 | 151892 | 56.17 | 43.35 | 50 |
|  | 62 | 96 | 1 | 29542 | 25.56 | 15.46 | 25 |
|  | 355 | 407 | 1 | 105890 | 39.16 | 27.03 | 46 |
|  | 309 | 360 | 1 | 40166 | 15.44 | 8 | 9 |
|  | 415 | 467 | 1 | 60652 | 22.43 | 13.04 | 23 |
| Hemiptera | 128 | 179 | 1 | 75095 | 28.87 | 15.08 | 36 |
|  | 143 | 201 | 1 | 94864 | 28.2 | 14.58 | 15 |
|  | 323 | 404 | 1 | 74535 | 11.36 | 4 | 12 |
|  | 361 | 444 | 1 | 58426 | 8.48 | 2.64 | 8 |
|  | 440 | 513 | 1 | 73460 | 13.78 | 5.27 | 9 |
|  | 336 | 407 | 1 | 65257 | 12.95 | 4.82 | 14 |
|  | 341 | 423 | 1 | 185918 | 27.65 | 14.18 | 19 |
|  | 590 | 688 | 1 | 147682 | 15.38 | 6.15 | 13 |
|  | 489 | 557 | 1 | 84256 | 18.22 | 7.83 | 10 |
|  | 478 | 499 | 1 | 20321 | 46.08 | 29.33 | 57 |
|  | 464 | 485 | 1 | 18230 | 41.34 | 25.13 | 38 |
|  | 450 | 470 | 1 | 18547 | 46.37 | 29.6 | 55 |
|  | 356 | 448 | 1 | 163762 | 19.35 | 8.53 | 8 |
|  | 489 | 511 | 1 | 14606 | 30.18 | 16.06 | 51 |
| Hymenoptera | 94 | 125 | 1 | 40477 | 42.12 | 43.14 | 50 |
|  | 143 | 191 | 1 | 110411 | 47.92 | 52.05 | 60 |
|  | 36 | 57 | 1 | 92581 | 209.93 | 447.23 | 627 |
|  | 132 | 183 | 1 | 134551 | 51.73 | 58.18 | 94 |
|  | 339 | 439 | 1 | 64037 | 6.4 | 2.78 | 4 |
|  | 431 | 482 | 1 | 45457 | 17.48 | 11.98 | 17 |
|  | 255 | 300 | 1 | 79546 | 39.28 | 38.97 | 43 |
|  | 251 | 302 | 1 | 76366 | 29.36 | 25.51 | 44 |
|  | 119 | 188 | 1 | 106734 | 22.42 | 17.22 | 31 |
|  | 249 | 304 | 1 | 36920 | 12.2 | 7.11 | 8 |
|  | 165 | 213 | 1 | 52332 | 22.71 | 17.55 | 29 |
| Homoptera | 354 | 392 | 1 | 122319 | 84.71 | 86.17 | 72 |
|  | 110 | 162 | 1 | 68397 | 25.29 | 17.6 | 30 |
|  | 203 | 323 | 1 | 38861 | 2.7 | 0.93 | 1.6 |
|  | 390 | 505 | 1 | 106411 | 8.05 | 3.91 | 6 |
|  | 330 | 447 | 1 | 111513 | 8.15 | 3.97 | 7 |
|  | 312 | 363 | 1 | 172623 | 66.37 | 62.53 | 66 |
| Lepidoptera | 435 | 459 | 1 | 15427 | 26.78 | 18.71 | 37 |
|  | 93 | 117 | 1 | 32396 | 56.24 | 50.23 | 77 |
|  | 169 | 203 | 1 | 67982 | 58.81 | 53.3 | 92 |
|  | 415 | 456 | 1 | 22084 | 13.14 | 7.25 | 9 |
| Neuroptera | 450 | 495 | 1 | 34310 | 16.94 | 10.23 | 18 |
|  | 400 | 445 | 1 | 30208 | 14.92 | 8.82 | 14 |
| Odonata | 417 | 447 | 1 | 50148 | 55.72 | 49.61 | 39 |
|  | 274 | 295 | 1 | 77824 | 176.47 | 230.13 | 230 |
|  | 278 | 299 | 1 | 71250 | 161.56 | 204.62 | 308 |
| Orthoptera | 415 | 442 | 1 | 68445 | 93.89 | 99.36 | 111 |
|  | 368 | 409 | 1 | 51194 | 30.45 | 22.2 | 39 |
|  | 422 | 463 | 1 | 68657 | 40.84 | 32.81 | 44 |
|  | 554 | 584 | 1 | 21122 | 23.47 | 15.7 | 35 |


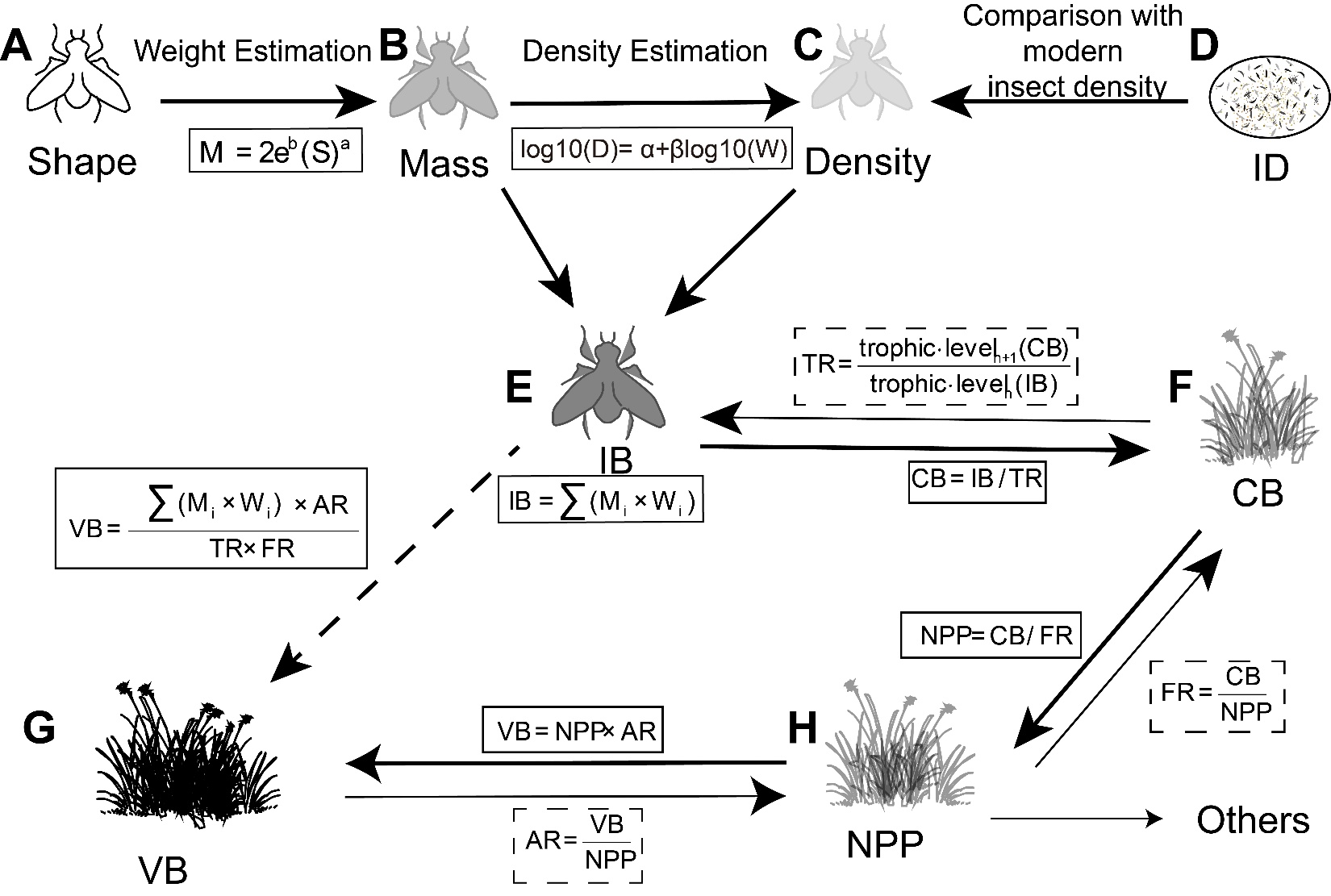


**Figure S1. The hypothetical flow chart for estimating vegetation biomass (VB).** **A**: The shape data for insects in the community. **B**: The body mass data of a population of insects. **C**: The estimated insect density in the community. **D**: The modern insect density (ID) distribution range of insects in the community. **E**: Insect biomass (IB) of phytophagous insects in the community. **F**: The consumed biomass (CB) by phytophagous insects. **G**: The vegetation biomass (VB) estimated in the community. **H**: The net primary production (NPP) in a community. The solid bold line indicates the calculation process. The solid thin line indicates the true direction of the biomass. The dashed lines indicate an indirect calculation process. The boxes outlined in solid lines indicates the formulas used for the calculations. The boxes outlined in dashed lines indicate the formulas needed for the calculation.


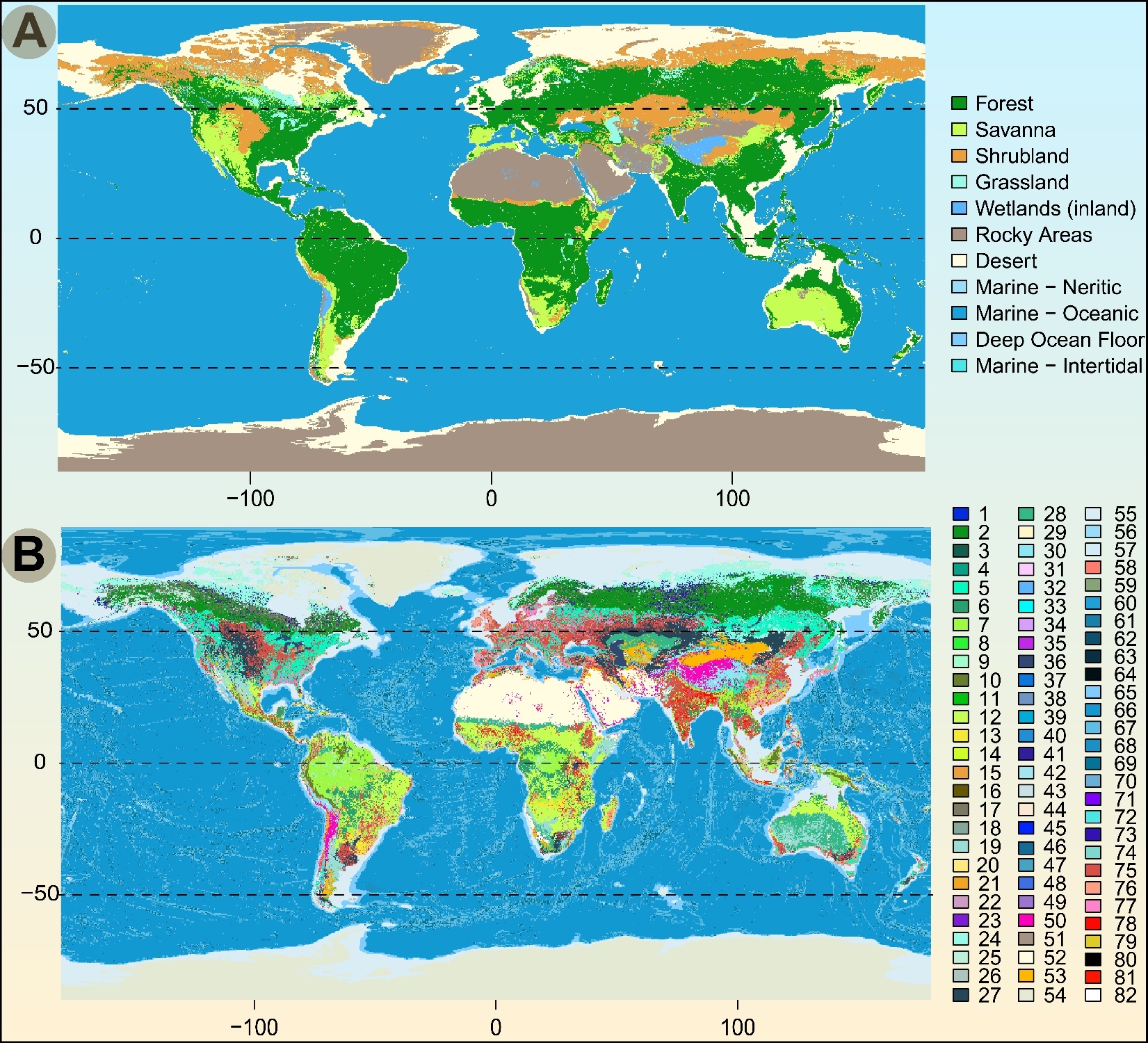


**Figure S2.** **Distribution of Modern Habitat Types on a Global Scale.** The global distribution of habitat types follows a structured framework established by the International Union for Conservation of Nature (IUCN), as outlined by the classification system presented by Martin Jung et al. **A**: visually represents the categorization of habitat classes at the level 1. **B**: visually represents the categorization of habitat classes at the level 2 as follows: 1: "Water"; 2: "Forest"; 3: "Forest–Boreal"; 4: "Forest–Subarctic"; 5: "Forest–Subantarctic"; 6: "Forest–Temperate"; 7: "Forest–Subtropical–tropical dry"; 8: "Forest–Subtropical–tropical moist lowland"; 9: "Forest–Subtropical–tropical mangrove vegetation"; 10: "Forest–Subtropical–tropical swamp"; 11: "Forest–Subtropical–tropical moist montane"; 12: "Savanna"; 13: "Savanna–Dry"; 14: "Savanna–Moist"; 15: "Shrubland"; 16: "Shrubland–Subarctic"; 17: "Shrubland–Subantarctic"; 18: "Shrubland–Boreal"; 19: "Shrubland–Temperate"; 20: "Shrubland–Subtropical–tropical dry"; 21: "Shrubland–Subtropical–tropical moist"; 22: "Shrubland–Subtropical–tropical high altitude"; 23: "Shrubland–Mediterranean–type"; 24: "Grassland"; 25: "Grassland–Tundra"; 26: "Grassland–Subarctic"; 27: "Grassland–Subantarctic"; 28: "Grassland–Temperate"; 29: "Grassland–Subtropical–tropical dry"; 30: "Grassland–Subtropical–tropical seasonally wet or flooded"; 31: "Grassland–Subtropical–tropical high altitude"; 32: "Wetlands (inland)"; 33: "Wetlands (inland)–Permanent rivers streams creeks"; 34: "Wetlands (inland)–Seasonal/intermittent/irregular rivers/streams/creeks"; 35: "Wetlands (inland)–Shrub dominated wetlands"; 36: "Wetlands (inland)–Bogs/marshes/swamps/fens/peatlands"; 37: "Wetlands (inland)–Permanent freshwater lakes"; 38: "Wetlands (inland)–Seasonal/intermittent freshwater lakes (over 8 ha)"; 39: "Wetlands (inland)–Permanent freshwater marshes/pools (under 8 ha)"; 40: "Wetlands (inland)–Seasonal/intermittent freshwater marshes/pools (under 8 ha)"; 41: "Wetlands (inland)–Freshwater springs and oases"; 42: "Wetlands (inland)–Tundra wetlands"; 43: "Wetlands (inland)–Alpine wetlands"; 44: "Wetlands (inland)–Geothermal wetlands"; 45: "Wetlands (inland)–Permanent inland deltas"; 46: "Wetlands (inland)–Permanent saline brackish or alkaline lakes"; 47: "Wetlands (inland)–Seasonal/intermittent saline brackish or alkaline lakes and flats"; 48: "Wetlands (inland)–Permanent/saline/brackish or alkaline marshes/pools"; 49: "Wetlands (inland)–Seasonal/intermittent/saline/brackish or alkaline marshes/pools"; 50: "Wetlands (inland)/Karst and other subterranean hydrological systems"; 51: "Rocky Areas"; 52: "Desert"; 53: "Desert–Hot"; 54: "Desert–Temperate"; 55: "Desert–Cold"; 56: "Marine–Neritic"; 57: "Marine–Neritic Pelagic"; 58: "Marine–Coral Reefs"; 59: "Marine–Seagrass (submerged)"; 60: "Marine–Oceanic"; 61: "Marine–Epipelagic"; 62: "Marine–Mesopelagic"; 63: "Marine–Bathypelagic"; 64: "Marine–Abyssopelagic"; 65: "Marine–Deep Ocean Floor"; 66: "Marine–Continental Slope/Bathyl zone"; 67: "Marine–Abyssal Plain"; 68: "Marine–Abyssal Mountains/Hills"; 69: "Marine–Hadal/Deep Sea Trench"; 70: "Marine–Seamounts"; 71: "Marine–Deep Sea Vent"; 72: "Marine–Intertidal"; 73: "Marine–Tidepools"; 74: "Marine–Mangroves submerged Roots"; 75: "Artificial–Terrestrial"; 76: "Arable land"; 77: "Pastureland"; 78: "Plantations"; 79: "Rural Gardens"; 80: "Urban Areas"; 81: "Subtropical/Tropical Heavily Degraded Former Forest"; and 82: "Unknown".


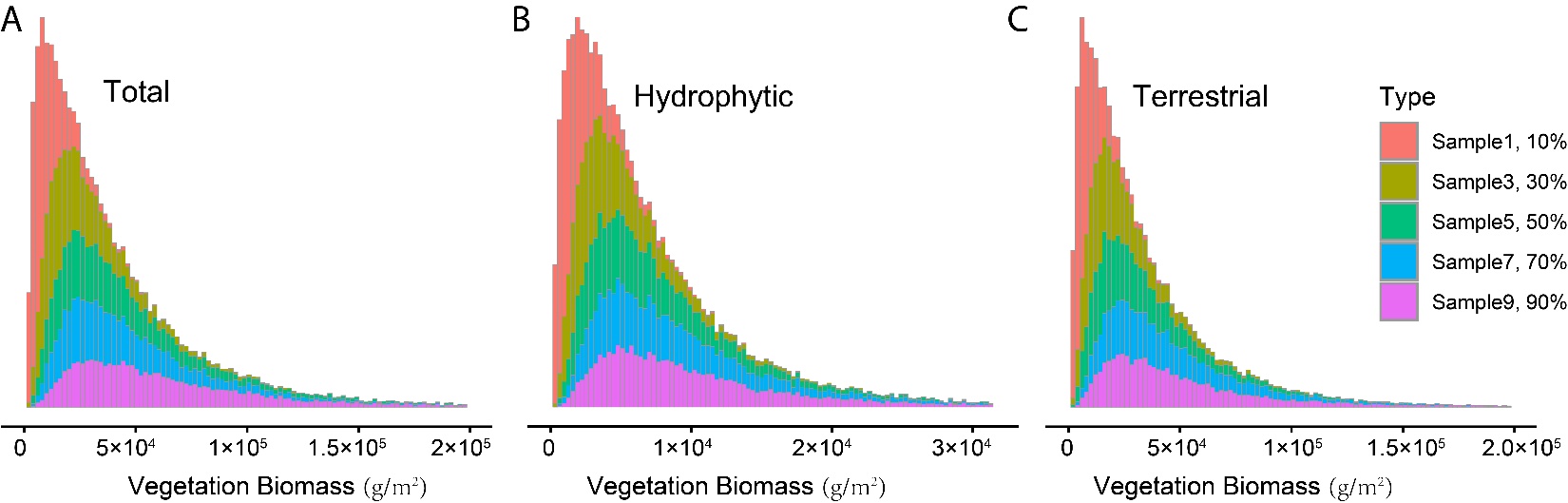


**Figure S3. Monte Carlo posterior distributions of reconstructed vegetation biomass (g m⁻²) derived from fossil insect assemblages under different sampling quantiles.** Panels show results for (A) total insect assemblages, (B) hydrophytic insects, and (C) terrestrial insects. Colored histograms represent vegetation biomass estimates based on insect biomass derived from the 10%, 30%, 50%, 70%, and 90% sampling quantiles of fossil assemblages from top to bottom. All distributions incorporate uncertainty from body mass estimation, density scaling, and trophic transfer parameters (feeding rate, accumulation rate, and transfer efficiency). Differences among quantiles illustrate the sensitivity of vegetation biomass reconstructions to fossil sampling structure and preservation bias, while the overall right-skewed distributions reflect the multiplicative propagation of ecological uncertainties.

**Graphical abstract**


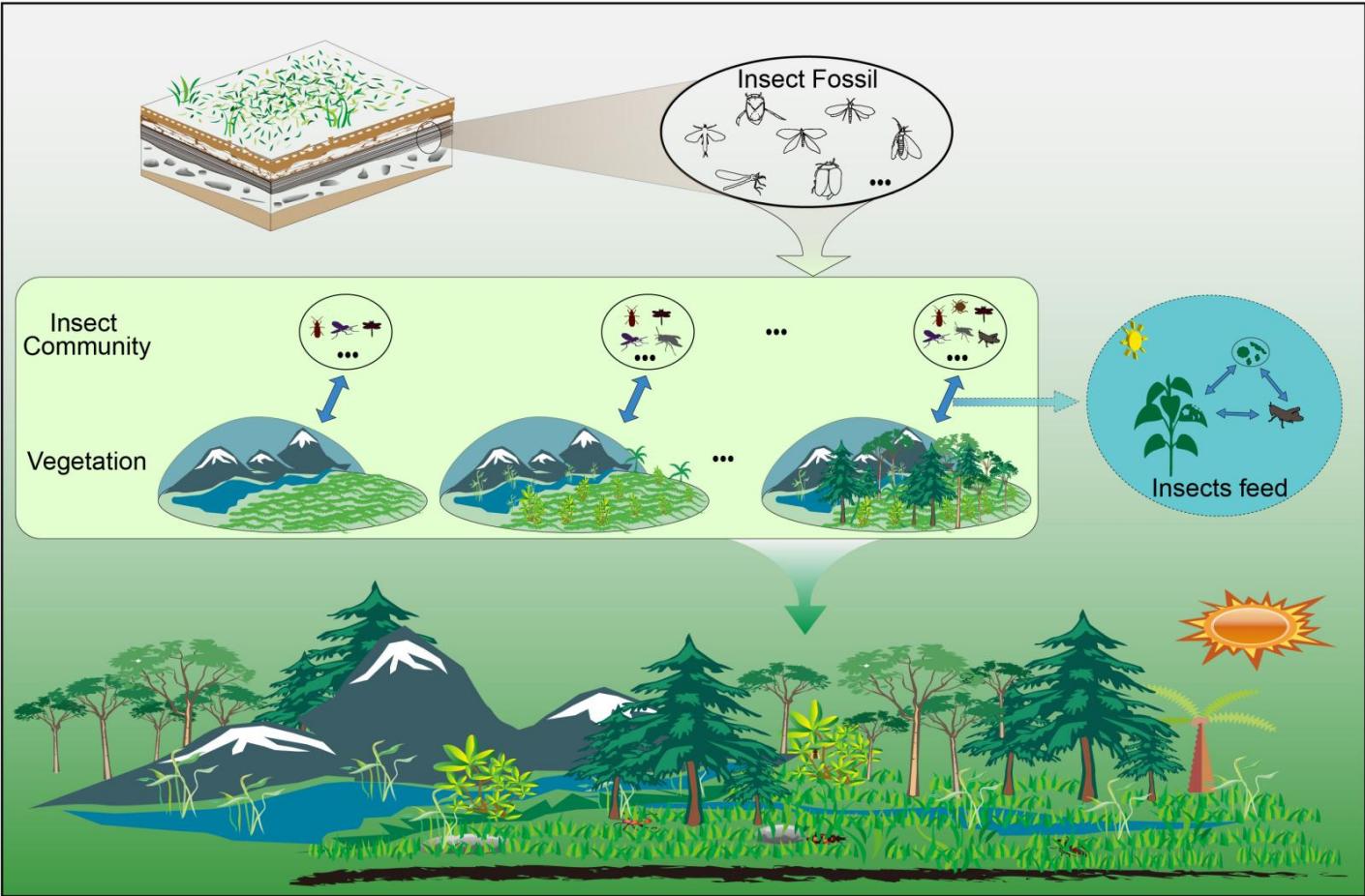


**References**

1. Ren D, Shih C, Gao T *et al.* *Rhythms of insect evolution: evidence from the Jurassic and Cretaceous in northern China*: John Wiley Sons Ltd, 2019.

2. Gao T, Shih C, Dong R. Behaviors and Interactions of Insects in Ecosystems of Mid-Mesozoic Northeastern China. *Annual Review of Entomology*. 2020; **66**. doi: 10.1146/annurev-ento-072720-095043

3. During MAD, Smit J, Voeten DFAE *et al.* The Mesozoic terminated in boreal spring. *Nature*. 2022; **603**(7899): 91–94. doi: 10.1038/s41586-022-04446-1

4. Sample BE, Cooper RJ, Greer RD *et al.* Estimation of insect biomass by length and width. *American Midland Naturalist*. 1993; **129**(2): 234–2240.

5. Jetz W, Carbone C, Fulford J *et al.* The Scaling of Animal Space Use. *Science*. 2004; **306**(5694): 266–268. doi: 10.1126/science.1102138

6. Damuth J. Interspecific allometry of population density in mammals and other animals: the independence of body mass and population energy-use. *Biological Journal of the Linnean Society*. 1987; **31**(3): 193–246. doi: <https://doi.org/10.1111/j.1095-8312.1987.tb01990.x>

7. Damuth J. Population density and body size in mammals. *Nature*. 1981; **290**(5808): 699–700. doi: 10.1038/290699a0

8. Hainsworth FR. Scaling: why is animal size so important *Auk*. 1985; **102**(3): 661–679. doi: 10.1093/auk/102.3.661

9. Gislason JC. Aquatic insect abundance in a regulated stream under fluctuating and stable diel flow patterns. *North American Journal of Fisheries Management*. 1985; **5**(1): 39–46.

10. Liu Mh, Li Y, Zhang Y *et al.* Correlation between Aquatic Insects and Environmental Factors in the Maoershan Streams. *Journal of Jilin Agricultural University*. 2014; **36**(4): 479–487.

11. Stagliano DM, Benke AC, Anderson DH. Emergence of aquatic insects from 2 habitats in a small wetland of the southeastern USA: temporal patterns of numbers and biomass. *Journal of the North American Benthological Society*. 1998; **17**(1): 37–53.

12. Zhanghe C, Hongta C, Bosun W. Studies on biomass and production of the lower subtropical evergreen broad-leaved forest in Heishiding Nature Reserve, China. VI. Distribution, biomass and production of roots. *Journal of Tropical Ecology*. 1994; **10**(2): 273–279.

13. Wint W. Leaf Damage in Tropical Rainforest Canopies. *Special Publication of the British Ecological Society*. 1983; **2**: 229–239.

14. Odum HT. *Summary: An emerging view of the ecological system at El Verde*. University of North Carolina, Chapel Hill: 1971 (Report Number(s): TID--24270)| (NSA Number: NSA-25-021639)|.

15. Guan D. Plant biomass and net primary production of myrtle bush communities in Hong Kong. *Chinese Journal of Acta Phytoecologica Sinica*. 1998; **22**(4): 356–363.

16. Gray JT. Community structure and productivity in *Ceanothus* chaparral and coastal sage scrub of southern California. *Ecological Monographs*. 1982; **52**(4): 415–434. doi: <https://doi.org/10.2307/2937353>

17. Whittaker RH, Woodwell GMJ. Dimension and production relations of trees and shrubs in the Brookhaven Forest, New York. *Journal of Ecol*ogy 1968; **56**: 1.

18. Lindeman RL. The trophic-dynamic aspect of ecology. *Bulletin of Mathematical Biology*. 1991; **53**(1): 167–191. doi: 10.1016/S0092-8240(05)80045-X

19. Lindeman RLJ. The trophic-dynamic aspect of ecology. *Ecology*. 1942; **23**(4): 399–417. doi: 10.2307/1930126

20. Levieux J. The soil fauna of tropical savannas. IV. The ants. *Ecosystems of the World.* 1983.

21. Potter D, DWB P, MA L. A study of the benthic macro-invertebrates of a shallow entrophic reservoir in South Wales with emphasis on the Chironomidae (Diptera); their life-histories and production. *Archiv für Hydrobiologie* 1974; **74**(2): 186–226.

22. Saito S. Energetics of isopod populations in a forest of central Japan. *Research on Population Ecology*. 1969; **11**(2): 229–258.

23. Crisp D. Estimates of the annual production of *Corixa germar*i (Fieb.) in an upland reservoir. *Archiv für Hydrobiologie* 1962; **58**(2): 210–223.

24. Mispagel ME. The Ecology and Bioenergetics of the Acridid Grasshopper, *Bootettix punctatus* on Creosotebush, *Larrea* *tridentata*, in the Northern Mojave Desert. *Ecology*. 1978; **59**(4): 779–788. doi: <https://doi.org/10.2307/1938782>

25. Mathias JA. Energy flow and secondary production of the amphipods *Hyalella azteca* and *Crangonyx richmondensis* *occidentalis* in Marion Lake, British Columbia. *Journal of the Fisheries Board of Canada*. 1971; **28**(5): 711–726.

26. Maitland PS, Hudspith PM. The zoobenthos of Loch Leven, Kinross, and estimates of its production in the sandy littoral area during 1970 and 1971. *Proceedings of the Royal Society of Edinburgh, Section B: Biological Sciences*. 1974; **74**: 219–239.

27. Block W. Seasonal fluctuations and distribution of mite populations in moorland soils, with a note on biomass. *The Journal of Animal Ecology*. 1966; **35**(3): 487–503.

28. Griffiths D. The structure of an acid moorland pond community. *The Journal of Animal Ecology*. 1973; **42**(2): 263–283.

29. Jacobs J. Coexistence of similar zooplankton species by differential adaptation to reproduction and escape in an environment with fluctuating food and enemy densities. *Oecologia*. 1977; **30**(4): 313–329.

30. McNeill S. The energetics of a population of *Leptopterna dolabrata* (Heteroptera: Miridae). *Journal of Animal Ecology*. 1971; **40**(1): 127–140.

31. Stachurski A. Population density, biomass and maximum natality rate and food conditions in *Ligidium hypnorum* L.(Isopoda). *Ekologia Polska*. 1972; **20**: 185–198.

32. Waters TF, Crawford GW. Annual production of a stream mayfly population: A comparison of methods 1. *Limnology and Oceanography*. 1973; **18**(2): 286–296.

33. Elliott J. The life cycle and production of the leech *Erpobdella octoculata* (L.)(Hirudinea: Erpobdellidae) in a Lake District stream. *Journal of Animal Ecology*. 1973; **42**(2): 435–448.

34. Jónasson PM. Ecology and production of the profundal benthos in relation to phytoplankton in Lake Esrom. *Oikos*. 1972; **14**: 1–148.

35. Babitskiy V. Biology and production of *Eurycercus lamellatus* (OFM) along the shores of Lake Narach. *Hydrobiological Journal*. 1970; **6**: 26–32.

36. Ricklefs RE. Adaptation, constraint, and compromise in avian postnatal development. *Biological Reviews*. 1979; **54**(3): 269–290. doi: <https://doi.org/10.1111/j.1469-185X.1979.tb01013.x>

37. Burky AJ. Biomass turnover, respiration, and interpopulation variation in the stream limpet *Ferrissia rivularis* (Say). *Ecological Monographs*. 1971; **41**(3): 235–251.

38. Hinton J. Energy flow in a natural population of *Neophilaenus lineatus* (Homoptera). *Oikos*. 1971; **22**(2): 155–171.

39. Beattie D, Golterman H, Vijverberg J. An introduction to the limnology of the Friesian lakes. *Hydrobiologia*. 1978; **58**(1): 49–64.

40. Smalley AE. Energy Flow of a Salt Marsh Grasshopper Population. *Ecology*. 1960; **41**(4): 672–677. doi: <https://doi.org/10.2307/1931800>

41. Sunderland K, Hassall M, Sutton S. The population dynamics of *Philoscia muscorum* (Crustacea, Oniscoidea) in a dune grassland ecosystem. *Journal of Animal Ecology*. 1976; **45**(2): 487–506.

42. Sutton S. The population dynamics of *Trichoniscus pusillus* and *Philoscia muscorum* (Crustacea, Oniscoidea) in limestone grassland. *Journal of Animal Ecology*. 1968; **37**(2): 425–444.

43. Gillespie DM. Population studies of four species of molluscs in the Madison River, Yellowstone National Park 1. *Limnology and Oceanography*. 1969; **14**(1): 101–114.

44. Eckblad JW. Population studies of three aquatic gastropods in an intermittent backwater. *Hydrobiologia*. 1973; **41**(2): 199–219.

45. Golley F, Gentry J. Bioenergetics of the Southern Harvester Ant, *Pogonomyrmex badius*. *Ecology*. 1964; **45**: 217. doi: 10.2307/1933834

46. Stockner JG. Ecological energetics and natural history of *Hedriodiscus truquii* (Diptera) in two thermal spring communities. *Journal of the Fisheries Board of Canada*. 1971; **28**(1): 73–94.

47. Brian M, Hibble J, Stradling D. Ant pattern and density in a southern English heath. *The Journal of Animal Ecology*. 1965; **34**(3): 545–555.

48. Welch H. Ecology of Chironomidae (Diptera) in a polar lake. *Journal of the Fisheries Board of Canada*. 1976; **33**(2): 227–247.

49. Phillipson J. Life cycle, numbers, biomass and respiratory metabolism of *Trichoniscus pusillus* (Crustacea, Isopoda) in a beech woodland—Wytham Woods, Oxford. *Oecologia*. 1983; **57**(3): 339–343.

50. Horst TJ, Marzolf GR. Production ecology of burrowing mayflies in a Kansas reservoir. *Internationale Vereinigung für theoretische und angewandte Limnologie: Verhandlungen*. 1975; **19**(4): 3029–3038.

51. Cooper WE. Dynamics and production of a natural population of a fresh-water amphipod, *Hyalella azteca*. *Ecological Monographs*. 1965; **35**(4): 377–394.

52. Anderson RO, Hooper FF. Seasonal abundance and production of littoral bottom fauna in a southern Michigan lake. *Transactions of the American Microscopical Society*. 1956; **75**(3): 259–270.

53. Duke K, Crossley Jr D. Population energetics and ecology of the rock grasshopper, *Trimerotropis saxatilis*. *Ecology*. 1975; **56**(5): 1106–1117.

54. Mason C. Snail populations, beech litter production, and the role of snails in litter decomposition. *Oecologia*. 1970; **5**(3): 215–239.

55. Jennings T, Barkham J. Slug populations in mixed deciduous woodland. *Oecologia*. 1975; **20**(3): 279–286.

56. Phillipson J, Abel R, Steel J *et al.* Earthworm numbers, biomass and respiratory metabolism in a beech woodland-Wytham Woods, Oxford. *Oecologia*. 1978; **33**(3): 291–309.

57. Burke MV, Mann K. Productivity and production: biomass ratios of bivalve and gastropod populations in an eastern Canadian estuary. *Journal of the Fisheries Board of Canada*. 1974; **31**(2): 167–177.

58. Rigler F, MacCallum M, Roff J. Production of zooplankton in Char Lake. *Journal of the Fisheries Board of Canada*. 1974; **31**(5): 637–646.

59. Hunter RD. Growth, fecundity, and bioenergetics in three populations of *Lymnaea palustris* in upstate New York. *Ecology*. 1975; **56**(1): 50–63.

60. Kajak Z, Rybak JI. Production and some trophic dependences in benthos against primary production and zooplankton production of several Masurian lakes. *Internationale Vereinigung für theoretische und angewandte Limnologie: Verhandlungen*. 1966; **16**(1): 441–451.

61. Kuenzler EJ. Structure and energy flow of a mussel population in a Georgia salt marsh 1. *Limnology and Oceanography*. 1961; **6**(2): 191–204.

62. Williamson P, Cameron R, Carter M. Population dynamics of the landsnail *Cepaea nemoralis* L.: a six-year study. *Journal of Animal Ecology*. 1977; **46**(1): 181–194.

63. Tudorancea C. Studies on Unionidae populations from the Crapina-Jijila complex of pools (Danube zone liable to inundation). *Hydrobiologia*. 1972; **39**(4): 527–561.

64. Kay D, Brafield A. The energy relations of the polychaete *Neanthes* (= *Nereis*) *virens* (Sars). *Journal of Animal Ecology*. 1973; **42**(3): 673–692.

65. Pearson WD, Kramer RH. Drift and production of two aquatic insects in a mountain stream. *Ecological Monographs*. 1972; **42**(3): 365–385.

66. Momot WT, Gowing H. Production and population dynamics of the crayfish *Orconectes virilis* in three Michigan lakes. *Journal of the Fisheries Board of Canada*. 1977; **34**(11): 2030–2040.

67. Abrahamsson SA, Goldman CR. Distribution, density and production of the crayfish *Pacifastacus leniusculus* Dana in Lake Tahoe, California-Nevada. *Oikos*. 1970; **21**(1): 83–91.

68. Paine RT. Size‐limited predation: an observational and experimental approach with the *Mytilus*–*Pisaster* interaction. *Ecology*. 1976; **57**(5): 858–873.

69. Lavelle P. Production annuelle d’un ver de terre *Millsonia anomala omodeo*. *Revue d'Ecologie, Terre et Vie*. 1971; **2**: 240–254.

70. Otto C. Energetic relationships of the larval population of *Potamophylax cingulatus* (Trichoptera) in a South Swedish stream. *Oikos*. 1975; **26**(2): 159–169.

71. Jónasson PM. Population ecology and production of benthic detritivores. *Internationale Vereinigung für theoretische und angewandte Limnologie: Verhandlungen*. 1975; **19**(2): 1066–1072.

72. Zervanos SM, Hadley NF. Adaptational biology and energy relationships of the collared peccary (*Tayassu tajacu*). *Ecology*. 1973; **54**(4): 759–774.

73. Hughes RN. An energy budget for a tidal-flat population of the bivalve *Scrobicularia plana* (Da Costa). *Journal of Animal Ecology*. 1970; **39**(2): 357–381.

74. Hughes RN. Population dynamics of the bivalve *Scrobicularia plana* (Da Costa) on an intertidal mud-flat in North Wales. *Journal of Animal Ecology*. 1970; **39**(2): 333–356.

75. Giani N, Laville H. Cycle biologique et production de *Sialis lutaria* L.(Megaloptera) dans le lac de Port-Bielh (Pyrenees Centrales). *Annales de Limnologie–International Journal of Limnology*. 1973; **9**(1): 45–61.

76. Klein G, Rachor E, Gerlach SA. Dynamics and productivity of two populations of the benthic tube-dwelling amphipod *Ampelisca brevicornis* (Costa) in Helgoland Bight. *Ophelia*. 1975; **14**(1-2): 139–159. doi: 10.1080/00785236.1975.10421973

77. Rigler F, Cooley J. The use of field data to derive population statistics of multivoltine copepods 1. *Limnology and Oceanography*. 1974; **19**(4): 636–655.

78. Miller R, Mann K. Ecological energetics of the seaweed zone in a marine bay on the Atlantic coast of Canada. III. Energy transformations by sea urchins. *Marine Biology*. 1973; **18**(2): 99–114.

79. Negus CL. A quantitative study of growth and production of unionid mussels in the River Thames at Reading. *Journal of Animal Ecology*. 1966; **35**(3): 513–532.

80. Resh VH. The use of transect sampling in estimating single species production of aquatic insects. *Internationale Vereinigung für theoretische und angewandte Limnologie: Verhandlungen*. 1975; **19**(4): 3089–3094.

81. Waters TF. Production Rate, Population Density, and Drift of a Stream Invertebrate. *Ecology*. 1966; **47**(4): 595–604. doi: <https://doi.org/10.2307/1933937>

82. Burgis MJ. Revised estimates for the biomass and production of zooplankton in Lake George, Uganda. *Freshwater Biology*. 1974; **4**(6): 535–541.

83. Mattice JS. Production of a natural population of *Bithynia tentaculata* L.(Gastropoda, Mollusca). *Ekologia Polska*. 1972; **20**(39): 525–539.
